# LC/MS analysis and deep sequencing reveal the accurate RNA composition in the HIV-1 virion

**DOI:** 10.1038/s41598-019-45079-1

**Published:** 2019-06-18

**Authors:** Anna Šimonová, Barbora Svojanovská, Jana Trylčová, Martin Hubálek, Ondřej Moravčík, Martin Zavřel, Marcela Pávová, Jan Hodek, Jan Weber, Josef Cvačka, Jan Pačes, Hana Cahová

**Affiliations:** 1 Institute of Organic Chemistry and Biochemistry of the Czech Academy of Sciences, Prague, 16610 Czech Republic; 2 First Faculty of Medicine, Charles University, Prague, 12108 Czech Republic; 3 Institute of Molecular Genetics of the Czech Academy of Sciences, Prague, 14220 Czech Republic; 4 University of Chemistry and Technology, Prague, 16628 Czech Republic

**Keywords:** RNA, Retrovirus

## Abstract

The mechanism of action of various viruses has been the primary focus of many studies. Yet, the data on RNA modifications in any type of virus are scarce. Methods for the sensitive analysis of RNA modifications have been developed only recently and they have not been applied to viruses. In particular, the RNA composition of HIV-1 virions has never been determined with sufficiently exact methods. Here, we reveal that the RNA of HIV-1 virions contains surprisingly high amount of the 1-methyladenosine. We are the first to use a liquid chromatography-mass spectrometry analysis (LC/MS) of virion RNA, which we combined with m^1^A profiling and deep sequencing. We found that m^1^A was present in the tRNA, but not in the genomic HIV-1 RNA and the abundant 7SL RNA. We were able to calculate that an HIV-1 virion contains per 2 copies of genomic RNA and 14 copies of 7SL RNA also 770 copies of tRNA, which is approximately 10 times more than thus far expected. These new insights into the composition of the HIV-1 virion can help in future studies to identify the role of nonprimer tRNAs in retroviruses. Moreover, we present a promising new tool for studying the compositions of virions.

## Introduction

To date, more than 140 chemical RNA modifications are known^1^. Chemical RNA modification expand the repertoire of four natural nucleosides and influence RNA structure and function. The majority has been found in highly concentrated RNA moieties such as ribosomal (rRNA) or transfer RNA (tRNA). Nevertheless, there is growing evidence that coding messenger RNA (mRNA) and regulatory RNA also contain various chemical modifications^2^. Their existence in mRNA is complicated to be proved by common LC/MS technique but special and selective profiling or capturing techniques for various RNA modification have been developed lately. In particular, pseudouridine has been found in the mRNA thanks to the development of the selective profiling techniques Pseudo-seq.^3^ and Ψ-seq.^4^. Also, the widespread presence of 5-methylcytosine across the human transcriptome has been demonstrated by a bisulphite technique combined with deep sequencing^5^. A to I editing of the mRNA has been confirmed by Inosine chemical erasing^6^. The NAD captureSeq.^7^ allowed identification of RNAs with covalently attached Nicotinamide adenine dinucleotide (NAD) at 5′ end. So far, this method has enabled the detection of NAD in sRNA, and mRNA in *E. coli*^7^ and in eukaryotes^8,9^.

The development of a profiling technique for 6-methyladenosine (m^6^A) in mRNA^10,11^ paved the way for the emerging field of epitranscriptomic. Another common epitranscriptomic mark in mRNA was supposed to be 1-methyladenosine (m^1^A)^12,13^. As well as for m^6^A detection the specific antibodies have been used to detect m^1^A in the human transcriptome. The problem of immunoprecipitation methods is that they do not allow for single base resolution of particular RNA modification. The authors claimed identification of thousands of transcripts bearing m^1^A in eukaryotic cells. A subsequent study indicated that the m^1^A is only present in 15 sites of the mRNA, in the previously known T-loop of tRNAs, and in rRNA from HEK293T cells^14^. Apart from immunoprecipitation, the authors of the later method employed two types of reverse transcriptases enabling single-base resolution.

The study of the chemical structures of RNA species such as mRNA, micro RNA and guiding RNA is hampered by their very low concentrations in cells. Therefore, we believe that viruses may represent a suitable model system for RNA modification studies because they have simple intrinsic structure and well-known molecular mechanisms.

So far, m^6^A profiling is the only applied technique for the studies of viral RNA modifications. Recently, m^6^A profiling has been used to study m^6^A in the genomic RNA of *Flaviviridae*^15^. In particular, the conserved regions modified by m^6^A were identified in genomic RNA of hepatitis C (HCV), dengue, Zika, yellow fever and West Nile virus. The m^6^A modulated infection by HCV and the depletion of m6A methyltransferases or m6A demethylase was found to increase or decrease the HCV particle production, respectively. A photo-crosslinking-assisted m^6^A sequencing technique was used to precisely map m^6^A editing sites on the HIV-1 genome^16^. The m^6^A sites were found to cluster in the HIV-1 3′ untranslated region and to recruit YTHDF “reader” proteins. The m^6^A editing and the recruitment of YTHDF proteins were identified as the positive regulators of HIV-1 mRNA expression.

As new layers of RNA modifications are constantly being uncovered in the transcriptome, we can presume that other RNA modifications besides m^6^A can be employed by viruses. At first, we focused on the viral packageome of HIV-1. It was believed that HIV-1 packs in virion not only two copies of genomic RNA, but also 12–14 copies of guiding 7SL RNA^17,18^, 8 copies of tRNA^Lys3^ that serve as primer for its reverse transcriptase^19–21^, around 12 copies of tRNA^Lys1,2^, around 50 copies of rather randomly packed tRNAs and some fragments of other mRNA, rRNA etc^18,22–26^. In total, the host cell-derived RNA should comprise up to half of the virion RNA by mass^27,28^.

To best of our knowledge, the LC/MS analysis of RNA from viral particles has not been reported yet. We isolated RNA from HIV-1 viral particles, digested it into form of nucleosides and we analysed this mixture by LC/MS system. We found surprisingly high amount of m^1^A (4.1% of all adenosines - A) in the packageomic RNA that corresponded to approximately 340 1-methyladenosines (Supplementary Table S1). The tRNA molecules contain one m^1^A in their sequence, what correlates to 70 positions. In the light of the emerging field of epitranscriptomics we asked the question where the rest 270 m^1^A can come from and whether the genomic HIV-1 RNA or other co-packed RNA are so heavily methylated. Therefore, we prepared four deep sequencing libraries with particular focus on m^1^A profiling. We found that the m^1^A is present solely in tRNA and that genomic HIV-1 RNA, neither 7SL RNA do not contain this modification. Based on this results we could recalculate the ratios of genomic HIV-1 RNA, 7SL RNA and tRNAs in the virion. We concluded that the HIV-1 virion consists beside 2 copies of genomic HIV-1 RNA and 14 copies of 7SL RNA also 770 copies of tRNA. Our results suggest the role for the cellular RNA could be much more significant than expected so far, both in mechanism of viral infection and in viral replication. Our study also confirms the evidence that the m^1^A modification is rather typical for tRNAs^14^ and it is not so widespread as it was previously described^12^. We also show here that the LC/MS analysis of RNA modifications in virion in combination with deep sequencing can be used for the determination of the exact RNA composition of complex virions. The presence of specific RNA modification m^1^A helped us to bring new insight into the HIV-1 virion composition. In future, this method can be applied also for studies of the composition of other viruses.

## Results

### LC/MS technique reveals a higher amount of m^1^A in the HIV-1 packageome than expected

**Figure 1:**
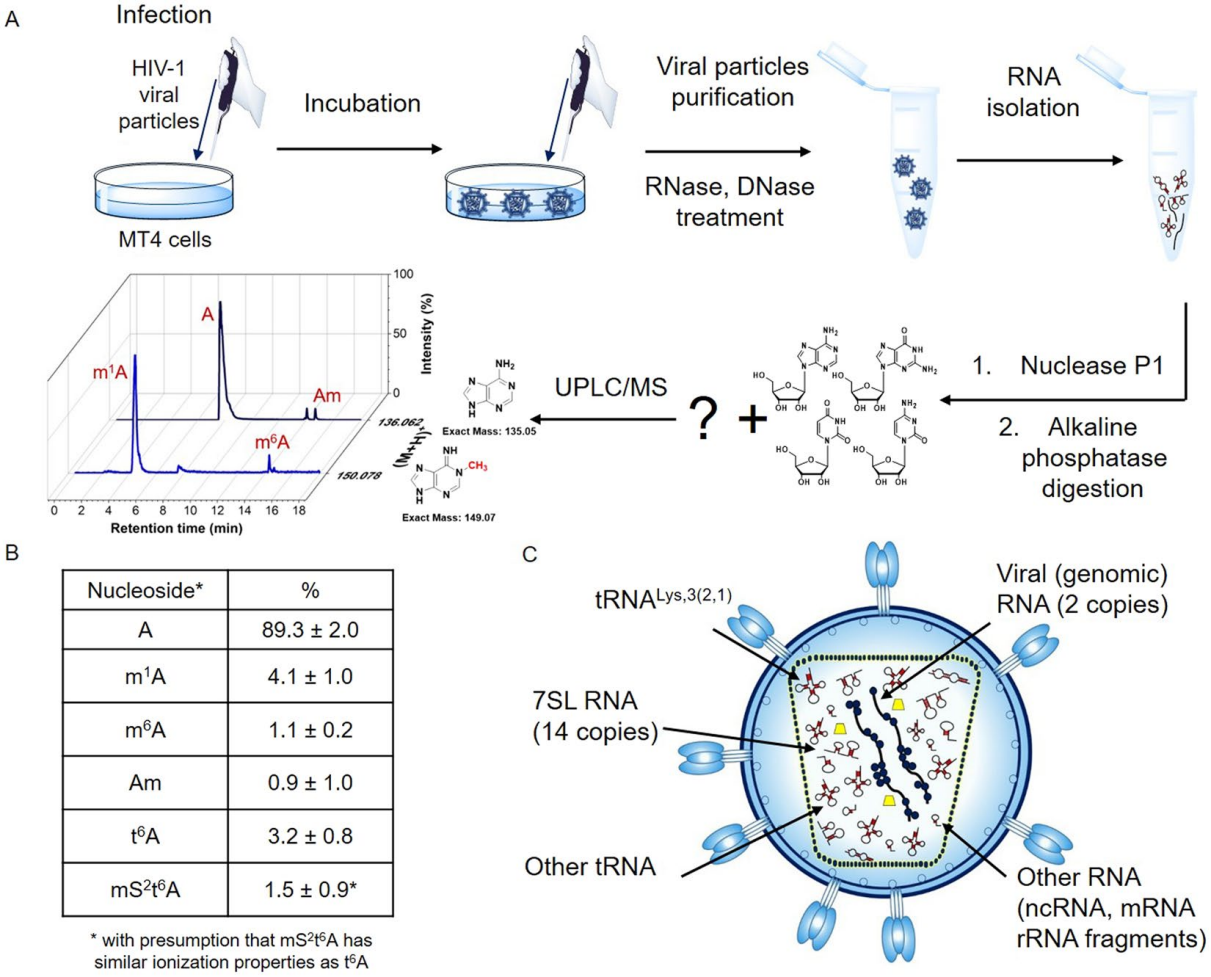
LC/MS analysis of RNA in the HIV-1 packageome. (**A**) Scheme showing the preparation of HIV-1 samples for LC/MS analysis. (**B**) Ratios of nucleosides in the HIV-1 samples as analysed by LC/MS using biological tetraplicates. (**C**) A schematic view on the RNA composition in HIV-1.

In order to analyse the RNA modifications in the HIV-1 virus, we firstly produced HIV-1 virus by infection of MT4 cells (Fig. 1A). We harvested the supernatant both from the infected cells and the uninfected MT4 cells, and we used the latter as the mock medium. After purification of the virions on sucrose cushion, we treated the sample by RNase and DNase to digest the nucleic acids not packed in viral particles. Then, the RNA was isolated by RNAzol. The samples were precipitated using ethanol and then digested by Nuclease P1 and Alkaline Phosphatase to form nucleosides. The digested RNA was analysed using a Synapt G2 LC/MS system (Fig. 1A,B). The authenticity of the methylated nucleosides was confirmed by injection of synthetic standards, which produced the same *m/z* signal and were eluted at the same time.

Using LC/MS analysis, we observed methylated adenosines. m^6^A was present only in low amount approx. 1.1%. Surprisingly, we found quite high amount of m^1^A around 4.1% of all adenosines in the packageome of the HIV-1-virus. We also searched for other known modified adenosines in the RNA digest and we were able to confirm only N^6^-threonylcarbamoyladenosine (t^6^A), 2′-O-methyladenosine (Am) and 2-methylthio-N^6^-threonylcarbamoyladenosine (mS^2^t^6^A) (Supplementary Figures S1, S2). Other modified adenosines were not detected by our LC/MS analysis or they were present only in traces that we could not calculate (2-methylthio-N^6^-isopentenyladenosine, N^6^-(cis-hydroxyisopentenyl)adenosine, N^6^-isopentenyladenosine, Supplementary Table S2^29^). Analysing the synthetic standards of m^6^A, m^1^A, Am and t^6^A, we were able to extrapolate their proportional representation in the digest (Supplementary Figures S1, S2, Table S3). As we did not have available standard of mS^2^t^6^A, we presume that the molecule has similar ionization properties as t^6^A. The packageomic RNA contains 4.1% of m^1^A, 1.1% of m^6^A, 0.9% of Am, 3.2% of t^6^A and 1.5% of mS^2^t^6^A (rest is A).

To verify that the signals of modified nucleosides originate from RNA molecules packed in viral particles, we analyzed the medium of the uninfected cells by LC/MS analysis (Supplementary Table S4). Here, we did not detect any traces of m^1^A or other modified A. Thus, we conclude that the signals of modified nucleosides in the samples prepared from supernatant of HIV-1 infected cells stem indeed from RNA molecules packed in viral particles. As the RNAzol protocol only isolates oligonucleotides longer than 10 nt, we can rule out the possibility that the modified nucleoside signal originates from co-packed small molecules.

Using previously published data on the number of co-packed RNA entities in one particle (two genomic HIV-1 RNAs, 14 7SL RNAs, approx. 70 tRNAs, Fig. 1C)^18,22,23^ and previous evidence on the presence of m^1^A in position 58 of the majority of tRNAs, we conclude that in RNA packed in a viral particle, 270 positions would be methylated. However, this number did not correspond to the previous estimate of approximately 70 tRNA molecules.

### m^1^A is solely present in the tRNA

To investigate the exact positions of m^1^A in the packageomic RNA, we prepared a deep sequencing library. As the viral packageome is very simple, we used for the m^1^A localisation the method based on the reverse transcription signature^30–32^. The RNA for deep sequencing was prepared in biological triplicates. Every sample was divided in two parts and one part was treated by basic conditions to obtain an m^1^A conversion to m^6^A (Dimroth rearrangement)^12^ that is normally read as A by reverse transcriptase. We prepared three deep sequencing libraries using SuperScript^TM^ III reverse transcriptase, which should either misread m^1^A or arrest meeting m^1^A. One deep sequencing library was prepared with TGIRT^TM^ reverse transcriptase, which should confirm the m^1^A position at base resolution.

**Figure 2:**
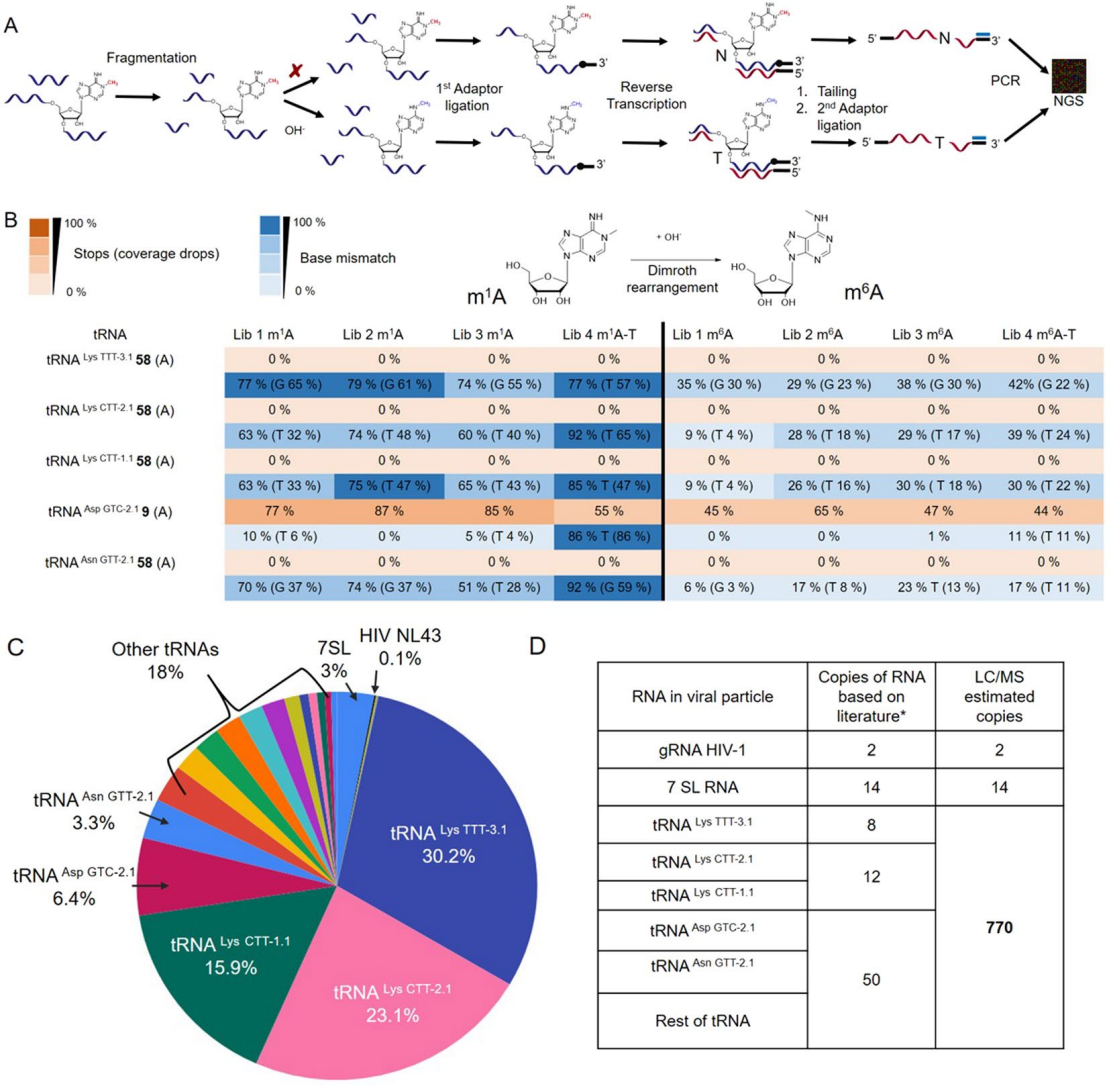
m^1^A profiling of RNAs in HIV-1 virion. (**A**) Scheme showing the preparation of the deep sequencing libraries of RNA isolated from HIV-1 virions. (**B**) Table showing the observed misincorporation (blue) or arrests (orange) of reverse transcriptase in the deep sequencing libraries. T stands for library Lib4 prepared with TGIRT reverse transcriptase, while Lib1-Lib3 libraries were prepared with SuperScript^TM^ III reverse transcriptase. (**C**) The percentages of the most abundant RNA reads in the deep sequencing libraries. (**D**) Table showing the extrapolated numbers of copies of RNAs in HIV-1 virion based on literature (∗)^17,18^ and on our LC/MS data.

In the deep sequencing protocol (Fig. 2A), we used a sequence of the following steps: The RNA was first fragmented to obtain fragments of uniform lengths (approx. 100 nt), then ligated with the first adaptor, reverse transcribed, tailed by cytidine triphosphate (CTP) and finally ligated with the second adaptor. After PCR with indexed primers, the samples were separated using agarose gel electrophoresis. The cut off was approximately 100–350 nt including the adaptors. Afterwards, the samples were pooled in a library and sequenced by IonTorrent technology.

The bioinformatics analysis confirmed that the virion contains mainly tRNAs, 7SL RNA and HIV-1 genomic RNA (Fig. 2C, Supplementary Table S5). Any other cellular RNAs were not detected in significant amount (more than 0.1%). By analysing the deep sequencing data using bioinformatics we identified m^1^A in position 58 of tRNA^Lys TTT-3.1.^, tRNA^Lys CTT-2.1.^, tRNA^Lys CTT-1.1.^ and tRNA^Asn GTT-2.1.^, as well as in position 9 of tRNA^Asp GTC-2.1.^. Based on a previous report^30^, we expected the reverse transcriptase to be paused (observed arrest) when meeting m^1^A. However, pattern of misreading for m^1^A in artificial sequences read by reverse transcriptase was also reported^30,32^. We observed this behavior of reverse transcriptase in the position 58 of some tRNAs. Approximately 70% of the m^1^A in position 58^33,34^ was misread (Fig. 2B – blue colour) as G or T by both reverse transcriptases used (SuperScript^TM^ III – library 1–3 and TGIRT^TM^ – library 4). In the sample that was treated with basic conditions, the misreading of position 58 was only approximately 30% (Fig. 2B). This observation corresponds to the expected conversion of m^1^A to m^6^A in the RNA. It also proves the chemical authenticity of m^1^A because the presence of other modified A would cause misincorporation but the ratio of misreading would not change after basic treatment.

Surprisingly, m^1^A in the position 9 of tRNA^Asp GTC-2.1.^ caused rather arrest (Fig. 2B – orange colour) of SuperScript^TM^ III in approximately 85% and a misreading as T (86%) was observed only in the experiment with TGIRT^TM^. In order to verify that the m^1^A profiling method works in our hand also when long RNAs are used, we tested the protocol on 28S rRNA from HEK293T cells. In this case, we observed arrest of the reverse transcriptase meeting m^1^A in position 1317 in 80%. This is contrary to the reverse transcriptase TGIRT^TM^, which misread the m^1^A mainly as G in 28S rRNA (Supplementary Table S7). Our observation shows that the reverse transcriptase behavior depends strongly not only on type of RNA modification but also on sequence and 2D structure (loop, bulge etc.). In any case, the behaviour of both reverse transcriptases (either arrest or misreading) always proved the presence of m^1^A.

Once we confirmed the m^1^A in tRNAs co-packed in viral particle, we searched for other m^1^A in all virion RNA entities. Adenosine is reported to be methylated in position 1 mainly within the specific motif GUUCN**A**NNC^14^ or similar. Therefore, we looked for i) the misreading pattern, ii) the arrests of reverse transcriptase, and iii) a similar motif to GUUCN**A**NNC (allowing one alteration) in 7SL RNA and the genomic HIV-1 RNA (Supplementary Table S7). Although we found the sequence motif allowing one alteration several times in the genomic RNA of HIV-1, we never observed a significant misreading or arresting pattern of reverse transcriptase meeting A in whole genomic RNA of HIV-1. 7SL RNA contains also one sequence motif but neither there we did not observe any significant misreading or arresting pattern. Because there are no other abundant RNA molecules beside tRNAs, (Supplementary Table S5), we concluded that all LC/MS detected m^1^A originates from the co-packed tRNAs.

To exclude the possibility that the used HIV-1 particles were not sufficiently purified on the sucrose cushion and the detected RNA, thus, came from extracellular vesicles^35^, we purified the viral particles using OptiPrep density gradient. The RNA prepared from OptiPrep density gradient purified virus represented 96% of RNA from sucrose cushion purified virus. The RNA from fractions removed by OptiPrep density gradient contained 1000 x less HIV-1 genomic RNA (Supplementary Figure S3) and we did not observe any tRNAs using TapeStation analysis (Supplementary Figure S4A).

Based on the deep sequencing data, we can conclude that the viral particle contains more than 96% of host RNA represented mainly by tRNAs. We estimate that tRNA^Lys TTT-3.1^ is present in 30%, tRNA^Lys CTT-2.1^ in 23%, tRNA^Lys CTT-1.1^ in 16%, tRNA^Asp GTC-2.1^ in 6% and tRNA^Asn GTT-2.1^ in 3% (Fig. 2C).

Precision of mapping is in almost all cases >99% (Supplementary Figure S5). However, the determined percent was averaged over all libraries and suffer from rather high variability. We are also aware of that e.g. PCR cycles can introduce a certain bias by preferentially amplifying short sequences over long ones.

### m^1^A stems from full-length tRNAs

**Figure 3:**
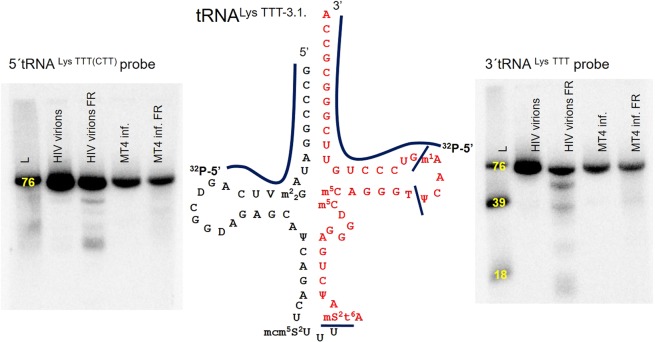
Northern blot analysis of RNA purified from HIV virions before and after fragmentation (FR) and from MT4 cells infected (inf.) with HIV-1 before and after fragmentation. The radioactively labelled probes were used for 5′ (left panel) and 3′ (right panel) ends of tRNA^Lys TTT-3.1.^. The yellow numbers stand for the size of synthetically prepared ladder (L) with the same sequence as natural tRNA^Lys TTT-3.1.^.

In our deep sequencing data, we only observed the 3′ half of the tRNA^Lys TTT-3.1^. We hypothesised that this phenomenon can either be explained by the presence of a bulky RNA modification such as mS^2^t^6^A at the anticodon loop or by the fact that the viral particle only contains the 3′-tRNA half of the tRNA^Lys TTT-3.1^. To shed light on this, we designed a northern blot analysis of the tRNA fragments. Radioactively labelled probes were designed for the 3′ ends of tRNA^Lys TTT-3.1^ and tRNA^Lys CTT-1.1 (2.1)^ as well as for the 5′ end of tRNA^Lys TTT-3.1^. As the 5′ end of tRNA^Lys TTT-3.1^ and tRNA^Lys CTT-1.1 (2.1)^ is similar, the 5′ end probe hybridize to all three tRNAs. We also prepared ladder with the sequence of tRNA^Lys TTT-3.1^ by *in vitro* transcription. The ladder helped us to estimate the size of observed bands. To rule out that the observed fragment in the deep sequencing data was caused by the fragmentation step, we analysed the RNA isolated from the viral particles and from the MT4 cells infected by HIV-1 before and after fragmentation. We observed the presence of 3′-tRNA fragments only in the RNA from viral particles after fragmentation (Fig. 3). Therefore, we ruled out the possibility that such 3′-tRNA fragments can contribute to the high level of m^1^A. Apparently, the observed fragments were formed during the preparation of the sample and they are not naturally present in the virion. We thus conclude that m^1^A solely originates from full-length tRNAs.

Nevertheless, the amount of observed m^1^A did not correspond to the number of co-packed tRNAs of 70 copies total in viral particle previously published^24–26^. Hence, we come to an end that the amount of co-packed tRNA is approximately 10-fold higher than previously reported. Based on the direct method of LC/MS analysis we can calculate that per 2 copies of genomic HIV-1 RNA and 14 copies of 7SL RNA HIV-1 virion contains also 770 copies of tRNAs (Fig. 2D, Supplementary Figure S6).

## Discussion

The RNA in the packageome of the HIV-1 virus was believed to consist of 2 copies of genomic HIV-1 RNA, 14 copies of 7SL RNA and 70 copies of tRNAs. Nevertheless, so far any direct method has not been used to study the RNA composition of virions. As first, we used LC/MS to study chemical composition of packageomic RNA. Surprisingly high amount of m^1^A led us to the deep sequencing analysis with particular focus on m^1^A. Even though immunoprecipitation methods for mapping m^1^A in complete transcriptome have been published^12,14^, we presumed that the method based on the reverse transcription signature would be sufficient for the localisation of m^1^A in simple packageome^30,31^. m^1^A profiling confirmed presence of this modification in tRNAs but excluded its presence in other RNA entities co-packed in HIV-1 virion. Once we ruled out the possibility that the m^1^A can stem from tRNA fragments, we were able to recalculate ratios of RNA molecules in HIV-1 virion. Based on LC/MS analysis, we extrapolate that the virion contains per 2 molecules of genomic HIV-1 RNA and 14 copies of 7SL RNA another 770 copies of various tRNAs.

So far, only one work has dealt with a deep sequencing analysis of HIV-1 particles^24^. According to Eckwahl *et al*., the HIV-1 particle contain even less than 8 copies of tRNA^Lys TTT-3.1^, as was previously reported. However, Eckwahl *et al*. admit that an absence of tRNA reads in their analysis can be caused by fragmentation that would lead to fragments <50 nt. Fragmented RNA would be underrepresented in their cDNA. In our deep sequencing libraries, however, we included RNAs with a size of around 40 nt. We, therefore, did not lose those short reads. In particular, we found that tRNA^Lys TTT-3.1.^ caused an almost quantitative abortion of the reverse transcriptase because it contains the bulky mS^2^t^6^A^36^ in position 37. As a result, the read had a length of only 39 nt.

Our deep sequencing analysis suggests that the HIV-1 virion contains approximately 69% of three versions of tRNA^Lys^. This is in agreement with works published by Kleiman *et al*. who found the ratio to be about 60%^37,38^. The same group later demonstrated using both microarray and 2D Page methods that the amount of all tRNA^Lys^ is only about 45%^39^. The reason for this difference may be imperfect labelling as both methods relied on ligation reactions.

The estimations of other co-packed tRNAs (50 molecules per virion)^18^ were based on the fact that the host non-coding RNAs in retroviruses outnumber viral gRNA by a factor of at least 50^40^.

In other works, the quantification of further tRNAs co-packed by an HIV-1 virion was usually omitted. Based on the knowledge that every tRNA contains one m^1^A in its sequence, we were able to quantify the number of all tRNAs in the viral packageome to be around 770. It is, however, important to mention that others often studied viral particles produced from different cells such as COS-7 cells^37,38^ or CEM-SS cells^40^, or produced by transfection and not infection^22^. Those factors can influence the composition of viral particles.

A recent report on RNA modifications in viral RNA that was purified via selective probes indicates that the genomic RNA of HIV-1 can contain besides m^1^A, 35 other RNA modifications^41^. The authors, however, conclude this fact only based on MS data, which means that the samples could be contaminated with heavily modified tRNA molecules. tRNA^Lys TTT-3.1.^, for example, can be bound so tightly to gRNA that it is not possible to separate the pure genomic RNA. Another error could have been introduced in the preparation of the viral samples. As the authors used only transfection, the plasmid containing the HIV-1 genomic sequence could have been fully transcribed into RNA. Based on our screening of HIV-1 sequence, we know that the additional plasmid sequence pNL4-3 contains the original motif GUUCNANNC, which might be methylated. However, we cannot exclude that a certain position in the genomic HIV-1 is methylated from less than 5% as our profiling technique would not be able to track it. However, this amount of m^1^A would not significantly contribute to the observed LC/MS signal. It is also arguable, whether such level of methylation would have any biological significance.

In our work, we also considered the existence of 3′ tRNA fragments that could contribute to observed high amount of m^1^A in LC/MS analysis. Pyrosequencing and recently also deep sequencing methods of small non-coding RNAs in HIV-1 infected cells have proven the existence of a very abundant 18-nt tRNA fragment from tRNA^Lys TTT-3.1.^ that is complemental to the HIV-1 primer binding site^42,43^. Rather than being a primer of reverse transcription, this tRNA fragment when overexpressed led to a decrease in viral replication. Nevertheless, the concentration of these tRNA fragments was so low that we were neither able to observe the fragments in isolated RNA from infected cells nor in packageomic RNA. Other types of 3′ tRNA fragments were observed only in Northern blot analysis of fragmented RNA. Therefore, we also ruled out the possibility that m^1^A can come from any type of tRNA fragments.

As the role of primer tRNA^Lys TTT-3.1.^ is quite well understood, the function of nonprimer tRNAs such as tRNA^Lys CTT-2.1.^, tRNA^Lys CTT-1.1.^, tRNA^Asp GTC-2.1.^ or tRNA^Asn GTT-2.1.^ is still very enigmatic. One of the hypothesis suggests that the matrix domain of the HIV-1 Gag protein binds almost exclusively to specific tRNAs in the cytosol^44^. tRNAs shield the membrane-binding surface of matrix and regulate the interaction with intracellular membranes prior reaching the plasma membrane^27^. Cellular tRNAs might also help to import HIV-1 intracellular reverse transcription complex to nucleus^45^. Another theory suggests that the enrichment of particular tRNAs in virion can manipulate with tRNA pool in cells and enhance production of the Gag polyprotein and virus production in general^46^. In any case, the importance of nonprimer tRNAs in virion is very high. In this work, we bring new tool – LC/MS in combination with deep sequencing –to study the virion RNA composition. Specific RNA modifications such as m^1^A can bring us missing information layer and in future, we can calculate the RNA composition of other viruses. Moreover, these techniques can be used for studies of an impact of viral infection on various RNA modification in host transcriptome.

## Methods

### Cell culture, transfections and infections

The human CD4 + T-cell line MT4 (NIH AIDS Reagent Program, Division of AIDS, NIAID, NIH from Dr. Douglas Richman) and the human embryonic kidney HEK293T cell line (American Type Culture Collection, LGC Standards, UK) were cultured under standard conditions at 37 °C under a humidified (>90%) atmosphere of 5% CO_2_/95% air. The MT4 cell line was cultured in RPMI 1640 w/o L-Glutamine and 25 mM Hepes supplemented with 10% FBS, penicillin (100 U/mL) and streptomycin (100 µg/mL) (all Sigma-Aldrich). The HEK293T cell line was cultured in DMEM high glucose supplemented with 10% FBS, penicillin (100 U/mL) and streptomycin (100 µg/mL) (all Sigma-Aldrich).

#### Isolation of large and small RNA fractions from the cell culture

MT4 cells were collected by centrifugation (225 × g, 5 min, and 20 °C), HEK293T cells were collected by trypsinization and subsequent centrifugation as above. Cells pellets were washed with PBS and cells were lysed with RNAzol reagent (Sigma-Aldrich). Large and small RNA fractions were purified according to the RNAzol manufacturer’s protocol. The RNA concentration was determined on NanoDrop ONE (ThermoFisher Scientific) and the RNA sample quality control was performed on a 4200 TapeStation System (Agilent).

#### Infection

MT4 cells were initially infected with a cell-free HIV-1 strain NL4-3, which was generated by transient transfection of HEK293T cells with a pNL4-3 plasmid (obtained through NIH AIDS Reagent Program, Division of AIDS, NIAID, NIH from Dr. Malcolm Martin). The infected cultures were subsequently expanded by co-cultivation. 48 h post-infection, cell culture supernatants containing viral particles and infected cells were added to uninfected MT-4 cells (5*10^5^ cells per mL) at a ratio of 1:9. The co-culture was synchronized by three successive additions of infected culture supernatant to uninfected MT4 cells (5*10^5^ cells per mL, the ratio of 1:9, 27 h between infections).

Virus-containing supernatants were harvested 40 h after the last synchronization step, cleared by centrifugation (225 × g, 5 min, 20 °C) and filtration (0.45 µm pore size cellulose-acetate filter (VWR)), and stored at −80 °C.

Infectious titres were determined as 50% tissue culture infectious dose by endpoint titration using serial 10-fold dilutions of the virus on TZM-bl cells^47^.

### Viral particles purification and RNA isolation

#### Sucrose cushion isolation

Virus particles were concentrated from cleared culture medium by centrifugation through a cushion of 20% (wt./wt.) sucrose in phosphate-buffered saline (PBS) (90 000 × g, 90 min, 4 °C). The pellet was resuspended in RNase/DNase buffer (Tris HCl 100 mM, MgCl_2_ 25 mM, CaCl_2_ 25 mM) with DNase I (10 U/mL, New England BioLabs - NEB), RNase I (200 U/mL) and RNase A (20 mg/mL, both ThermoFisher Scientific) and incubated 2 h at 37 °C. RNase/DNase treatment was stopped by adding RNAzol (Sigma-Aldrich).

Mock medium was prepared in the same way from uninfected MT-4 cells.

#### OptiPrep gradient isolation

Virus particles were concentrated and cleared through a sucrose cushion as described above. The virus pellet was resuspended in PBS, loaded to the top of the iodixanol gradient (6% to 35% OptiPrep Density Gradient medium diluted in PBS, Sigma–Aldrich) and ultracentrifuged (90 000 × g, 90 min, 4 °C). HIV-containing fractions were detected by western blot analysis using the anti-HIV capsid protein antibody. Selected fractions were collected, diluted with PBS five times and the virus was pelleted by ultracentrifugation (90 000 × g, 45 min, 4 °C). The virus-containing pellet was resuspended in RNase/DNase buffer and RNase/DNase treatment was performed as described above.

#### Western blot analysis

Samples from each fraction of the Optiprep gradient were mixed with loading buffer in the ratio of 5:1 and denatured for 5 min at 95 °C. Separation was carried out at 15% SDS PAGE at constant voltage 150 V for 90 min. Separated proteins were transferred to polyvinylidene difluoride (PVDF) membrane (Bio-Rad) and blocked at casein (Blocker Casein, ThermoFisher Scientific). HIV-1 CA protein was detected with polyclonal anti HIV-1 CA antibody (sera of rabbit immunized with purified HIV-1 CA protein, produced at Institute of Physiology CAS; dilution 1:1000, 1 h, RT) and as secondary antibody was used Goat anti Rabbit IgG labelled with horseradish peroxidase (1:10000, 1 h, RT, Sigma-Aldrich). After washing, the membranes were incubated with SuperSignal West Femto Maximum Sensitivity Substrate (ThermoFisher Scientific) and the intensity of chemiluminescence was detected using the CCD camera (Las-3000, software Image Reader Las-3000, Fujifilm) (Supplementary Figure S7).

RNA from viral particles was purified by Zymo-Spin™ IIC Columns (Direct-zol™ RNA MiniPrep Plus, Zymo) according to the manufacturer’s protocol. The quality of RNA samples was checked by HS RNA ScreenTape. (Supplementary Figure S4) RNA samples were quantified by an RNA High Sensitivity Assay (Quibit 4 Fluorometer, ThermoFisher Scientific), and the presence of HIV gRNA was confirmed by RT-PCR (LightCycler, Roche) (Supplementary Figure S3).

### RNA digestion and LC/MS analysis

RNA samples (1–4 µg) were fully digested by Nuclease P1 (1 U/µg of RNA, Sigma-Aldrich) in 40 µL of 50 mM ammonium acetate buffer (pH 4.5) for 1 h at 37 °C. After addition of Alkaline phosphatase (CIP, 1 U/µg of RNA, NEB) and CutSmart buffer (final concentration 1 × , NEB), the samples were incubated for another 1 h at 37 °C. Digested RNA samples were diluted in 200 µL and purified over Microcon® – 10 kDa centrifugal filters (Merck). The flow-through was concentrated using a SpeedVac system to the volume of 20 µL for LC-MS analysis.

All samples were measured in technical duplicates. The separation of the digested RNA samples (8 µL injection volume) was performed by an LC system (I-Class, Waters) on a C18 column (Acquity UPLC® BEH C18 1.7 µm, 15 cm, Waters) at 40 °C using a gradient of water (A) and acetonitrile (B), each containing 0.1% (v/v) formic acid^48^. The gradient was 0–6 min, 100% A; 6–7.5 min, 100–99% A; 7.5–9.5 min, 99–94% A; 9.5–15 min, 94% A; 15–25 min, 94–50% A; 25–27 min, 50–20%; 27–29.5 min, 20% A; 29.5–30 min, 20–100% A; 30–40 min, 100% A. The flow rate was 0.05 mL/min. The autosampler cooled the samples to 8 °C. The LC system was coupled on-line to a mass spectrometer (Synapt G2, Waters) to acquire masses of nucleosides by electrospray ionisation. Ions were scanned in a positive polarity mode over full-scan range of *m/z* 100–1200. The source parameters were as follows: capillary voltage, 3 kV; source temperature, 150 °C; sampling cone, 40; extraction cone, 5; desolvation temperature, 450 °C; desolvation gas flow, 600 L/h.

All mass chromatograms were analysed employing the MassLynx V4.1 software. Mixtures of nucleoside standards (m^1^A, m^6^A, Am, A, t^6^A; Jena Bioscience, Sigma-Aldrich, CarboSynth) at three different amounts: 64, 320 and 1600 fmol each were injected on a column to compare the response of each nucleoside under defined ionization conditions. Mixtures were measured in the technical triplicates. For each standard, an extracted ion chromatogram (EIC) was generated using a major fragment observed in its full scan spectrum (fragmentation occurs in the ion source). The chromatographic peaks in EICs were integrated. The standard peak area (area under the curve, AUC) was used to calculate the ionization efficiency ratio of the tested nucleosides in this concentration range (Supplementary Table S3, Figures S1, S2).

The chromatographic peaks of the major fragments in EICs were integrated. The AUC was used to calculate the percentage of the adenosine modifications (Fig. 1A,B).

### Deep sequencing library preparation

Deep sequencing libraries were prepared using a combination of three protocols^12,31,49^.

Chemical fragmentation (metal-ion induced) was used to achieve size distributions of the fragments from 50–200 nt. RNA samples (1–2 µg) were incubated with 100 mM ZnCl_2_ in 100 mM Tris-HCl buffer at pH 7.4 at 75 °C for 1 min. The reaction was terminated by addition of EDTA at a final concentration of 50 mM. Samples were ethanol precipitated and the size of the RNA fragments was verified by HS RNA Screen Tape®.

One half of each sample was incubated with alkaline buffer (50 mM Na_2_CO_2_, 2 mM EDTA, pH 10.4) for 1 h at 60 °C to convert m^1^A into m^6^A via Dimroth rearrangement. Samples were purified by RNA Clean & Concentrator columns (Zymo) and analysed on HS RNA Screen Tape®.

After denaturation of the RNA samples at 90 °C for 30 s, followed by cooling down on ice, dephosphorylation was performed by 0.5 U of FastAP alkaline phosphatase (ThermoFisher Scientific) in dephosphorylation buffer (at a final concentration 100 mM Tris-HCl, pH 7.4, 20 mM MgCl_2_, 0.1 mg/mL BSA and 100 mM 2-mercaptoethanol) for 30 min at 37 °C. The whole procedure was repeated, followed by final heat deactivation of the enzyme at 75 °C for 5 min.

A pre-adenylated adaptor was prepared as described previously^49^ (for the sequence see Supplementary Table S6). Ligation at the 3′-end of RNA was performed at 4 °C for 72 h in dephosphorylation buffer, containing in addition, 15% DMSO, 5 µM adenylated 3′-RNA adaptor, 0.5 U/µL T4 RNA ligase (ThermoFisher Scientific) and 1 U/µL T4 RNA ligase 2 truncated (NEB). The reaction was stopped by heating to 75 °C for 15 min. Unligated 3′-adaptor was removed by 5′-Deadenylase (NEB) and Lambda exonuclease (ThermoFisher Scientific). The mixture was ethanol precipitated.

Reverse transcription with SuperScript III (ThermoFisher Scientific, 10 U/µL) was performed in the First-Strand Buffer, containing RT primer (5 µM), dNTP mix (0.5 mM final conc.), DTT (5 mM), BSA (50 µg/µL) in 30 µL reaction mixture for 1 h at 50 °C. RT primer excess was digested by a combination of Lambda exonuclease, Exonuclease 1 and FastAP alkaline phosphatase (all ThermoFisher Scientific) after reaction. RNA was degraded by NaOH and samples were ethanol precipitated.

Reverse transcription with TGIRT™-III (InGex, 1 µL, 500 nM) was performed in 19 µL of reaction buffer (450 mM NaCl, 5 mM MgCl_2_, 20 mM Tris-HCl, pH 7.5), with DTT (5 mM) and RT primer (5 µM) for 30 min at room temperature. After 30 min dNTPs (1.25 mM each, final volume 20 µL) were added and the reaction was incubated at 60 °C for 50 min. The reaction was stopped by the addition of 1 µL of 5 M NaOH and was incubated for 3 min at 95 °C. Samples were cooled down at room temperature, neutralized with 1 µL of 5 mM HCl and ethanol precipitated.

3′-Tailing of cDNA was performed using 1 U/µL of Deoxynucleotidyl transferase (TdT, ThermoFisher Scientific) in 1x TdT buffer containing 1.25 mM CTP for 30 min at 37 °C with heat deactivation (75 °C, 10 min). Double stranded DNA anchor was prepared as described previously^49^. Ligation of dsDNA adaptor (1.25 µM) was done in 50 mM Tris-HCl buffer (pH 7.4), containing 10 µM ATP and 20 mM MgCl_2_ with T4 DNA ligase (1.5 U/µL) at 4 °C for 72 h. The enzyme was heat deactivated at 65 °C for 10 min. Samples were ethanol precipitated and dissolved in 12 µL of water (molecular biology grade).

PCR amplification was performed with barcoded PCR primers (Supplementary Table S6) in 38 cycles in ThermoPol reaction buffer 1 × (NEB), with 5 µM of each barcoded primer, 0.5 mM dNTPs (each) and 0.25 U of *Taq* DNA Polymerase (NEB) in 20 µL of total reaction mixture. Initial denaturation was performed at 95 °C for 60 s, following by annealing for 60 s at 54 °C, elongation for 60 s at 68 °C and denaturation for 30 s at 95 °C. Final extension was performed at 68 °C for 5 min.

PCR reaction mixture was loaded on 1.3% agarose gel (140 V for 2 h). Fractions between 100–400 nt were cut and DNA was extracted from the gel by NucleoSpin® Gel and PCR Clean-up (Macherey –Nagel).

### Bioinformatics

Libraries were trimmed using cutadapt^50^ and Atropos^51^. Clean sequences longer than 20 bp were mapped to human genome and HIV-1 genome using bwa aligner v0.7.10-r789^52^. All statistics and graphs were generated by custom scripts (github:).

### RNA detection by Northern blotting

Isolated RNA from HIV-1 particles and total RNA isolated from infected MT4 cells were each divided in two parts. One part was chemically fragmented as described for the deep sequencing libraries. Denaturing acrylamide gel (20%, 8 × 10 cm; 1 mm thickness) was prepared from 19:1 acrylamide:bis-acrylamide in 0.5x MOPS buffer (10x MOPS buffer stock containing 0.2 M MOPS pH 7.0, 50 mM NaOAc and 10 mM EDTA) and 7 M urea. The polymerized gel was pre-run at 100 V for 30 min in 0.5x MOPS buffer. 15 µL of the RNA samples (400–600 ng of RNA/sample, containing 7.3% formaldehyde, 50% formamide, 0.5x MOPS buffer and 0.01% bromphenol blue) were denatured for 15 min at 55 °C and loaded into the gel wells. The gel was initially run at 50 V for approximately 15 min to concentrate the samples in wells, and then at 150 V until the bromphenol blue reached 90% of the gel length. The gel was blotted onto a charged nylon membrane (Amersham Hybond-N + ; GE Healthcare) by capillary transfer in 20x SSC buffer (3 M NaCl, 0.3 M tri-sodium citrate, pH adjusted to 7.0) overnight. The membrane was crosslinked twice on a default setting (120 mJ, 30 s) using electronic ultraviolet crosslinker (Ultralum). The crosslinked membrane was hybridized with 10 mL of Church buffer (70 mM NaH_2_PO_4_, 180 mM Na_2_HPO_4_, 7% SDS, 1% BSA, 1 mM EDTA, pH 7.2) at 45 °C for 1 h using a ProBlot hybridization oven (Labnet). Meanwhile, 5 μL of 100 μM probe (Sigma-Aldrich) was end-labelled using 20 U of T4 polynucleotide kinase (NEB), 2 μL of γ-^32^P-ATP (3.3 μM, 10 μCi/μL; Hartmann analytic) in 20 μL of supplemented kinase buffer. The labelling was performed at 37 °C for 30 min. The enzyme was inactivated at 65 °C for 5 min and the probe was purified from unincorporated nucleotides using Micro Bio-Spin P-30 columns (BioRad) according to the manufacturer’s instructions. The probe was added to the membrane in 10 mL of fresh Church buffer and hybridized at 45 °C overnight. The probe was washed twice for 10 min each with low stringency buffer (2x SSC + 0.1% SDS) and once with high stringency buffer (0.1x SSC + 0.1% SDS), all at 45 °C. The membrane was sealed in foil, incubated with phosphor imaging plate (GE healthcare) and read using Typhoon FLA 9500 (GE Healthcare).

## Supplementary information

Supplementary Information

## Data Availability

Data from this manuscript is available upon request from the authors.
