# Supplementary material for: LC/MS analysis and deep sequencing reveal the accurate RNA composition in the HIV-1 virion
Source: Sci Rep. 2019 Jun 18;9:8697. doi: 10.1038/s41598-019-45079-1 (PMC6581912; doi:10.1038/s41598-019-45079-1)
Supplement: Supplementary file 1 — Supplementary Information [file 41598_2019_45079_MOESM1_ESM.pdf]

Supplementary information

LC/MS analysis and deep sequencing reveal the accurate RNA composition in the HIV-1 virion

Anna Šimonová[1,2], Barbora Svojanovská[1,2], Jana Trylčová[1], Martin Hubálek[1], Ondřej Moravčík[3], Martin Zavřel[1], Marcela Pávová[1], Jan Hodek[1], Jan Weber[1], Josef Cvačka[1], Jan Pačes[1,3,4] and Hana Cahová[1,*]

[1] Institute of Organic Chemistry and Biochemistry of the Czech Academy of Sciences, Prague 16610, Czech Republic
[2] First Faculty of Medicine, Charles University, Prague 12108, Czech Republic
[3] Institute of Molecular Genetics of the Czech Academy of Sciences, Prague 14220, Czech Republic
[4] University of Chemistry and Technology, Prague 16628, Czech Republic

* To whom correspondence should be addressed. Tel: +420 220 183 124; Email: cahova@uochb.cas.cz

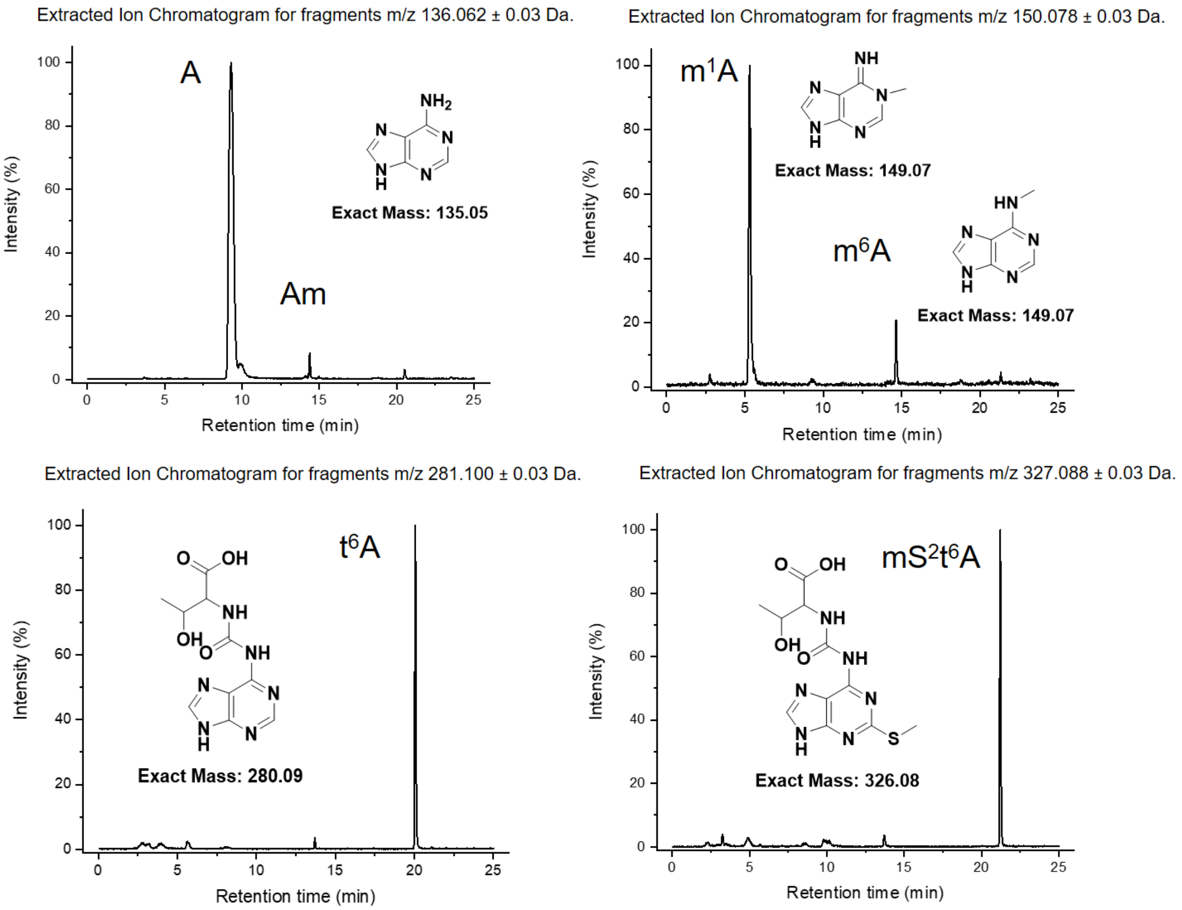

FIgure S1: Extracted Ion Chromatograms (EIC) of detected adenosines in digested RNA from HIV-1 virion.

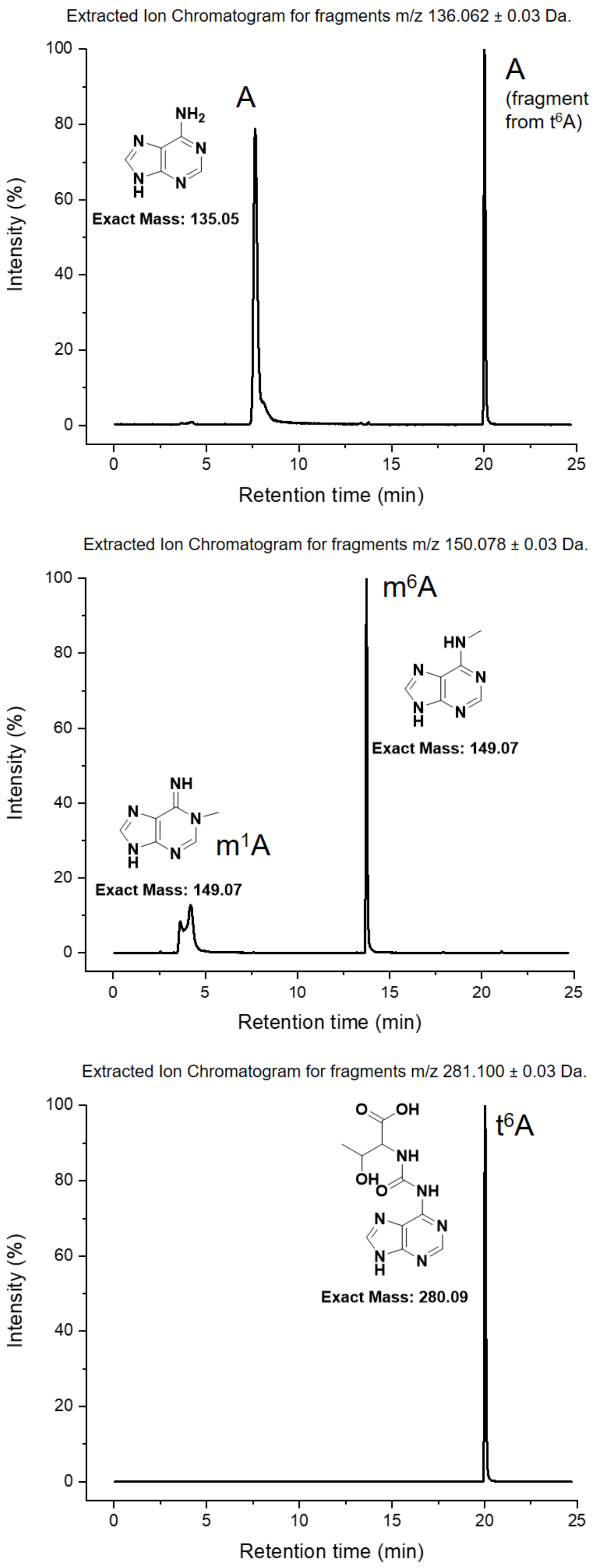

FIgure S2: Extracted Ion Chromatograms (EIC) of standard nucleosides.

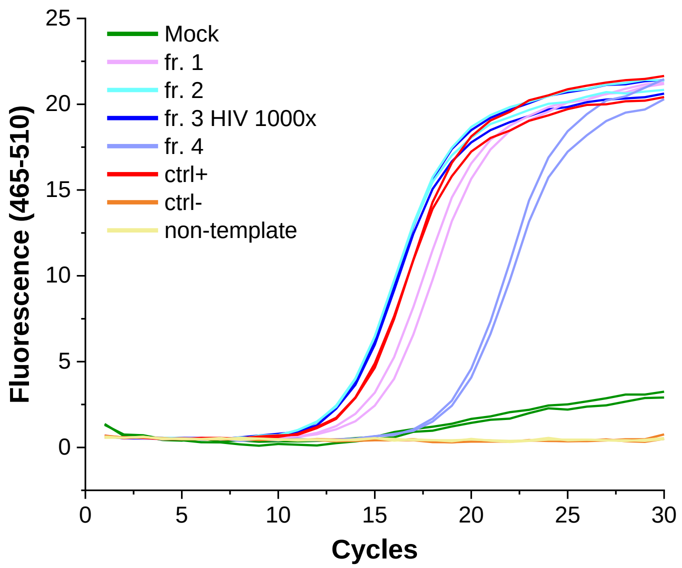

FIgure S3: RT PCR analysis of HIV-1 RNA from various fractions from OptiPrep density gradient purification.

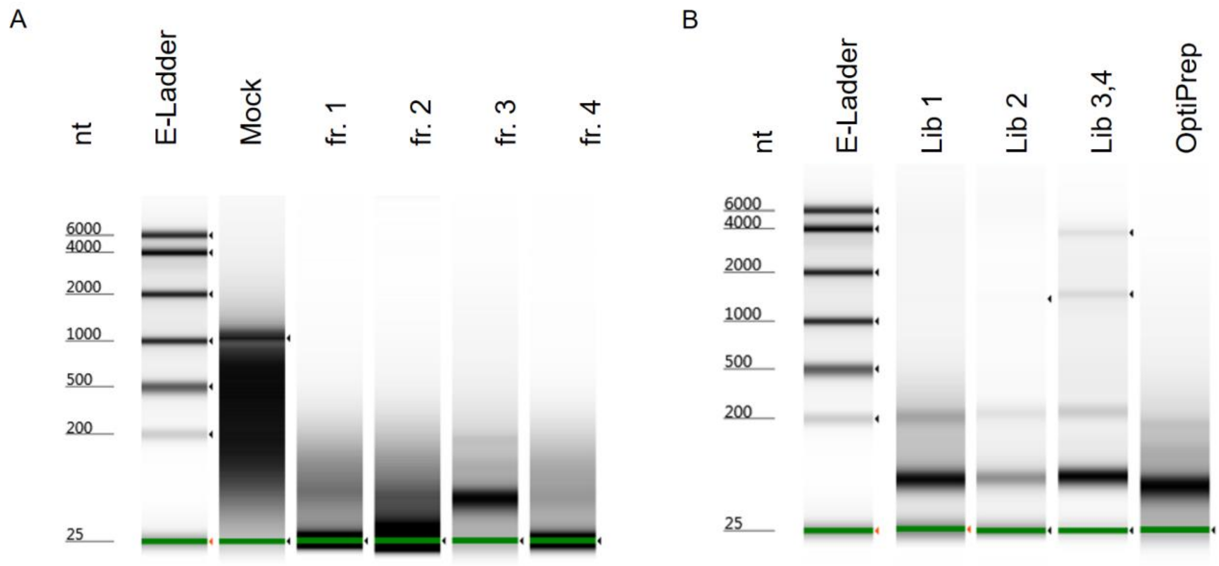

FIgure S4: The results of Tape station analysis. (A) The fractions of HIV-1 RNA after OptiPrep gradient. (B) HIV-1 RNA purified using Sucrose cushion or OptiPrep gradient.

Quality of mapping reads to tRNA-Lys

A: Multiple alignment of three human Lys tRNAs

```
hs-tRNA-Lys-TTT-3-1 GCCCGGATAGCTCAGTCGGTAGAGCATCAGACTTTTAATCTGAGGGTCCAGGGTTCAAGTCCCTGTTCGGGCG
hs-tRNA-Lys-CTT-1-1 GCCCGGCTAGCTCAGTCGGTAGAGCATGGGACTCTTAATCCCAGGGTCGTGGGTTCGAGCCCCACGTTGGGCG
hs-tRNA-Lys-CTT-2-1 GCCCGGCTAGCTCAGTCGGTAGAGCATGAGACTCTTAATCTCAGGGTCGTGGGTTCGAGCCCCACGTTGGGCG
                   ****** *******************  **** ******  ******  ****** ** ***   * *****
```

B: Distinguishing positions for three human Lys tRNAs and percentage of reads for each base on those positions

```
hstRNA-Lys-TTT-3-1
     GCCCGG A     TAGCTCAGTCGGTAGAGCAT C     A     GACT T     TTAATC T     G     AGGGTC C     A     GGGTTC A     AG T     CCC T     G     T     T C     GGGCG
A:          99.0                       0.0   99.8       1.7          0.0   0.0          0.0   100          100      0.0       0.0   0.0   0.0     0.0
C:          0.9                        99.5  0.0        0.8          0.0   0.0          100   0.0          0.0      0.0       0.0   0.0   0.0     100
G:          0.0                        0.5   0.1        0.4          0.0   100          0.0   0.0          0.0      0.0       0.0   100   0.0     0.0
T:          0.0                        0.0   0.0        97.0         100   0.0          0.0   0.0          0.0      100       100   0.0   100     0.0
hstRNA-Lys-CTT-1-1
     GCCCGG C     TAGCTCAGTCGGTAGAGCAT G     G     GACT C     TTAATC C     C     AGGGTC G     T     GGGTTC G     AG C     CCC A     C     G     T T     GGGCG
A:          0.6                        0.1   0.7        0.1          0.0   0.1          0.0   0.0          0.0      0.1       99.8  0.0   0.0     0.0
C:          99.1                       0.0   0.0        99.8         100   99.9         0.0   0.1          0.0      98.4      0.1   99.9  0.0     0.3
G:          0.1                        99.8  99.3       0.0          0.0   0.0          99.9  0.2          99.9     1.5       0.1   0.0   99.9    0.0
T:          0.2                        0.1   0.1        0.1          0.0   0.0          0.1   99.7         0.1      0.1       0.0   0.0   0.0     99.7
hstRNA-Lys-CTT-2-1
     GCCCGG C     TAGCTCAGTCGGTAGAGCAT G     A     GACT C     TTAATC T     C     AGGGTC G     T     GGGTTC G     AG C     CCC A     C     G     T T     GGGCG
A:          0.4                        0.0   100        0.1          0.0   0.1          0.0   0.0          0.0      0.1       99.8  0.0   0.0     0.0
C:          99.4                       0.1   0.0        99.8         0.4   99.9         0.0   0.1          0.1      98.7      0.1   99.9  0.0     0.3
G:          0.1                        99.9  0.0        0.0          0.0   0.0          99.9  0.2          99.8     1.1       0.1   0.0   99.9    0.0
T:          0.1                        0.1   0.0        0.1          99.5  0.0          0.1   99.7         0.1      0.1       0.0   0.0   0.0     99.7
```

FIgure S5: The quality of mapping reads to tRNA^{Lys}

A

Type of RNA	Copies of RNA based on literature	Length (nt)	Number of A per sequence
HIV-1 gRNA	2	9181	3272
7 SL RNA	14	299	56
tRNA $^{lys}_{TTT-3.1}$	8	76	15
tRNA $^{lys}_{CTT-2.1}$ +tRNA $^{lys}_{CTT-1.1}$	12	76	15
Other tRNA	50	Average 76	15

B

Nucleoside	%
A	89.3 ± 2.0
m^1A	4.1 ± 1.0
m^6A	1.1 ± 0.2
Am	0.9 ± 1.0
t^6A	3.2 ± 0.8
mS^2t^6A	1.5 ± 0.9*

C

$x = m^1A$
$n = \sum A$

4.1% n= 0.041 n = x
G = 2 gRNA HIV= 6544 A
S = 14 7SL RNA = 784 A
1 tRNA = T= 15 A

1/15 T= x
0.067 T= 0.041 n
n = 1.634 T
n = T+784+6544
1.634 T=T+784+6544
0.634 T=7328
T=11558 /15
tRNA= 770

*Figure S 6: The calculations used for the quantification of tRNA molecules per HIV-1 virion. A) Table of length and number of A per sequence of HIV-1 packageome. B) The percentage of LC/MS detected nucleosides from HIV-1 packageome RNA. * The ionization property of mS^2t^6A was estimated based on chemical similarity with t^6A. C) The equations used for the quantification of tRNAs per HIV-1 virion.*

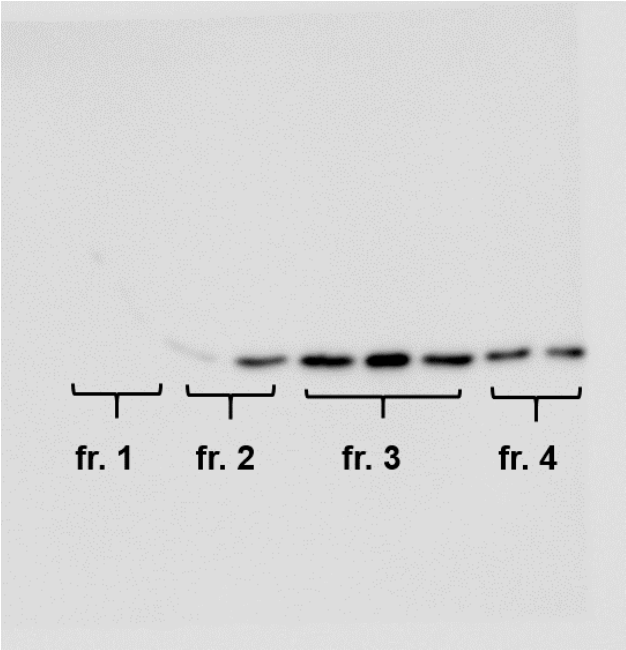

FIgure S7: The Western blot analysis of HIV-1 virions purified by OptiPrep gradient.

Table S1: Calculation of theoretical m^{1}A position in HIV-1 packageome based on data from literature

Type of RNA	Copies of RNA based on literature	Number of A per sequence
HIV-1 gRNA	2	3272
7 SL RNA	14	56
tRNA $^{lys\ TTT\text{-}3.1}$	8	15
tRNA $^{lys\ CTT\text{-}2.1}$ +tRNA $^{lys\ CTT\text{-}1.1}$	12	15
Other tRNA	50	15
Total A ~ 8378		
(4.1 %) m^{1}A ~ 343		

Table S2: The list of known adenosine modifications screened in analysed digested RNA from HIV-1 virion (n.d. = not detected, detected = only traces).

Name	Formula	Short Name	Presence in HIV virion sample
1,2′-O-dimethyladenosine	$C_{12}O_4N_5H_{17}$	m1Am	n.d.
1,2′-O-dimethylinosine	$C_{12}O_5N_4H_{16}$	m1Im	n.d.
1-methyladenosine	$C_{11}O_4N_5H_{15}$	m1A	confirmed
1-methylinosine	$C_{11}O_5N_4H_{14}$	m1I	n.d.
2,8-dimethyladenosine	$C_{12}H_{17}N_5O_4$	m2,8A	n.d.
2- methylthiomethylenethio-N6-isopentenyl-adenosine	$C_{17}H_{25}N_5O_4S_2$	msms2i6A	n.d.
2-methyladenosine	$C_{11}O_4N_5H_{15}$	m2A	n.d.
2-methylthio cyclic N6-threonylcarbamoyladenosine	$C_{17}H_{21}N_5O_7S$	ms2ct6A	n.d.
2-methylthio-N6-(cis-hydroxyisopentenyl) adenosine	$C_{16}O_5N_5H_{23}S_1$	ms2io6A	n.d.
2-methylthio-N6-hydroxynorvalylcarbamoyladenosine	$C_{17}O_8N_6H_{24}S_1$	ms2hn6A	n.d.
2-methylthio-N6-isopentenyladenosine	$C_{16}O_4N_5H_{23}S_1$	ms2i6A	detected
2-methylthio-N6-methyladenosine	$C_{12}O_4N_5H_{17}S_1$	ms2m6A	n.d.
2-methylthio-N6-threonylcarbamoyladenosine	$C_{16}O_8N_6H_{22}S_1$	ms2t6A	detected
2′-O-methyladenosine	$C_{11}O_4N_5H_{15}$	Am	confirmed
2′-O-methylinosine	$C_{11}O_5N_4H_{14}$	Im	n.d.
8-methyladenosine	$C_{11}O_4N_5H_{15}$	m8A	n.d.
N6,2′-O-dimethyladenosine	$C_{12}O_4N_5H_{17}$	m6Am	n.d.
N6,N6,2′-O-trimethyladenosine	$C_{13}O_4N_5H_{19}$	m6,6Am	n.d.
N6,N6-dimethyladenosine	$C_{12}O_4N_5H_{17}$	m6,6A	n.d.
N6-(cis-hydroxyisopentenyl)adenosine	$C_{15}O_5N_5H_{21}$	io6A	detected
N6-acetyladenosine	$C_{12}O_5N_5H_{15}$	ac6A	n.d.
N6-formyladenosine	$C_{11}H_{13}N_5O_5$	f6A	n.d.
N6-glycinylcarbamoyladenosine	$C_{13}O_7N_6H_{16}$	g6A	n.d.
N6-hydroxymethyladenosine	$C_{11}H_{15}N_5O_5$	hm6A	n.d.
N6-hydroxynorvalylcarbamoyladenosine	$C_{16}O_8N_6H_{22}$	hn6A	n.d.
N6-isopentenyladenosine	$C_{15}O_4N_5H_{21}$	i6A	detected
N6-methyl-N6-threonylcarbamoyladenosine	$C_{16}O_8N_6H_{22}$	m6t6A	n.d.

N6-methyladenosine	$C_{11}O_4N_5H_{15}$	m6A	confirmed
N6-threonylcarbamoyladenosine	$C_{15}O_8N_6H_{20}$	t6A	confirmed
cyclic N6-threonylcarbamoyladenosine	$C_{15}O_7N_6H_{18}$	ct6A	n.d.
hydroxy-N6-threonylcarbamoyladenosine	$C_{15}H_{20}N_6O_9$	ht6A	n.d.
inosine	$C_{10}O_5N_4H_{12}$	I	n.d.

Table S3: The area under the curve of the tested nucleosides. Ionization ratios were used for the calculation of percent of detected adenosines. The ionization property of mS^2t^6A was estimated based on structural similarity with t^6A.

nucleoside	Amount (fmol)	AUC 1st meas.	ratio	AUC 2nd meas.	ratio	AUC 3rd meas.	ratio	AUC average	STDEV	AUC ratio average	STDEV
A	64.0	124.5	0.79	149.5	0.77	118.9	0.56	131.0	16.3	0.5	0.18
A	320.0	335.5	0.45	366.4	0.45	354.3	0.41	352.1	15.6		
A	1600.0	1149.9	0.33	1170.9	0.34	1222.5	0.34	1181.1	37.4		
m^1A	64.0	176.2	1.12	163.2	0.84	112.7	0.53	150.7	33.5	0.6	0.23
m^1A	320.0	409.9	0.54	449.0	0.55	424.4	0.50	427.8	19.8		
m^1A	1600.0	1464.1	0.43	1561.1	0.45	1794.3	0.50	1606.5	169.7		
m^6A	64.0	156.7	1.00	194.8	1.00	212.6	1.00	188.0	28.5	1.0	
m^6A	320.0	753.2	1.00	816.8	1.00	855.1	1.00	808.3	51.4		
m^6A	1600.0	3444.7	1.00	3452.4	1.00	3565.2	1.00	3487.4	67.4		
t^6A	64.0	143.9	0.92	141.3	0.73	166.4	0.78	150.5	13.8	0.9	0.09
t^6A	320.0	649.5	0.86	699.8	0.86	711.7	0.83	687.0	33.0		
t^6A	1600.0	3235.6	0.94	3396.3	0.98	3451.1	0.97	3361.0	112.0		
Am	64.0	133.3	0.93	158.1	0.81	217.8	1.02	169.7	43.4	0.6	0.22
Am	320.0	391.1	0.52	455.3	0.56	403.9	0.47	416.8	34.0		
Am	1600.0	1699.2	0.49	1669.3	0.48	1826.0	0.51	1731.5	83.2		

Table S4: The LC/MS analysis of digested RNA isolated from mock medium from the uninfected MT4 cells.

Nucleoside	%
A	99.0
m^1A	0.1
m^6A	0.0
t^6A	0.5
Am, mS^2t^6A	0.6

Table S5: Bioinformatics analysis. Reads of the most abundant RNAs – first 20 tRNAs and other non tRNA sequences detected in HIV-1 virions deep sequencing analysis.

data	libAS2-m1AHIV-sb-Hv3.sam	libAS2-m6AHIV-sb-Hv3.sam	libAS3-m1AHIV-sb-Hv3.sam	libAS3-m6AHIV-sb-Hv3.sam	libAS4-m1AHIV-sb-Hv3.sam	libAS4-m6AHIV-sb-Hv3.sam
7SL	74.98	56.88	43.15	33.44	90.61	31.29
HIVNL43	2.82	1.30	1.10	1.05	5.13	1.80
ALU	0.72	0.67	0.61	0.53	2.82	0.80
RNU1-1	0.11	0.06	2.96	0.67	1.39	0.54
RNU6-1	0.79	0.35	0.70	0.55	1.73	0.79
VTRNA1-1	0.59	0.73	0.83	0.55	1.31	0.33
SNAR-A1	0.05	0.06	0.04	0.03	0.15	0.03
L1	0.00	0.00	0.00	0.00	0.14	0.03
hstRNA-Lys-TTT-3-1	1312.60	157.12	914.85	278.86	440.15	214.88
hstRNA-Lys-CTT-2-1	330.63	62.05	1341.64	85.73	572.93	154.70
hstRNA-Lys-CTT-1-1	274.87	52.61	874.39	72.15	382.84	96.52
hstRNA-Asp-GTC-2-1	28.62	16.01	354.96	22.65	239.89	47.31

hstRNA-Asn-GTT-2-1	54.97	26.35	195.30	23.65	44.53	20.55
hstRNA-Lys-CTT-4-1	68.94	38.16	125.66	38.84	41.88	29.49
hstRNA-Glu-CTC-1-1	24.08	13.57	64.41	16.33	86.05	34.78
hstRNA-Lys-CTT-3-1	46.89	36.18	69.83	34.12	27.88	22.23
hstRNA-Lys-TTT-4-1	73.06	7.50	86.49	14.58	38.05	13.83
hstRNA-Lys-TTT-2-1	66.38	6.75	82.14	13.97	35.56	13.47
hstRNA-Gly-GCC-2-1	15.51	12.55	108.18	19.98	36.63	15.00
hstRNA-Asn-GTT-3-1	25.52	20.51	56.48	15.35	9.97	6.24
hstRNA-His-GTG-1-1	9.97	3.62	32.92	5.71	21.01	7.94
hstRNA-Gly-TCC-2-1	7.61	1.86	34.10	4.24	18.67	8.55
hstRNA-Met-CAT-6-1	10.48	5.97	23.73	7.43	12.56	7.67
hstRNA-Lys-TTT-5-1	16.69	4.53	18.03	5.99	9.82	1.95
hstRNA-Asp-GTC-3-1	5.10	8.72	13.32	5.85	13.11	6.63
hstRNA-Gly-GCC-1-1	5.35	4.16	19.21	4.77	8.57	3.75
hstRNA-Asp-GTC-1-1	1.77	1.59	24.16	1.90	12.02	3.02
hstRNA-Met-CAT-1-1	7.86	1.30	15.66	3.79	10.15	5.65
hstRNA-Glu-TTC-2-1	3.01	1.66	19.20	2.86	11.98	3.11

Table S6: Sequences.

3'-adaptor	5´p-CNNNNNNAGATCGGAAGAGCACACGTCTG-(C3)
RT primer	5´CAGACGTGTGCTCTTCCGAT
cDNA anchor sense oligonucleotide	5´p-CAGATCGGAAGAGCGTCGTGT-(C3)
cDNA anchor antisense oligonucleotide	5´ACACGACGCTCTTCCGATCTGGG
Probe 3' Lys TTT	5´TGGCGCCCGAACAGGGAC
Probe 5' Lys	5´ACCGACTGAGCTATCCGGGC
Probe 3' Lys CTT	5´TGGCGCCCAACGTGGGGC
76mer sense	TAATACGACTCACTATTAGGGCCCGGATAGCTCAGTCGGTAGAGCATCAGACTTTTAATCTGAGGGTCCAGGGTTCAAGTCCCTGTTCGGGCGCCA
76mer antisense	TGGCGCCCGAACAGGGACTTGAACCCTGGACCCTCAGATTAAAAGTCTGATGCTCTACCGACTGAGCTATCCGGGCCC**T**AATAGTGAGTCGTATTA
39mer sense	TAATACGACTCACTATT**A**GGGATCTGAGGGTCCAGGGTTCAAGTCCCTGTTCGGGCGCCA
39mer antisense	TGGCGCCCGAACAGGGACTTGAACCCTGGACCCTCAGATCCC**T**AATAGTGAGTCGTATTA
18mer sense	TAATACGACTCACTATT**A**GGGTCCCTGTTCGGGCGCCA
18mer antisense	TGGCGCCCGAACAGGGACCC**T**AATAGTGAGTCGTATTA
NGS primers	
ForPrimer MID1s	ACGAGTGCGTACACGACGCTCTTCCGATCT
ForPrimer MID5s	ATCAGACACGACACGACGCTCTTCCGATCT
ForPrimer MID11s	TGATACGTCTACACGACGCTCTTCCGATCT
ForPrimer MID12s	TACTGAGCTAACACGACGCTCTTCCGATCT
ForPrimer MID27s	ACGCGAGTATACACGACGCTCTTCCGATCT
ForPrimer MID29s	ACTGTACAGTACACGACGCTCTTCCGATCT
ForPrimer MID34s	CACGCTACGTACACGACGCTCTTCCGATCT
ForPrimer MID35s	CAGTAGACGTACACGACGCTCTTCCGATCT
ForPrimer MID36s	CGACGTGACTACACGACGCTCTTCCGATCT
ForPrimer MID37s	TACACACACTACACGACGCTCTTCCGATCT
ForPrimer MID39s	TACAGATCGTACACGACGCTCTTCCGATCT
ForPrimer MID40s	TACGCTGTCTACACGACGCTCTTCCGATCT
RevPrimer MID3s	AGACGCACTCCAGACGTGTGCTCTTCCGATCT
RevPrimer MID7s	CGTGTCTCTACAGACGTGTGCTCTTCCGATCT
RevPrimer MID14s	CGAGAGATACCAGACGTGTGCTCTTCCGATCT
RevPrimer MID18s	TCTACGTAGCCAGACGTGTGCTCTTCCGATCT
RevPrimer MID32s	AGTACGCTATCAGACGTGTGCTCTTCCGATCT
RevPrimer MID33s	ATAGAGTACTCAGACGTGTGCTCTTCCGATCT
RevPrimer MID50s	ACTAGCAGTACAGACGTGTGCTCTTCCGATCT
RevPrimer MID55s	CGATCGTATACAGACGTGTGCTCTTCCGATCT
RevPrimer MID61s	CTATAGCGTACAGACGTGTGCTCTTCCGATCT
RevPrimer MID84s	AGTGACACACCAGACGTGTGCTCTTCCGATCT

RevPrimer MID91s	CATACTCTACCAGACGTGTGCTCTTCCGATCT
RevPrimer MID93s	CGAGACGCGCCAGACGTGTGCTCTTCCGATCT

Table S7: Bioinformatic analysis.

tRNA$^{Lys\ TTT-3.1}$

- Stops (coverage drops)
- Errors (base mismatch)
- Pattern: "gttc.A..c"

	Lib 1 m^{1}A	Lib 2 m^{1}A	Lib 3 m^{1}A	Lib 4 m^{1}A-T	Lib 1 m^{6}A	Lib 2 m^{6}A	Lib 3 m^{6}A	Lib 4 m^{6}A-T	p-value
36 (T)	32%	35%	49%	9%	42%	64%	51%	56%	0.06
	5% (A: 4%)	8% (A: 6%)	13% (A: 9%)	2% (A: 2%)	2% (A: 2%)	4% (A: 3%)	9% (A: 9%)	4% (A: 3%)	0.48
	(14636)	(10711)	(2444)	(92)	(1120)	(341)	(1185)	(96)	
37 (A)	94%	96%	98%	91%	94%	97%	98%	92%	0.76
	1% (G: 0%)	1% (G: 0%)	1% (C: 0%)	45% (T: 44%)	0% (G: 0%)	1% (T: 0%)	1% (G: 0%)	1% (T: 0%)	0.34
	(20870)	(14963)	(3937)	(98)	(1773)	(685)	(2102)	(195)	
43 (A)	1%	0%	0%	1%	10%	10%	4%	4%	0.01
	1% (G: 1%)	1% (G: 1%)	0% (G: 0%)	0% (G: 0%)	3% (G: 3%)	5% (G: 5%)	1% (G: 1%)	1% (G: 1%)	0.09
	(375673)	(494680)	(213871)	(1379)	(45869)	(36926)	(139728)	(3001)	
58 (A)	0%	0%	0%	0%	0%	0%	0%	0%	----
	77% (G: 65%)	79% (G: 61%)	74% (G: 55%)	77% (T: 57%)	35% (G: 30%)	29% (G: 23%)	38% (G: 30%)	42% (G: 22%)	0.00
	(379511)	(498535)	(215046)	(1396)	(51427)	(41189)	(146293)	(3129)	

Overall statistics

	Lib 1 m^{1}A	Lib 2 m^{1}A	Lib 3 m^{1}A	Lib 4 m^{1}A-T	Lib 1 m^{6}A	Lib 2 m^{6}A	Lib 3 m^{6}A	Lib 4 m^{6}A-T
Total reads	384617	508177	219874	1414	52843	42122	147578	3214
Average coverage	191960 (σ=182085)	251919 (σ=240044)	108238 (σ=103863)	711 (σ=643)	25357 (σ=24145)	20191 (σ=19582)	72024 (σ=71271)	1556 (σ=1464)
Average errors	4182 (σ=34276)	5624 (σ=46061)	2224 (σ=18507)	15 (σ=125)	288 (σ=2153)	212 (σ=1422)	830 (σ=6540)	19 (σ=155)
Total errors	2.18%	2.23%	2.05%	2.22%	1.14%	1.05%	1.15%	1.26%
Total "A" errors	0.02%	0.01%	0.01%	0.01%	0.01%	0.02%	0.01%	0.01%

tRNA$^{Lys\ TTT-3.1}$ (Lib 1 m^{1}A)

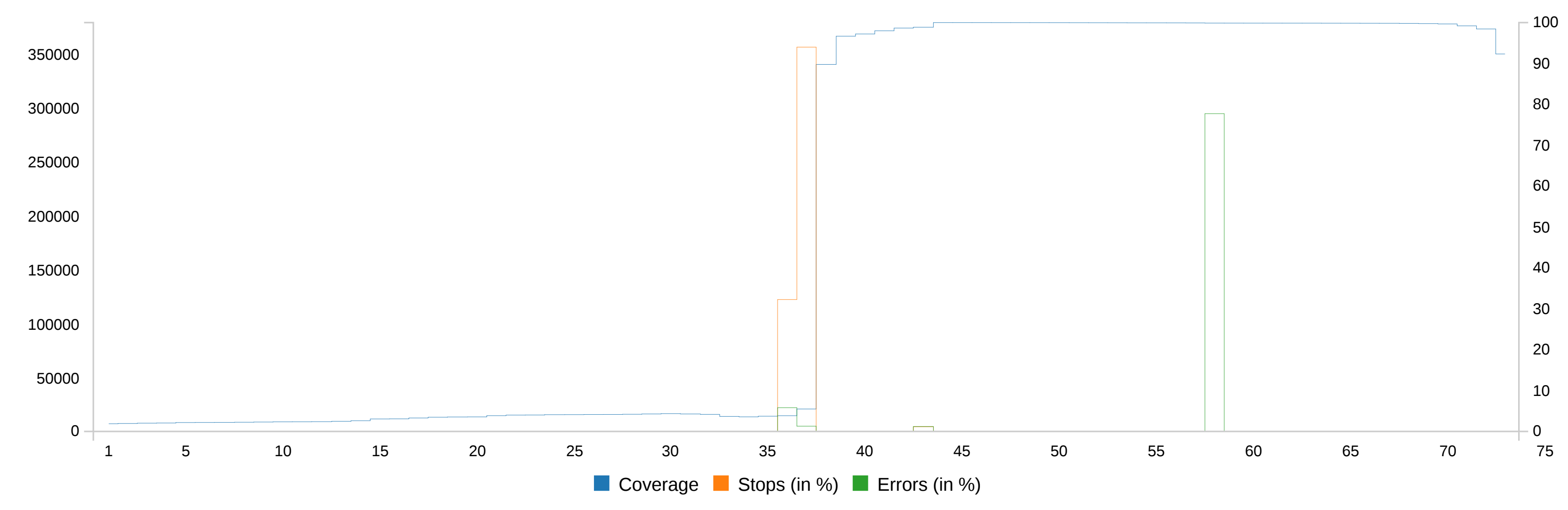

tRNA$^{Lys\ TTT-3.1}$ (Lib 2 m^{1}A)

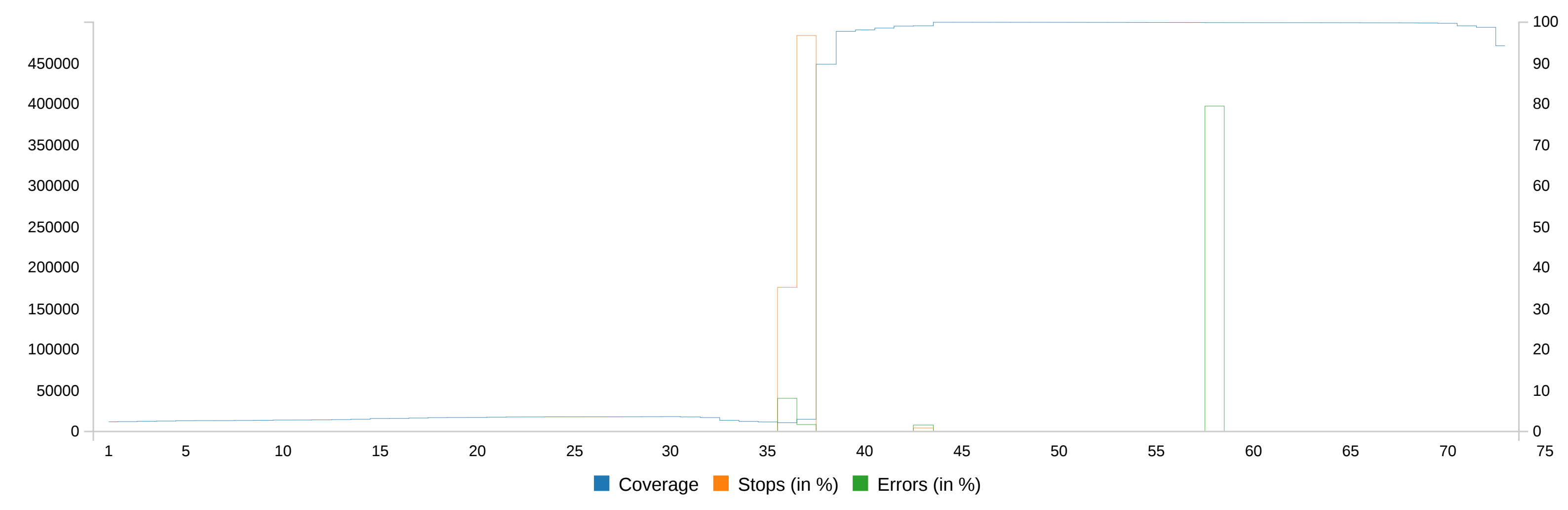

tRNA$^{Lys\ TTT-3.1}$ (Lib 3 m^{1}A)

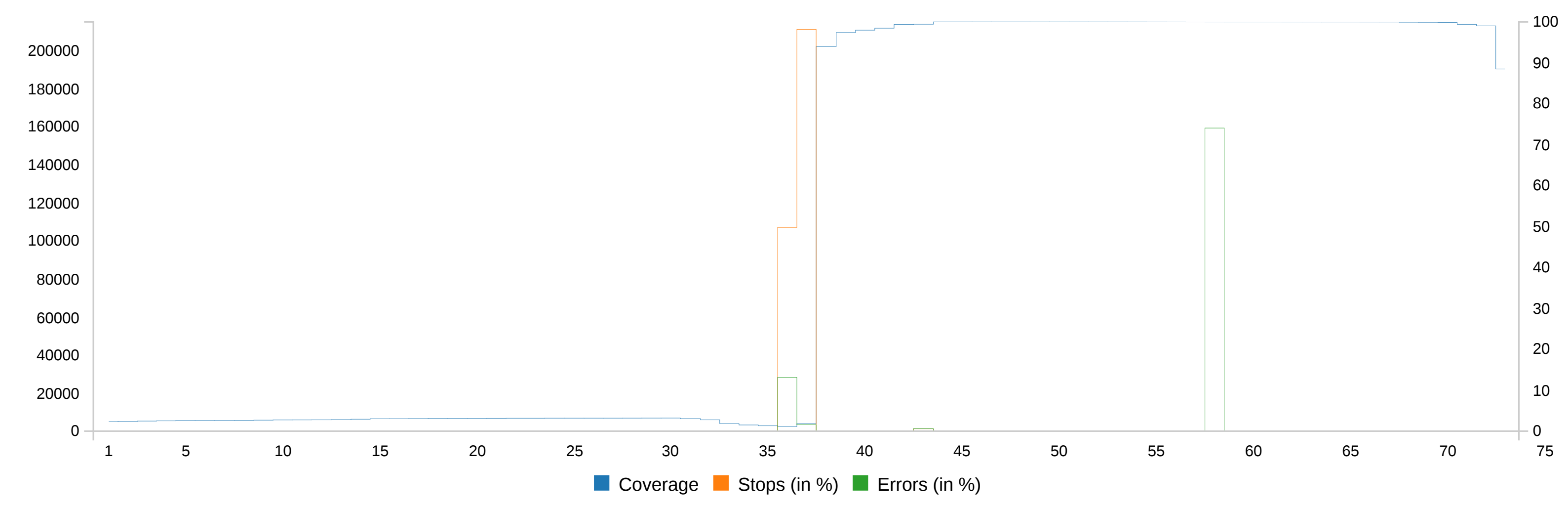

tRNA$^{Lys\ TTT-3.1}$ (Lib 4 m^{1}A-T)

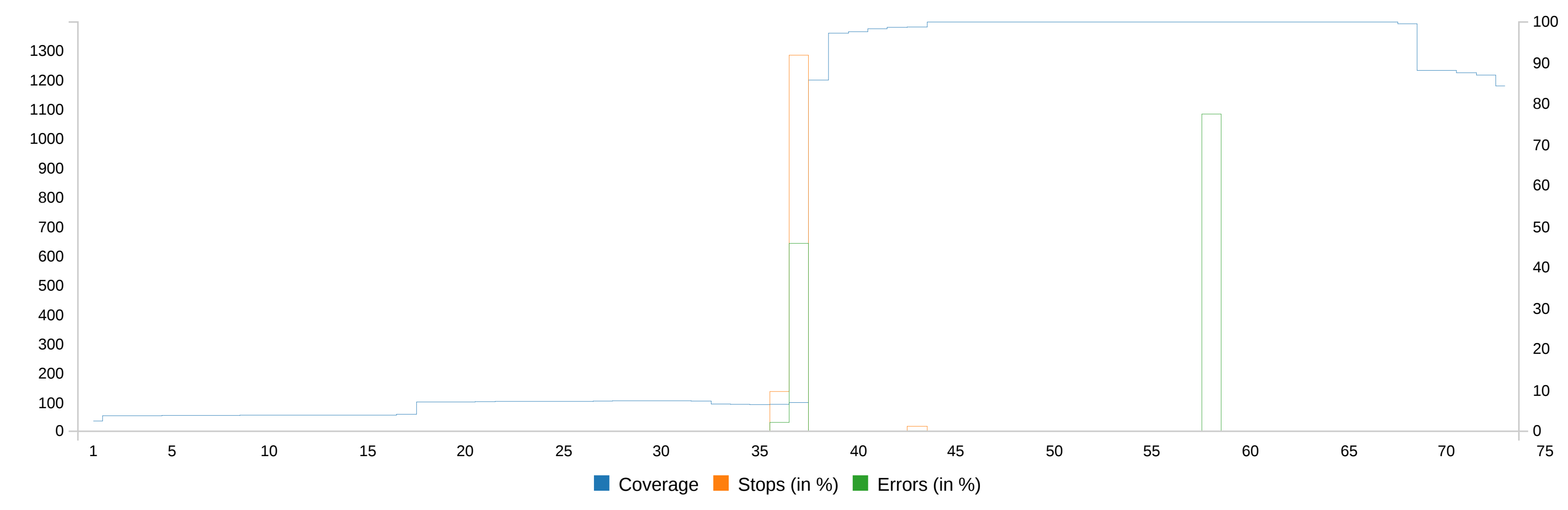

tRNA$^{Lys\ TTT-3.1}$ (Lib 1 m^{6}A)

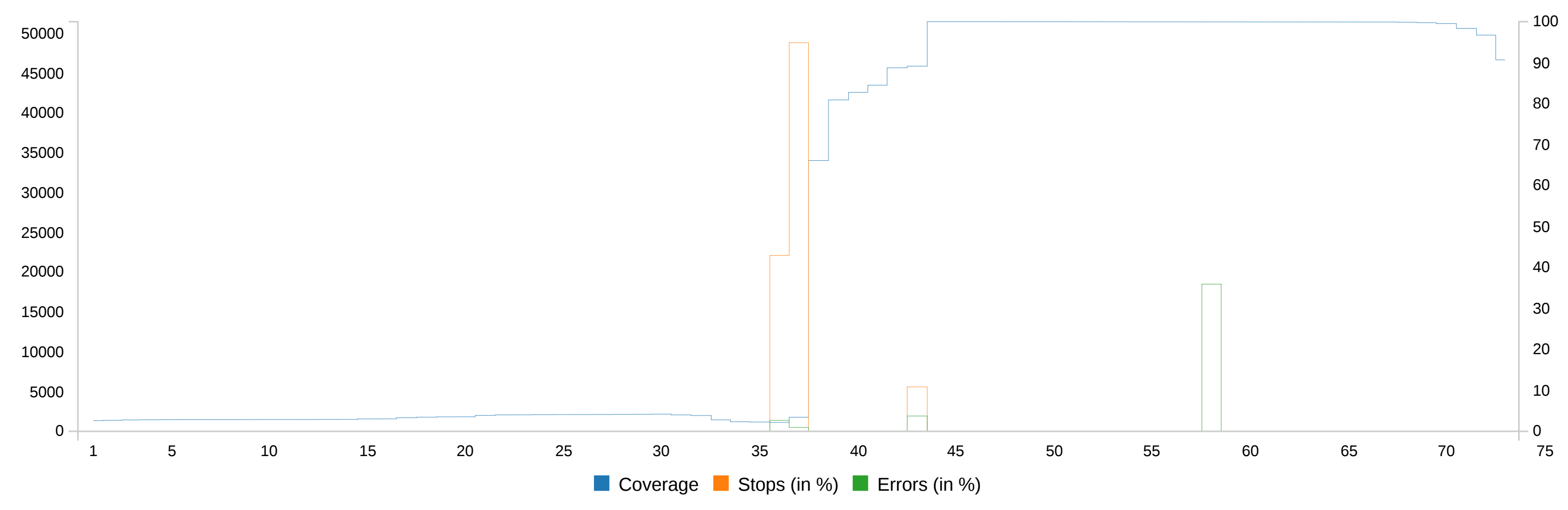

tRNA$^{Lys\ TTT-3.1}$ (Lib 2 m^{6}A)

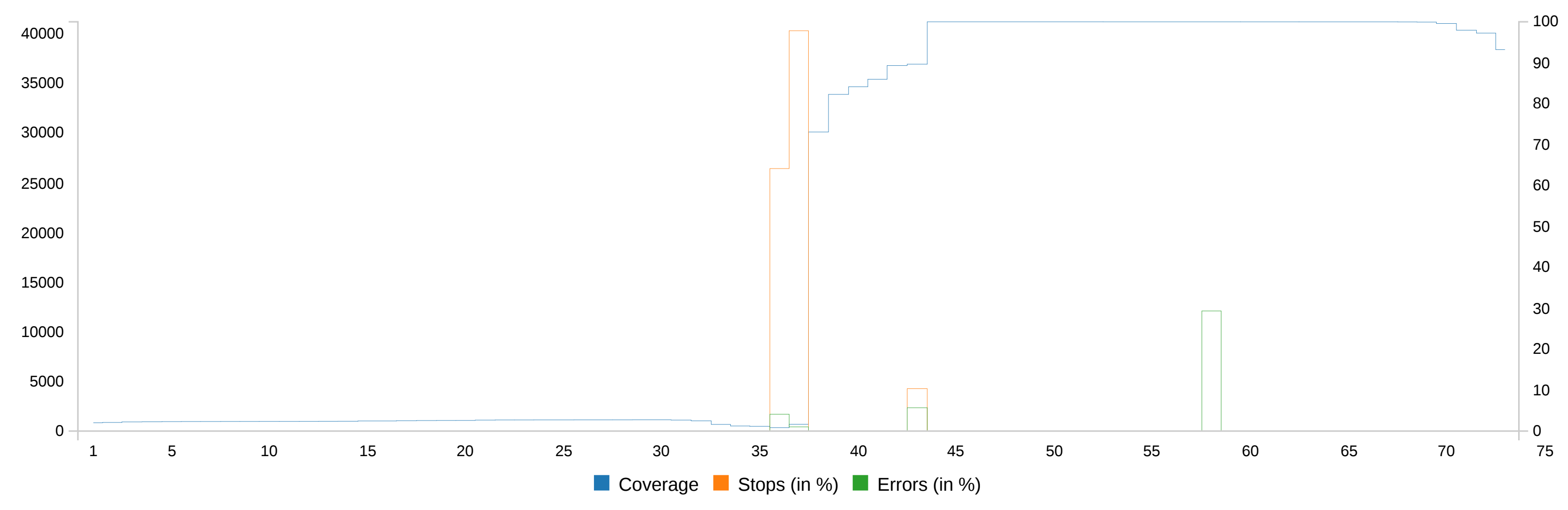

tRNA$^{Lys\ TTT-3.1}$ (Lib 3 m^{6}A)

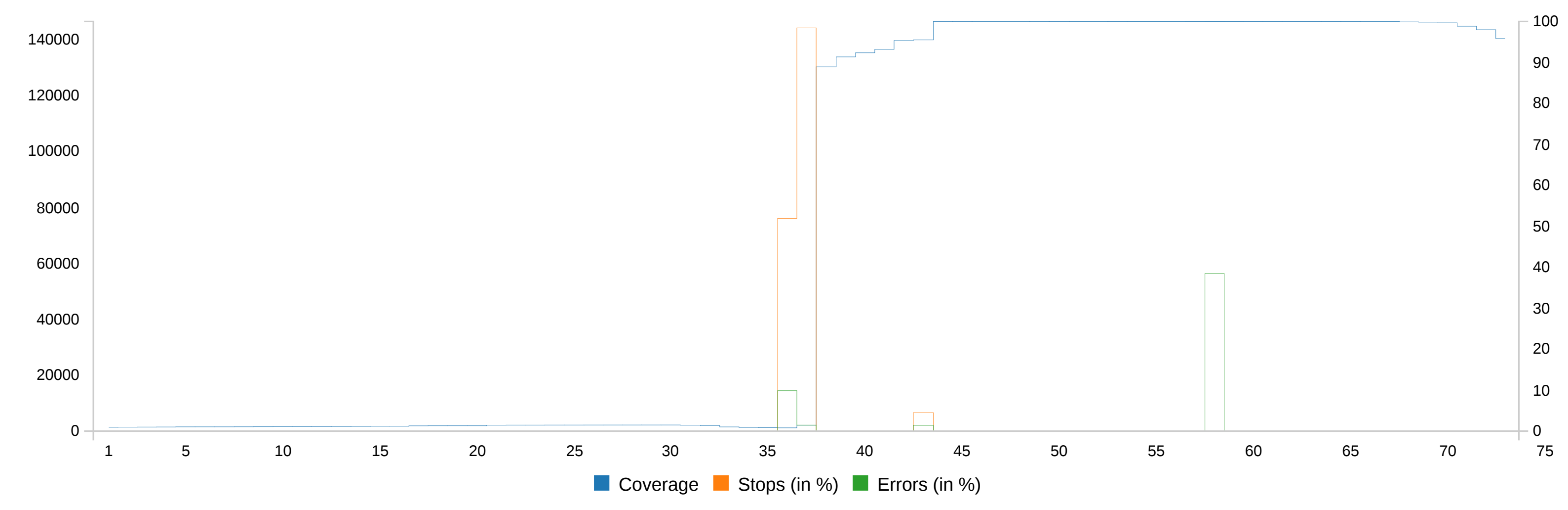

tRNA$^{Lys\ TTT-3.1}$ (Lib 4 m^{6}A-T)

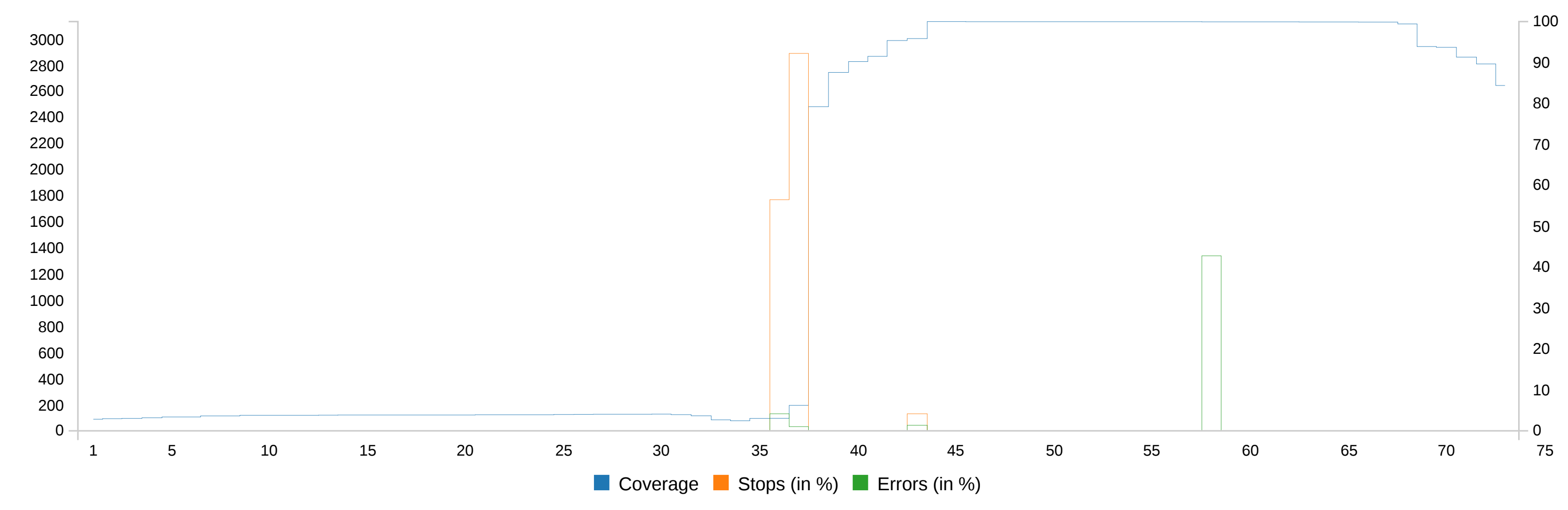

tRNA^{Lys} CTT-2.1

- Stops (coverage drops)
- Errors (base mismatch)
- Pattern: "gttc.A..c"

	Lib 1 m^{1}A	Lib 2 m^{1}A	Lib 3 m^{1}A	Lib 4 m^{1}A-T	Lib 1 m^{6}A	Lib 2 m^{6}A	Lib 3 m^{6}A	Lib 4 m^{6}A-T	p-value
16 (T)	7%	4%	3%	13%	27%	23%	20%	14%	0.01
	0% (C: 0%)	0% (G: 0%)	0% (G: 0%)	0% (C: 0%)	0% (C: 0%)	0% (C: 0%)	0% (C: 0%)	4% (C: 4%)	0.31
	(56915)	(554025)	(217581)	(3519)	(3204)	(3529)	(49401)	(2077)	
17 (C)	6%	4%	3%	1%	12%	10%	6%	4%	0.07
	0% (A: 0%)	0% (T: 0%)	0% (T: 0%)	0% (G: 0%)	0% (T: 0%)	0% (T: 0%)	0% (A: 0%)	0% (A: 0%)	0.33
	(61212)	(582644)	(226362)	(4045)	(4426)	(4599)	(62241)	(2425)	
20 (T)	7%	4%	5%	1%	22%	17%	14%	8%	0.02
	0% (A: 0%)	0% (G: 0%)	0% (G: 0%)	0% (C: 0%)	0% (C: 0%)	0% (C: 0%)	1% (C: 1%)	10% (C: 10%)	0.27
	(68125)	(623066)	(237720)	(4268)	(5297)	(5396)	(68023)	(2666)	
43 (A)	2%	0%	0%	0%	40%	20%	10%	3%	0.07
	0% (G: 0%)	0% (G: 0%)	0% (G: 0%)	0% (G: 0%)	0% (G: 0%)	0% (G: 0%)	0% (G: 0%)	0% (G: 0%)	0.29
	(89801)	(729562)	(273583)	(4959)	(11699)	(9875)	(91648)	(3540)	
58 (A)	0%	0%	0%	0%	0%	0%	0%	0%	----
	63% (T: 32%)	74% (T: 48%)	60% (T: 40%)	92% (T: 65%)	9% (T: 4%)	28% (T: 18%)	29% (T: 17%)	39% (T: 24%)	0.00
	(86069)	(686250)	(255304)	(4722)	(19108)	(11904)	(96991)	(3357)	

Overall statistics

	Lib 1 m^{1}A	Lib 2 m^{1}A	Lib 3 m^{1}A	Lib 4 m^{1}A-T	Lib 1 m^{6}A	Lib 2 m^{6}A	Lib 3 m^{6}A	Lib 4 m^{6}A-T
Total reads	96861	745214	286502	5064	20844	12924	106286	3815
Average coverage	74059 (σ=19605)	633373 (σ=115565)	236896 (σ=51410)	4210 (σ=987)	11416 (σ=6780)	8260 (σ=3633)	79765 (σ=23559)	2880 (σ=863)
Average errors	875 (σ=6334)	8513 (σ=59645)	2449 (σ=18010)	66 (σ=505)	33 (σ=202)	60 (σ=397)	506 (σ=3304)	29 (σ=155)
Total errors	1.18%	1.34%	1.03%	1.59%	0.29%	0.73%	0.64%	1.03%
Total "A" errors	0.06%	0.05%	0.04%	0.04%	0.02%	0.04%	0.03%	0.04%

tRNA^{Lys} CTT-2.1 (Lib 1 m^{1}A)

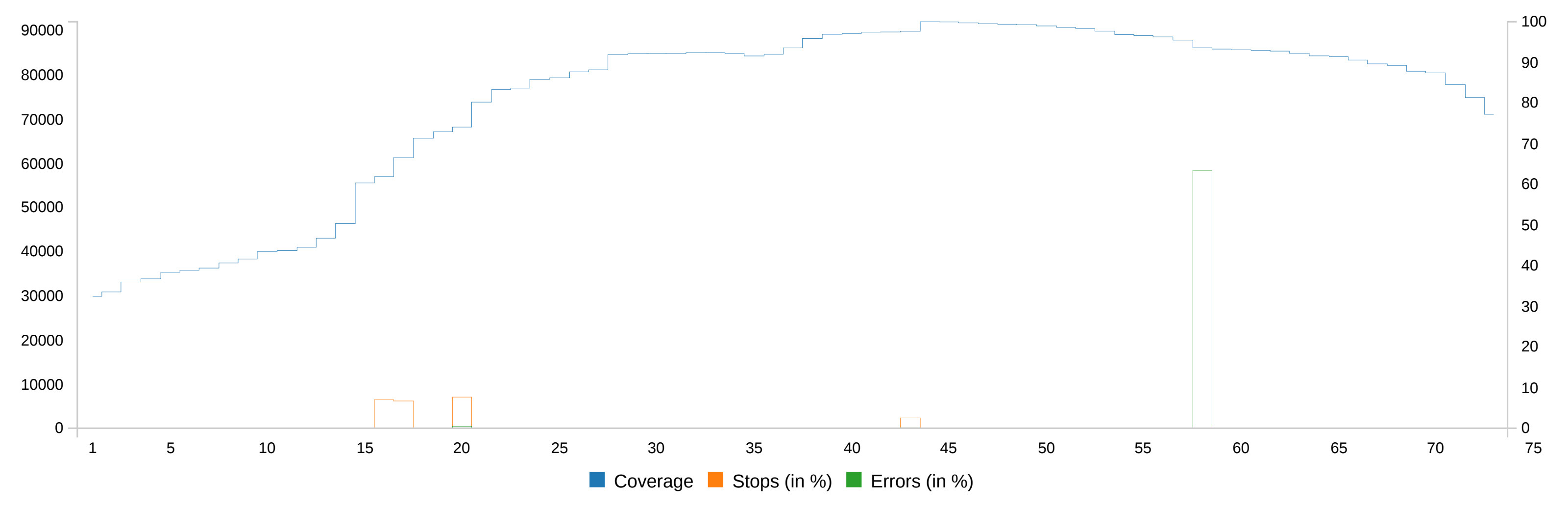

tRNA^{Lys} CTT-2.1 (Lib 2 m^{1}A)

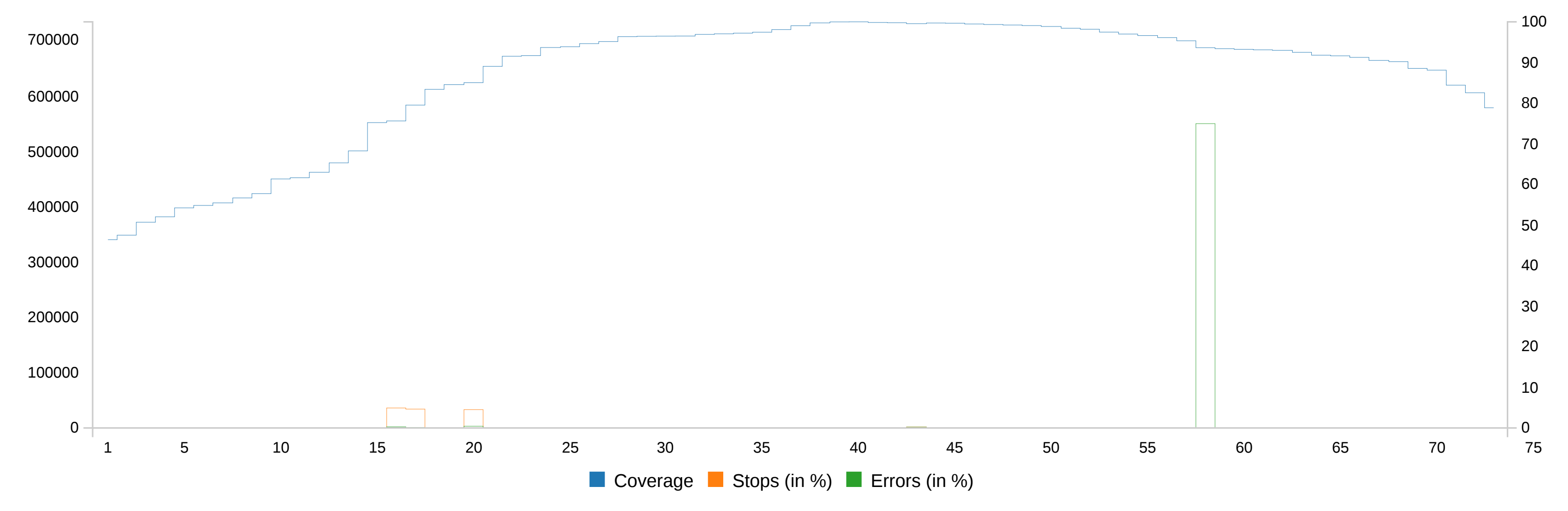

tRNA^{Lys} CTT-2.1 (Lib 3 m^{1}A)

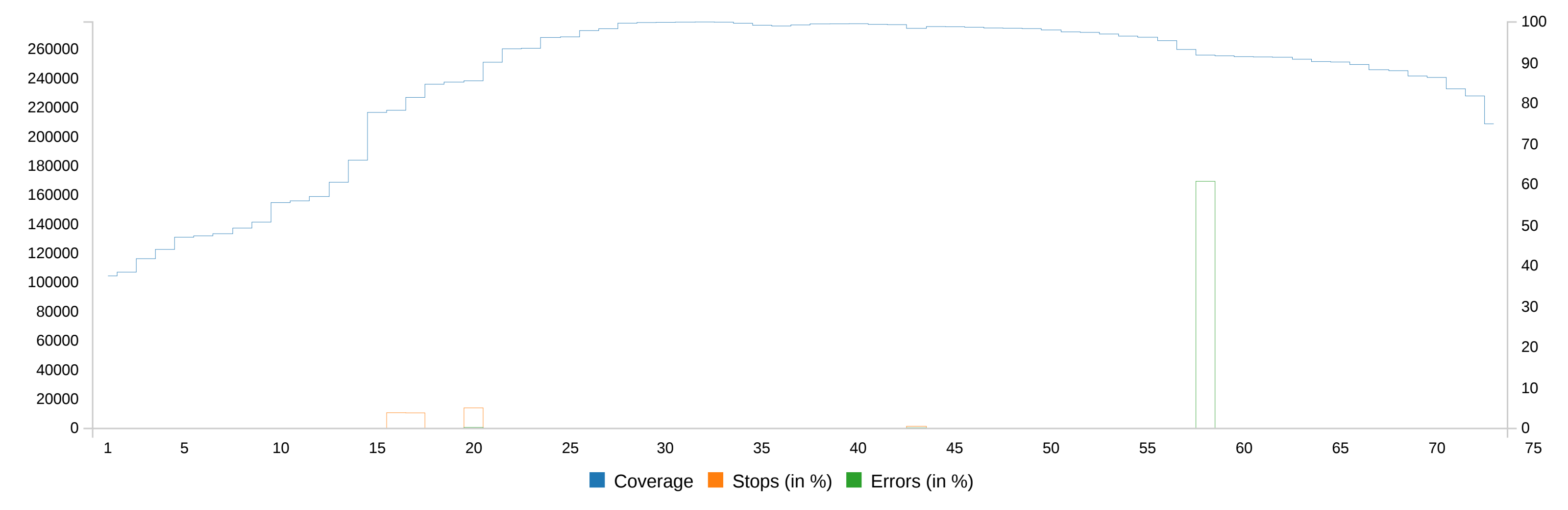

tRNA^{Lys} CTT-2.1 (Lib 4 m^{1}A-T)

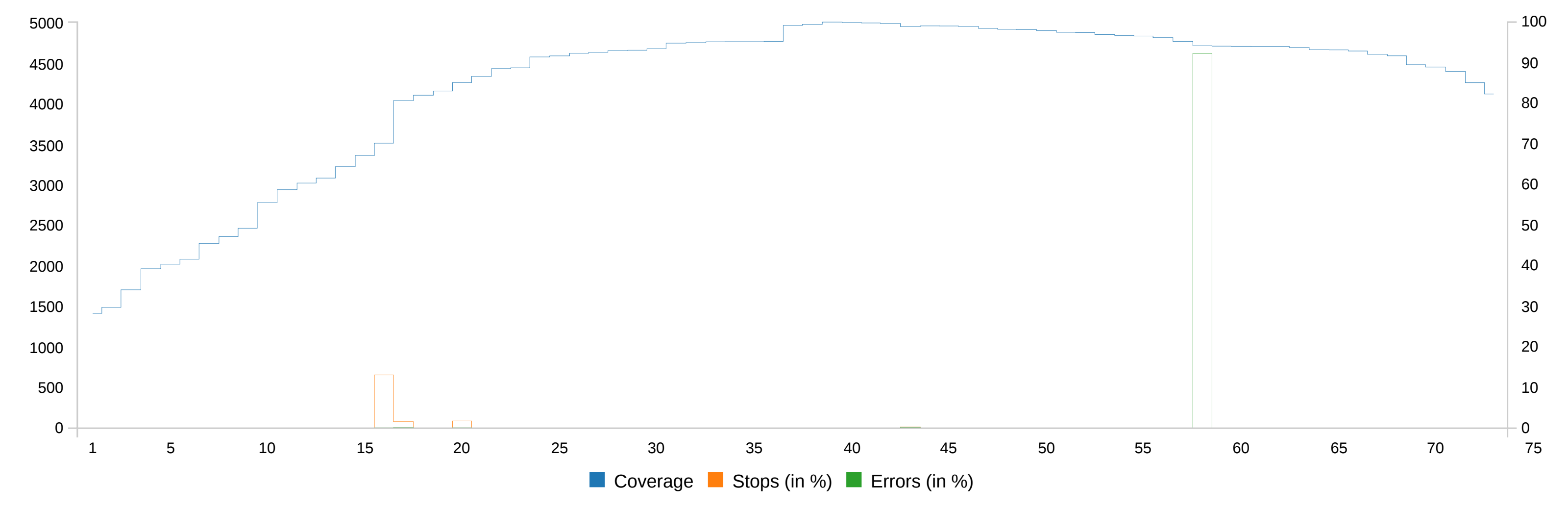

tRNA^{Lys} CTT-2.1 (Lib 1 m^{6}A)

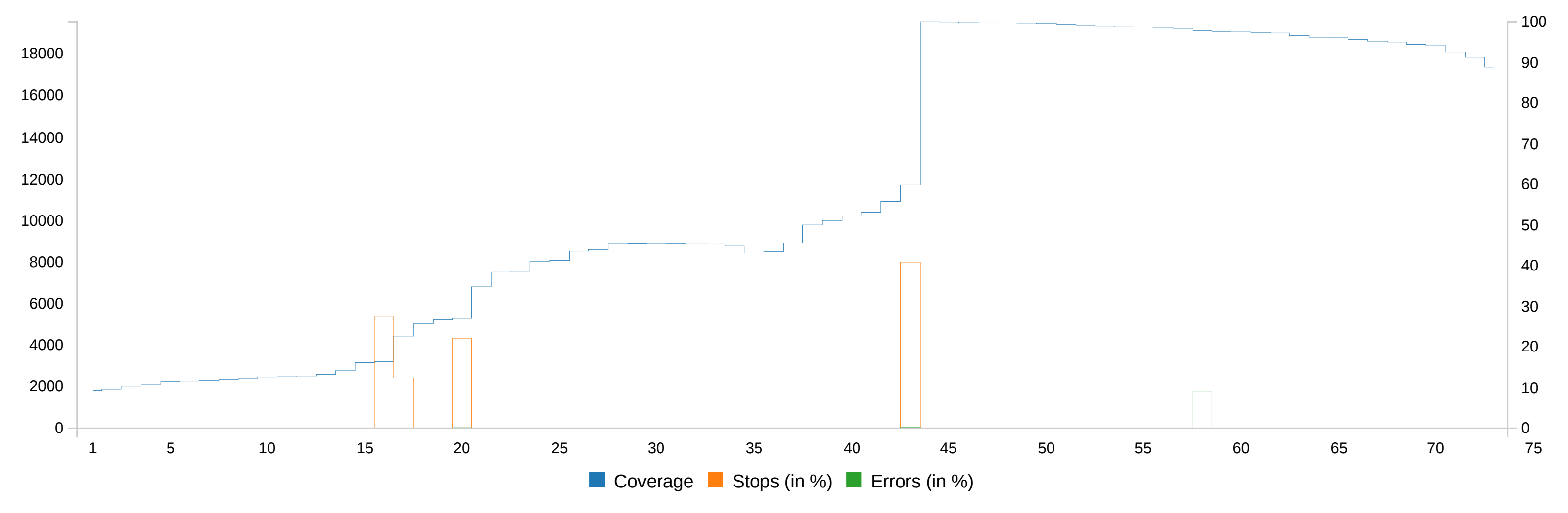

tRNA^{Lys} CTT-2.1 (Lib 2 m^{6}A)

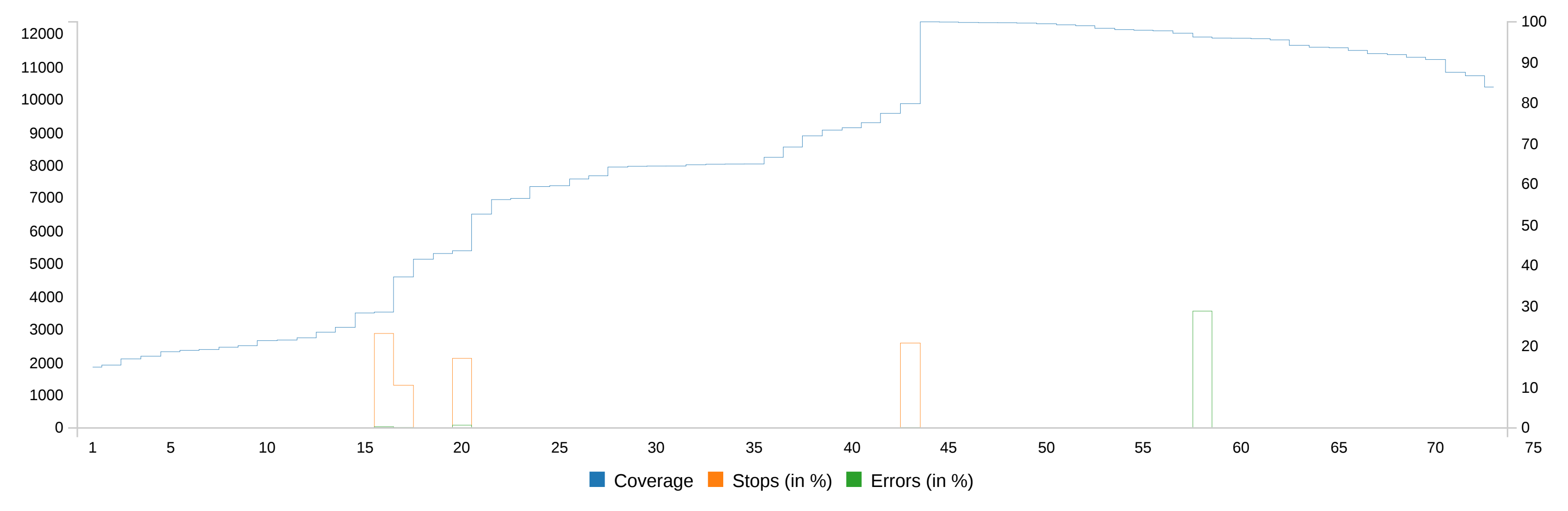

tRNA^{Lys} CTT-2.1 (Lib 3 m^{6}A)

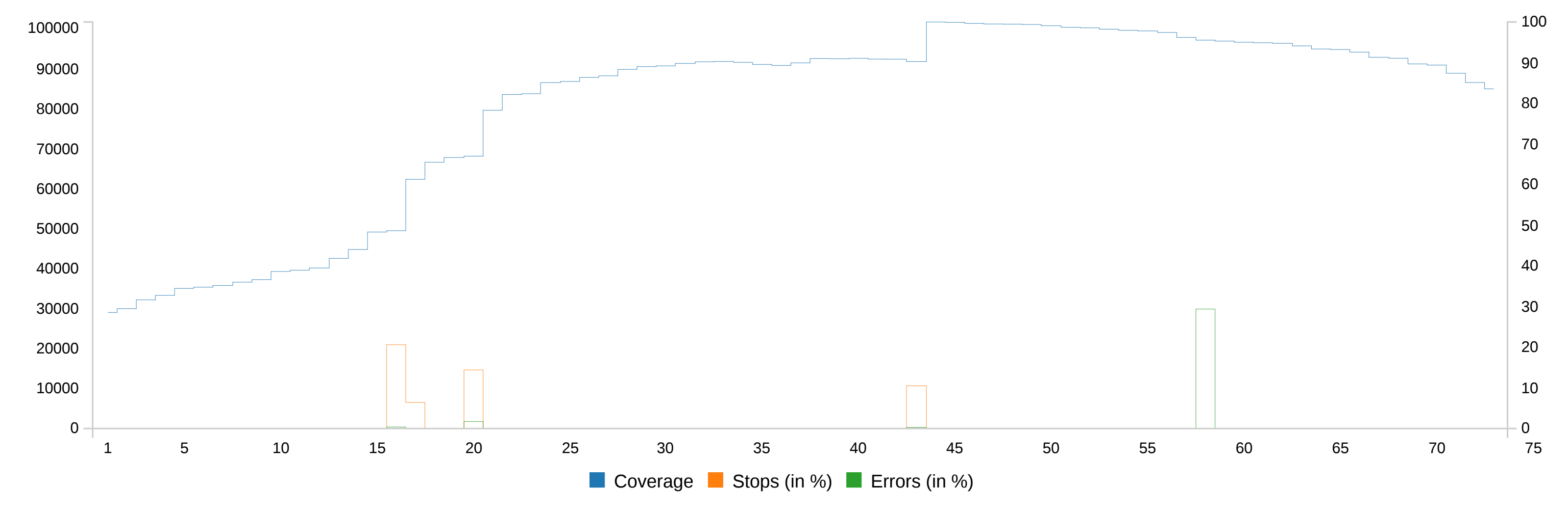

tRNA^{Lys} CTT-2.1 (Lib 4 m^{6}A-T)

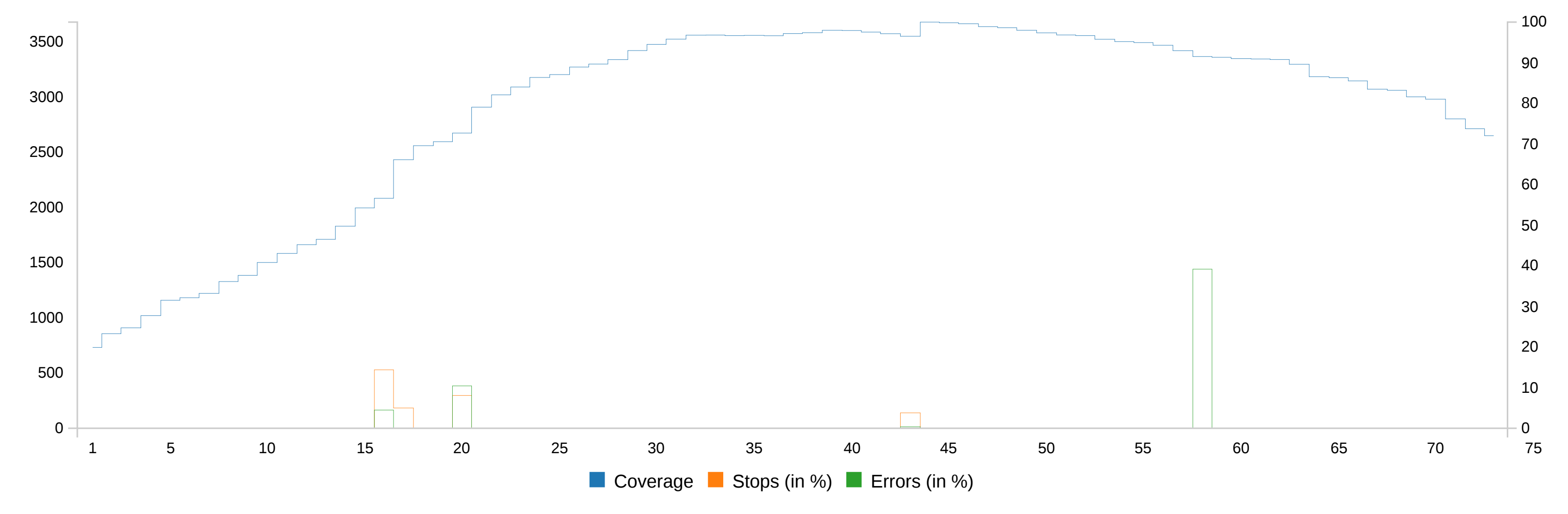

tRNA^{Lys} CTT-1.1

Stops (coverage drops)
Errors (base mismatch)
Pattern: "gttc.A..c"

	Lib 1 m^{1}A	Lib 2 m^{1}A	Lib 3 m^{1}A	Lib 4 m^{1}A-T	Lib 1 m^{6}A	Lib 2 m^{6}A	Lib 3 m^{6}A	Lib 4 m^{6}A-T	p-value
16 (T)	8%	6%	3%	2%	26%	21%	18%	8%	0.01
	0% (C: 0%)	0% (G: 0%)	0% (C: 0%)	0% (C: 0%)	0% (C: 0%)	0% (C: 0%)	0% (C: 0%)	4% (C: 4%)	0.24
	(44706)	(332160)	(141421)	(2688)	(2466)	(2647)	(28329)	(1548)	
20 (T)	9%	5%	6%	12%	25%	19%	16%	9%	0.04
	0% (A: 0%)	0% (G: 0%)	0% (G: 0%)	0% (C: 0%)	0% (A: 0%)	0% (C: 0%)	1% (C: 1%)	4% (C: 4%)	0.15
	(55549)	(389054)	(156798)	(3785)	(4116)	(4042)	(38679)	(1838)	
43 (A)	3%	0%	0%	0%	46%	25%	16%	6%	0.04
	0% (G: 0%)	0% (G: 0%)	0% (G: 0%)	0% (G: 0%)	0% (G: 0%)	0% (G: 0%)	0% (G: 0%)	0% (G: 0%)	0.58
	(75620)	(476831)	(186267)	(4963)	(8984)	(7841)	(54891)	(2290)	
58 (A)	0%	0%	0%	0%	0%	0%	0%	0%	----
	63% (T: 33%)	75% (T: 47%)	65% (T: 43%)	85% (T: 47%)	9% (T: 4%)	26% (T: 16%)	30% (T: 18%)	32% (T: 22%)	0.00
	(72822)	(444359)	(179037)	(4770)	(16390)	(9963)	(62893)	(2287)	

Overall statistics

	Lib 1 m^{1}A	Lib 2 m^{1}A	Lib 3 m^{1}A	Lib 4 m^{1}A-T	Lib 1 m^{6}A	Lib 2 m^{6}A	Lib 3 m^{6}A	Lib 4 m^{6}A-T
Total reads	80511	485680	191335	5098	17679	10879	66290	2573
Average coverage	60224 (σ=20485)	398549 (σ=98307)	157419 (σ=43097)	3943 (σ=1409)	9429 (σ=6056)	6674 (σ=3233)	48582 (σ=16864)	1970 (σ=535)
Average errors	748 (σ=5351)	5771 (σ=38935)	1860 (σ=13551)	66 (σ=473)	29 (σ=181)	47 (σ=305)	337 (σ=2244)	17 (σ=86)
Total errors	1.24%	1.45%	1.18%	1.67%	0.31%	0.72%	0.7%	0.87%
Total "A" errors	0.07%	0.07%	0.07%	0.06%	0.02%	0.04%	0.05%	0.05%

tRNA^{Lys} CTT-1.1 (Lib 1 m^{1}A)

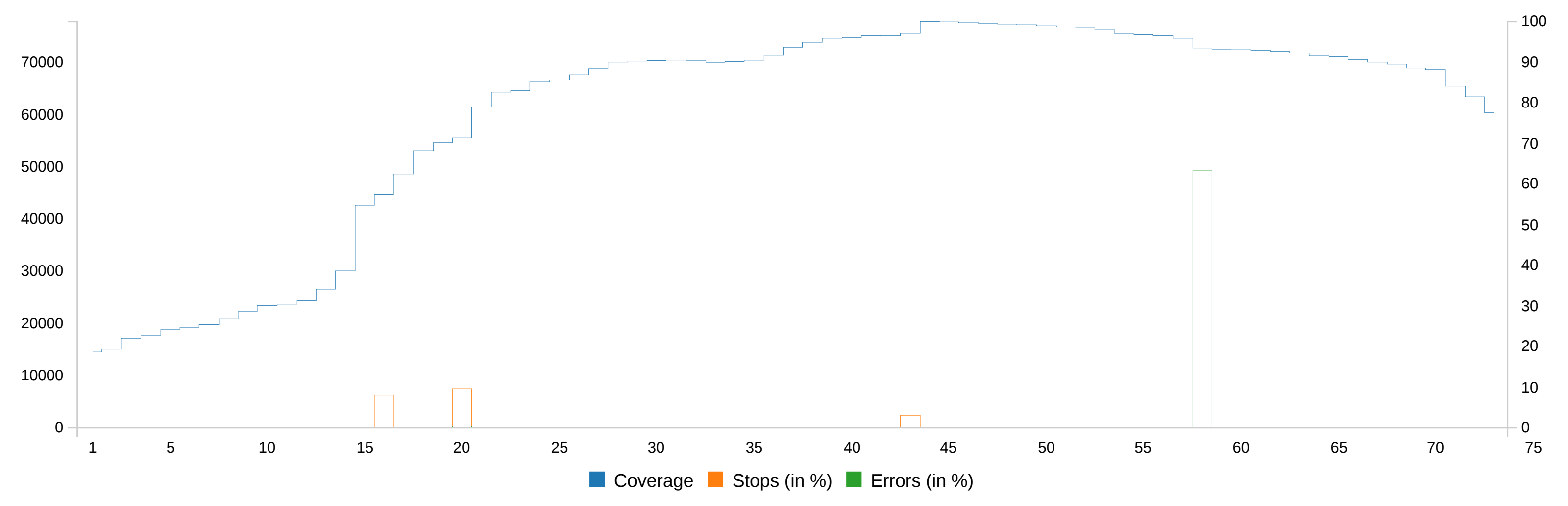

tRNA^{Lys} CTT-1.1 (Lib 2 m^{1}A)

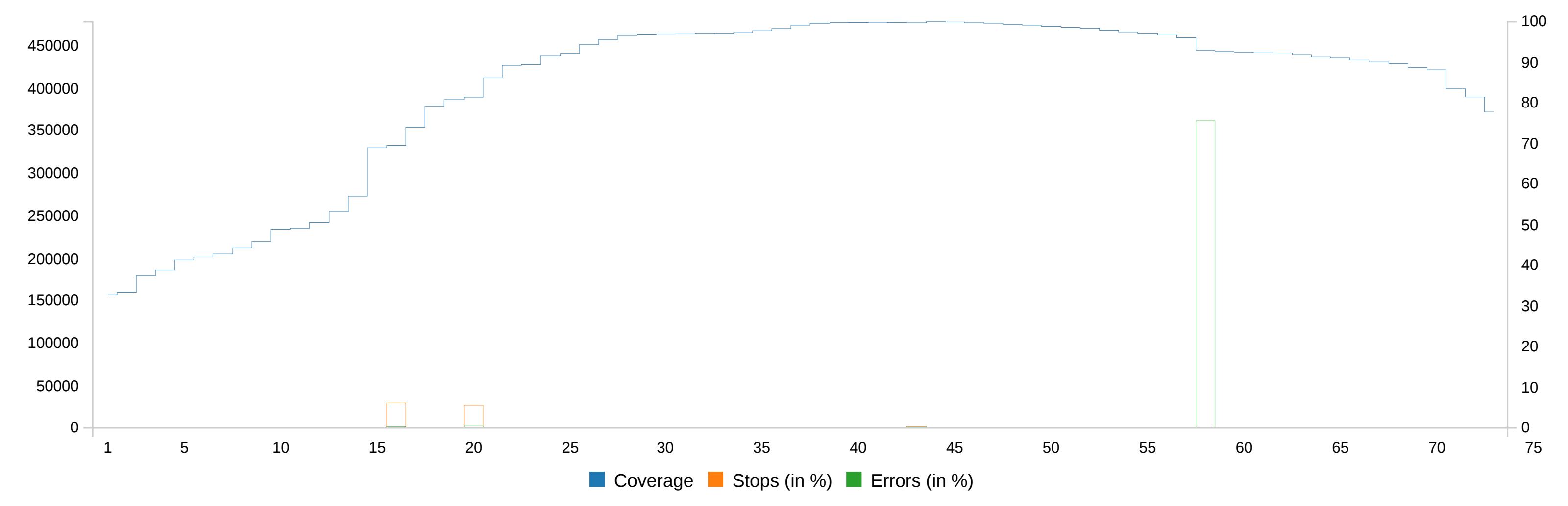

tRNA^{Lys} CTT-1.1 (Lib 3 m^{1}A)

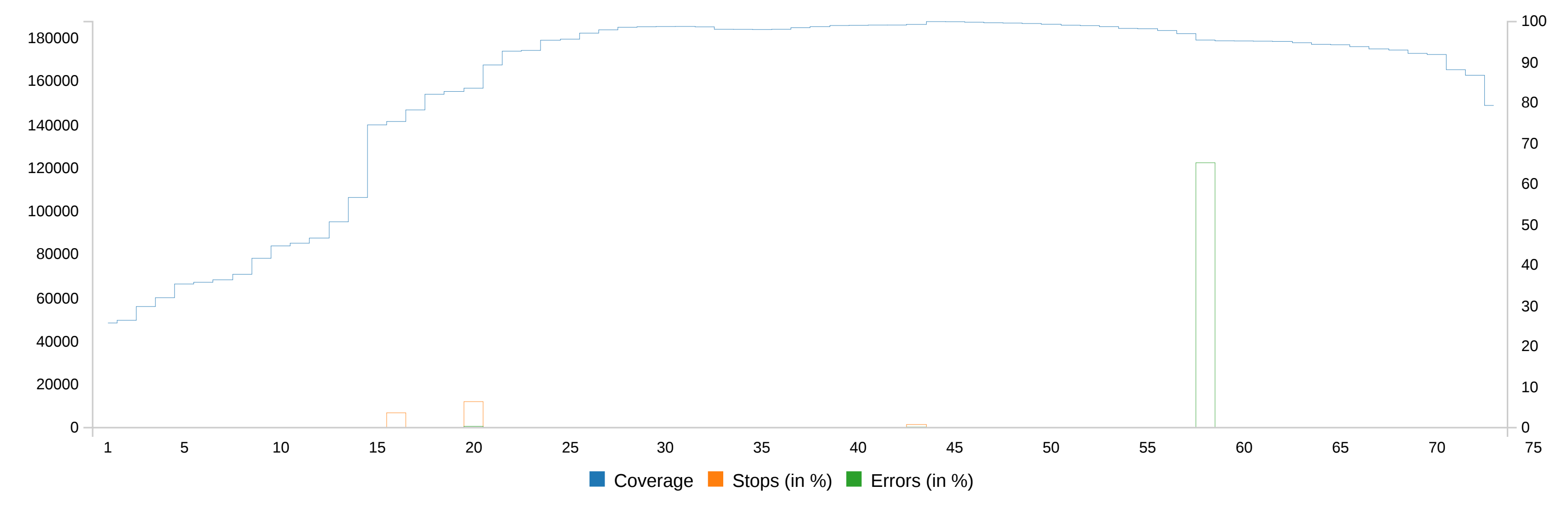

tRNA^{Lys} CTT-1.1 (Lib 4 m^{1}A-T)

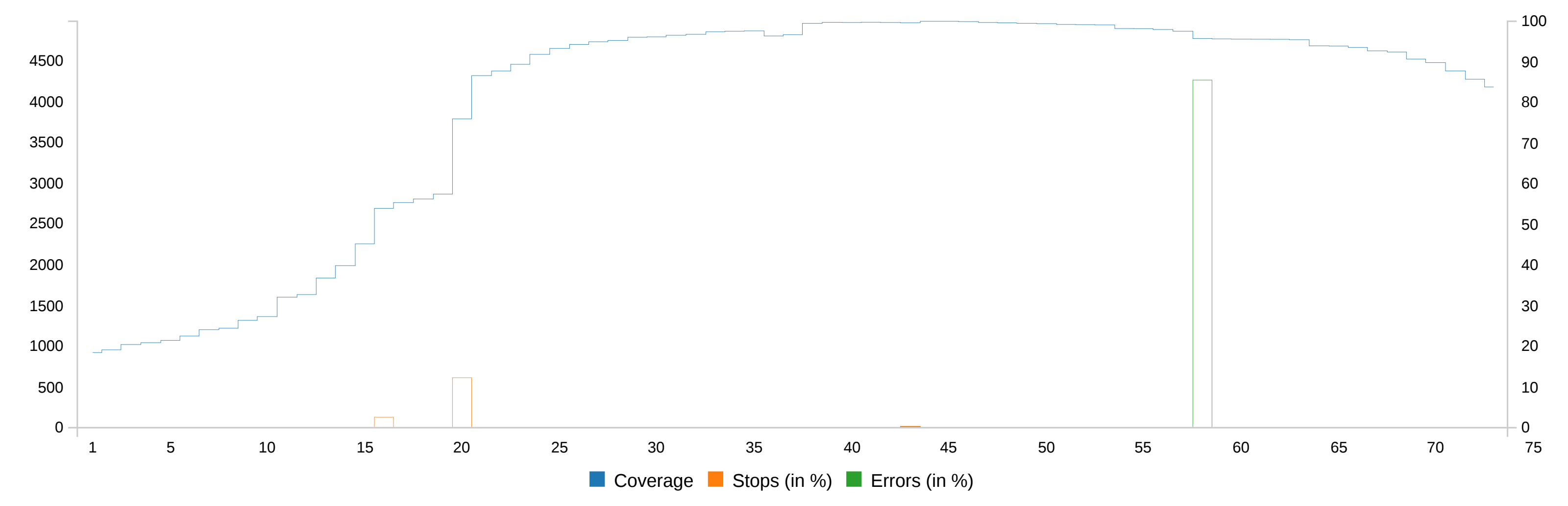

tRNA^{Lys} CTT-1.1 (Lib 1 m^{6}A)

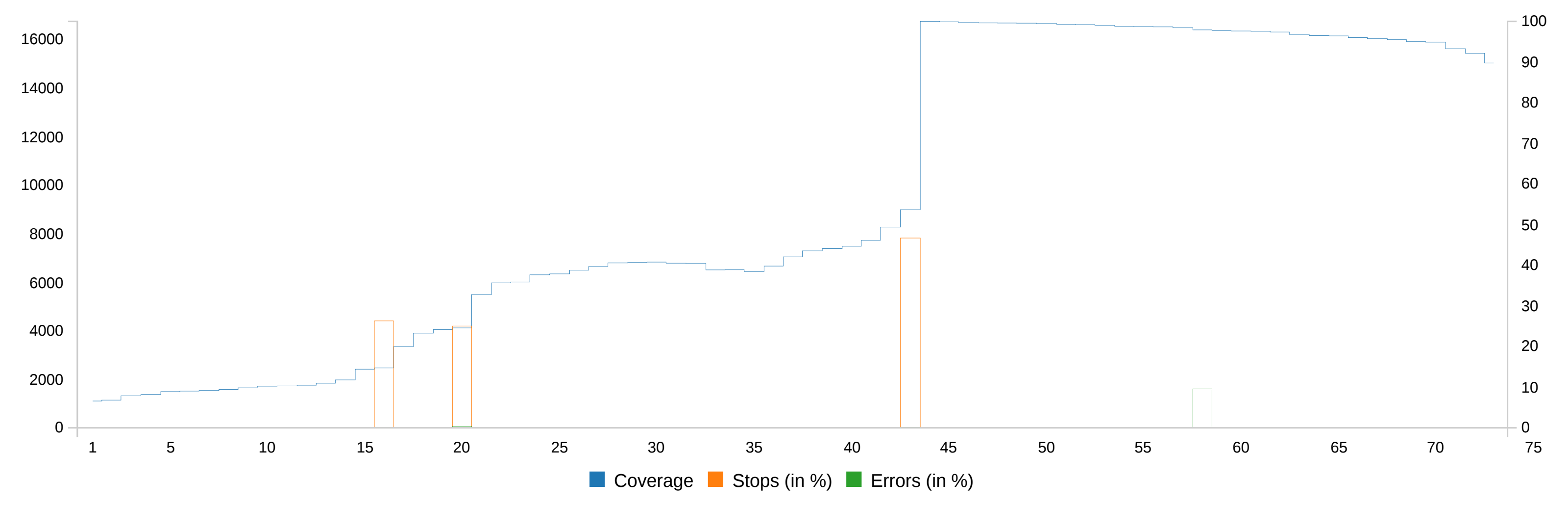

tRNA^{Lys} CTT-1.1 (Lib 2 m^{6}A)

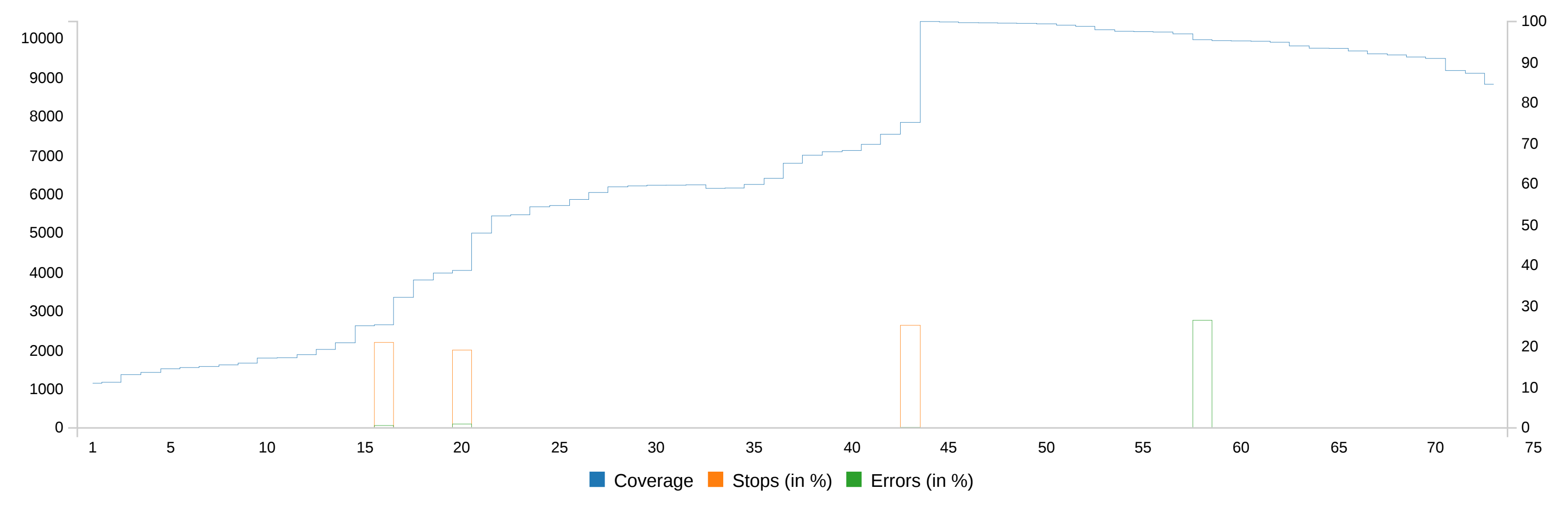

tRNA^{Lys} CTT-1.1 (Lib 3 m^{6}A)

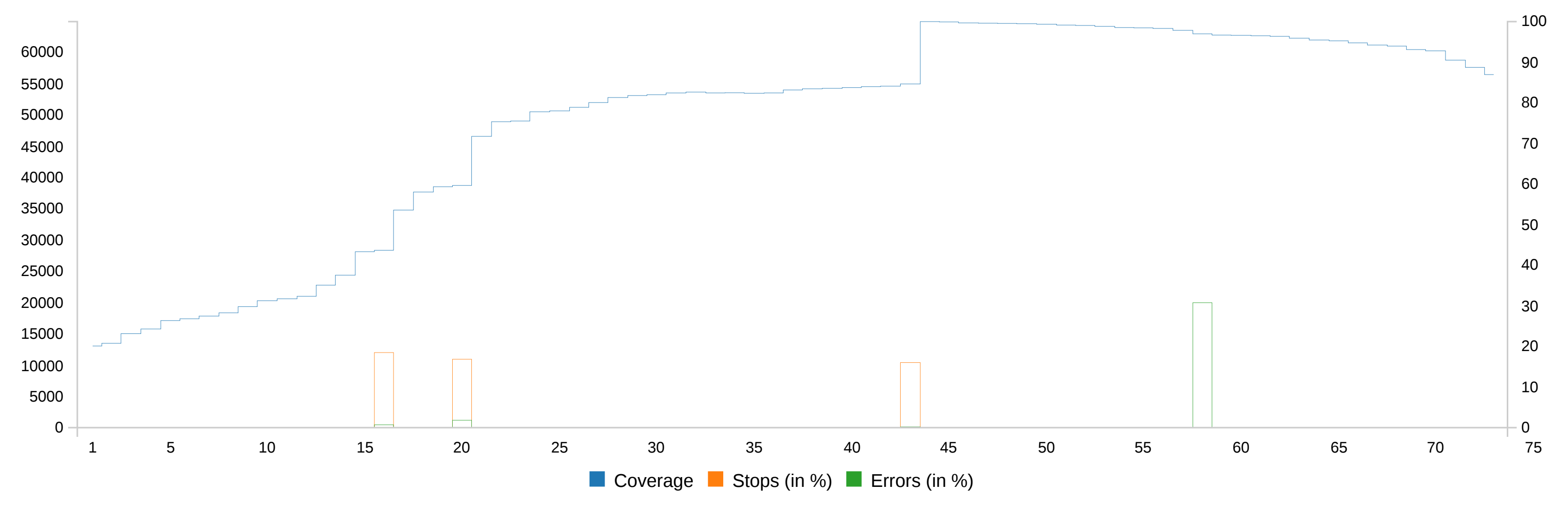

tRNA^{Lys} CTT-1.1 (Lib 4 m^{6}A-T)

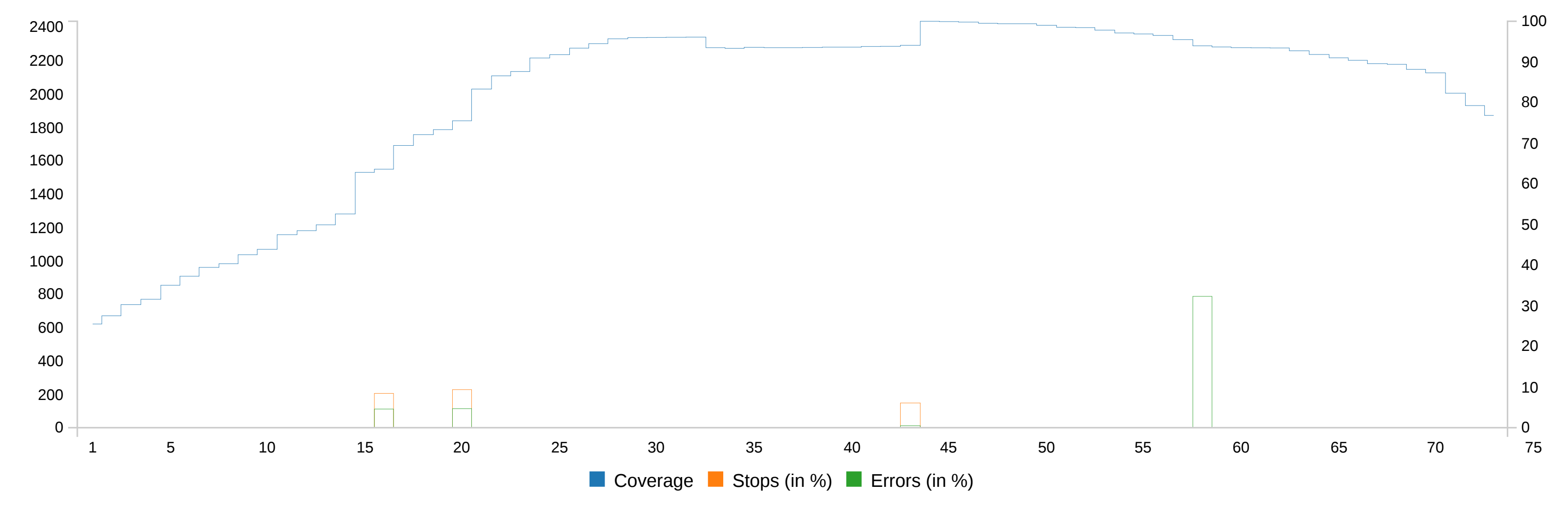

tRNA$^{Asp\ GTC-2.1}$

- Stops (coverage drops)
- Errors (base mismatch)
- Pattern: "gttc.A..c"

	Lib 1 m^{1}A	Lib 2 m^{1}A	Lib 3 m^{1}A	Lib 4 m^{1}A-T	Lib 1 m^{6}A	Lib 2 m^{6}A	Lib 3 m^{6}A	Lib 4 m^{6}A-T	p-value
1 (T)	8%	4%	4%	0%	17%	8%	9%	6%	0.08
	0% (A: 0%)	0% (A: 0%)	0% (A: 0%)	0% (A: 0%)	0% (A: 0%)	0% (A: 0%)	0% (A: 0%)	0% (A: 0%)	----
	(675)	(14440)	(10297)	(657)	(289)	(234)	(7466)	(252)	
9 (A)	77%	87%	85%	55%	45%	65%	47%	44%	0.03
	10% (T: 6%)	0% (G: 0%)	5% (T: 4%)	86% (T: 86%)	0% (T: 0%)	0% (G: 0%)	1% (T: 0%)	11% (T: 11%)	0.32
	(1004)	(19881)	(13543)	(693)	(639)	(467)	(10477)	(380)	
19 (T)	1%	0%	0%	2%	13%	9%	3%	6%	0.02
	0% (C: 0%)	0% (G: 0%)	0% (G: 0%)	0% (C: 0%)	0% (C: 0%)	0% (G: 0%)	0% (C: 0%)	1% (C: 1%)	0.34
	(6022)	(178858)	(107147)	(1729)	(1743)	(1780)	(23314)	(897)	
29 (C)	1%	0%	0%	0%	10%	5%	2%	1%	0.09
	0% (T: 0%)	0% (A: 0%)	0% (G: 0%)	0% (G: 0%)	0% (T: 0%)	0% (A: 0%)	0% (G: 0%)	0% (G: 0%)	0.66
	(6635)	(186458)	(111421)	(1800)	(3038)	(2361)	(26651)	(1041)	
57 (A)	0%	0%	0%	0%	0%	0%	0%	0%	----
	6% (T: 5%)	6% (T: 5%)	5% (T: 5%)	1% (T: 0%)	1% (T: 1%)	3% (T: 2%)	3% (T: 3%)	4% (T: 4%)	0.27
	(7395)	(186952)	(115487)	(2316)	(4759)	(2979)	(30749)	(1156)	

Overall statistics

	Lib 1 m^{1}A	Lib 2 m^{1}A	Lib 3 m^{1}A	Lib 4 m^{1}A-T	Lib 1 m^{6}A	Lib 2 m^{6}A	Lib 3 m^{6}A	Lib 4 m^{6}A-T
Total reads	8263	194464	118463	2520	5308	3367	32076	1243
Average coverage	6134 (σ=2180)	161973 (σ=55946)	98898 (σ=33478)	1887 (σ=537)	3282 (σ=1636)	2310 (σ=928)	25649 (σ=7049)	960 (σ=279)
Average errors	17 (σ=55)	516 (σ=1439)	195 (σ=754)	11 (σ=70)	4 (σ=8)	4 (σ=11)	43 (σ=125)	3 (σ=9)
Total errors	0.29%	0.32%	0.2%	0.63%	0.13%	0.18%	0.17%	0.39%
Total "A" errors	0.04%	0.04%	0.02%	0.05%	0.02%	0.03%	0.02%	0.12%

tRNA$^{Asp\ GTC-2.1}$ (Lib 1 m^{1}A)

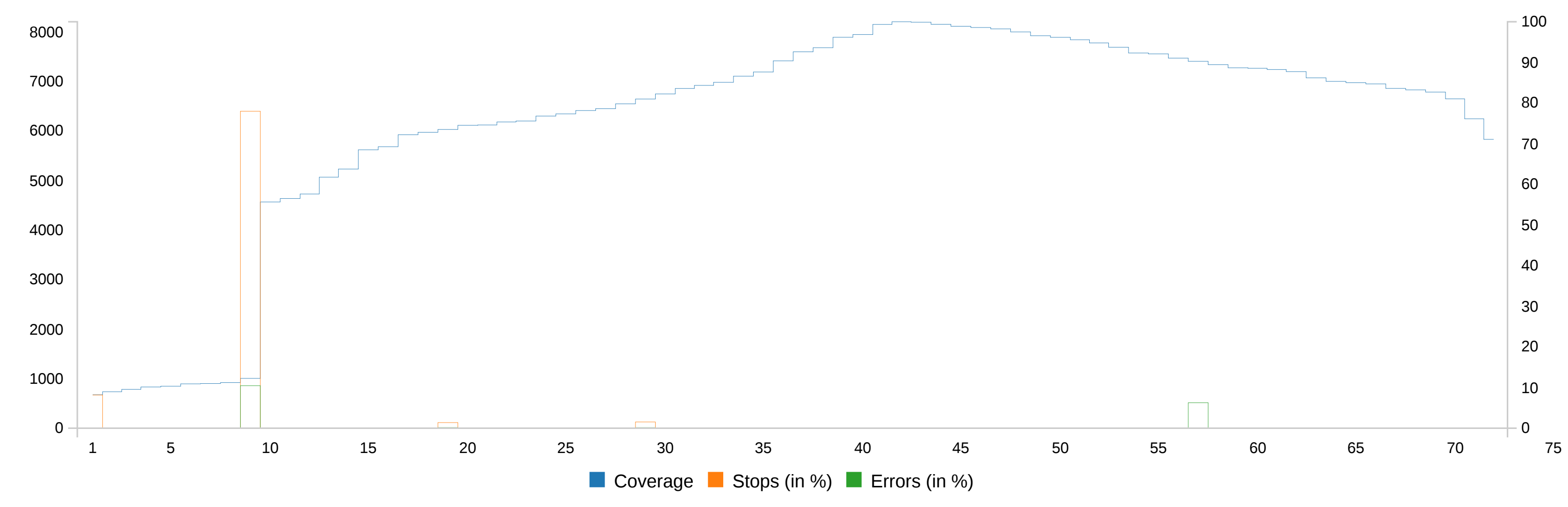

tRNA$^{Asp\ GTC-2.1}$ (Lib 2 m^{1}A)

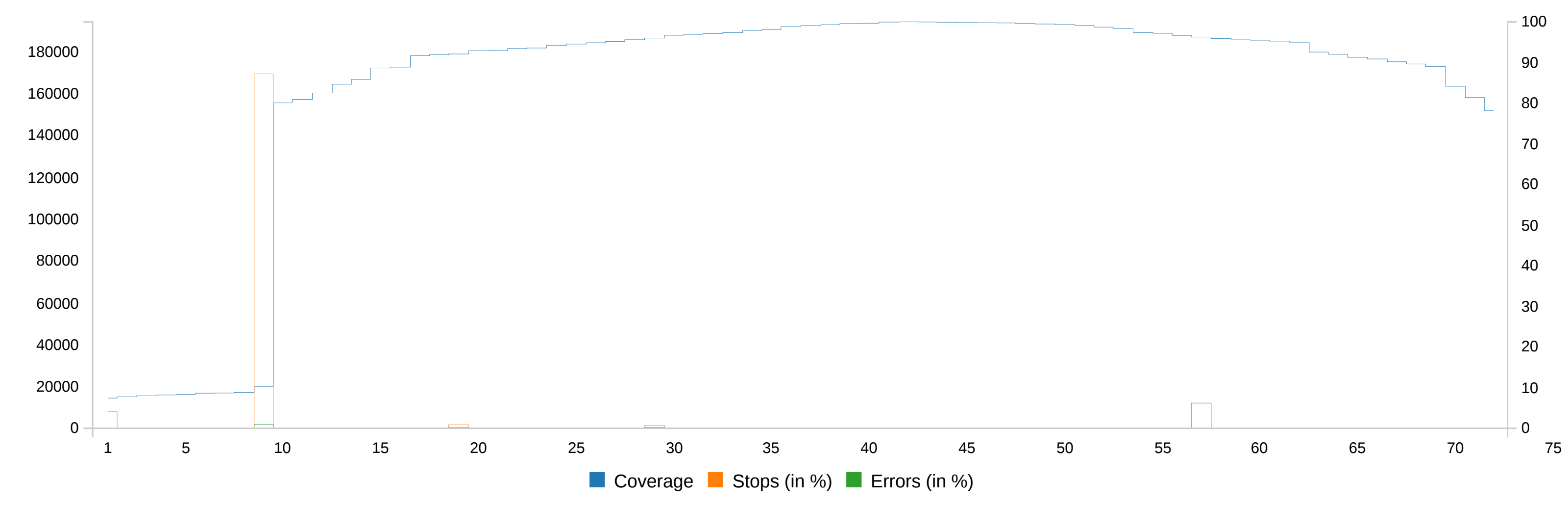

tRNA$^{Asp\ GTC-2.1}$ (Lib 3 m^{1}A)

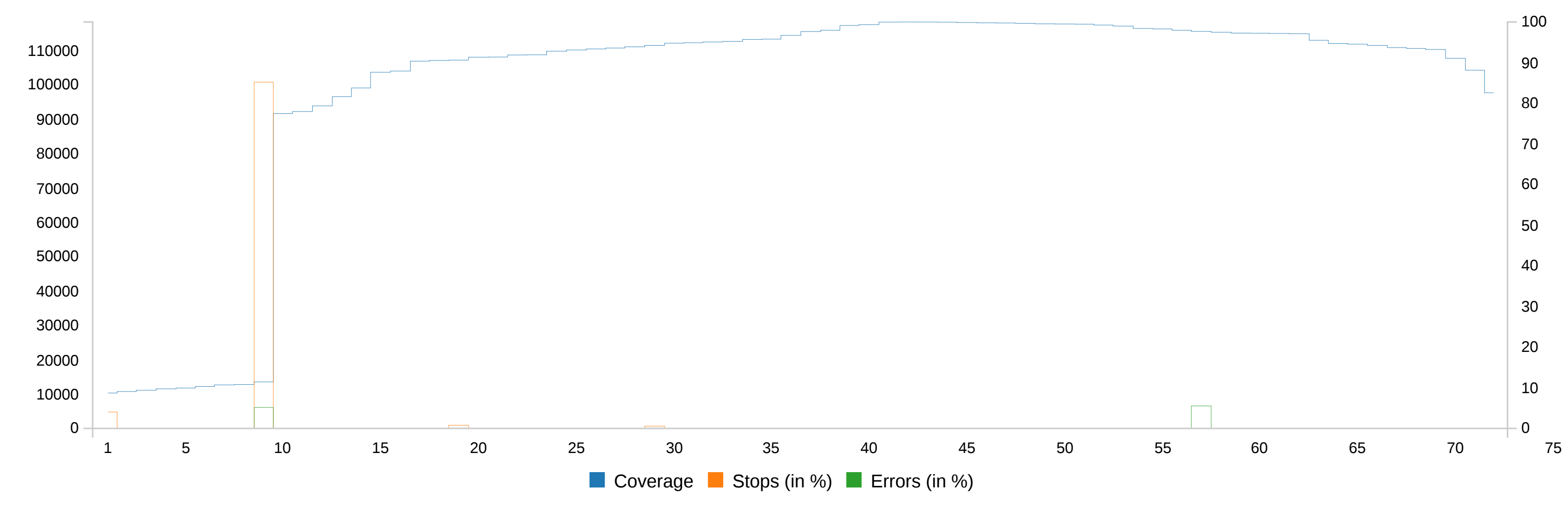

tRNA$^{Asp\ GTC-2.1}$ (Lib 4 m^{1}A-T)

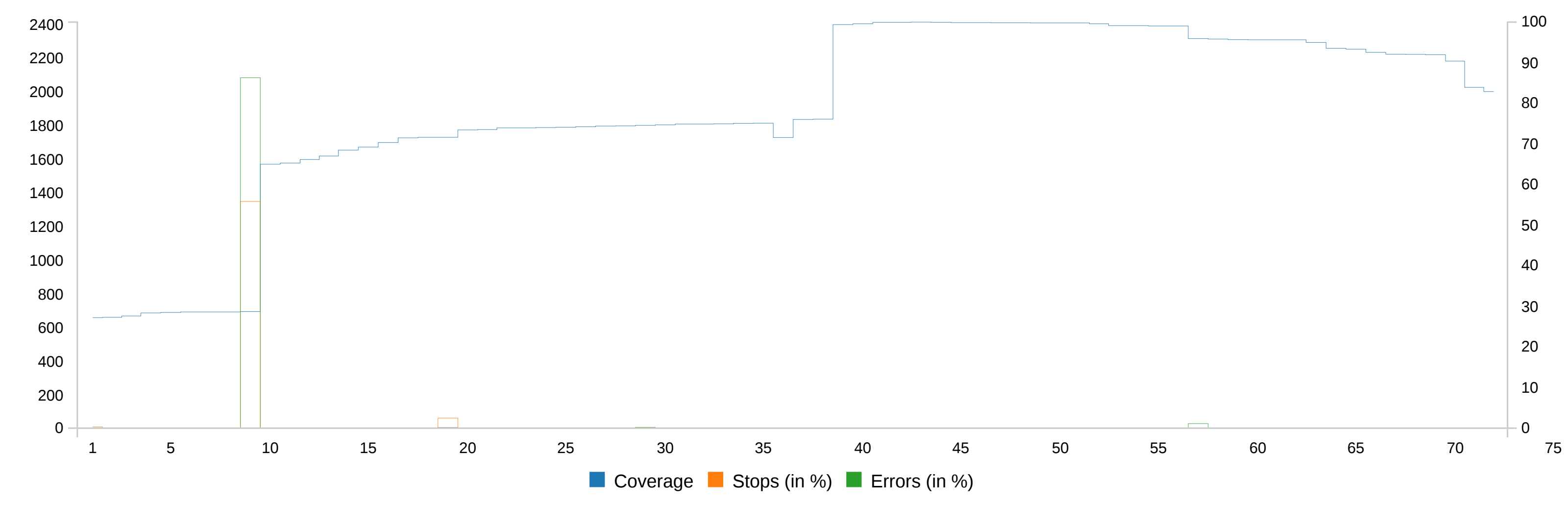

tRNA$^{Asp\ GTC-2.1}$ (Lib 1 m^{6}A)

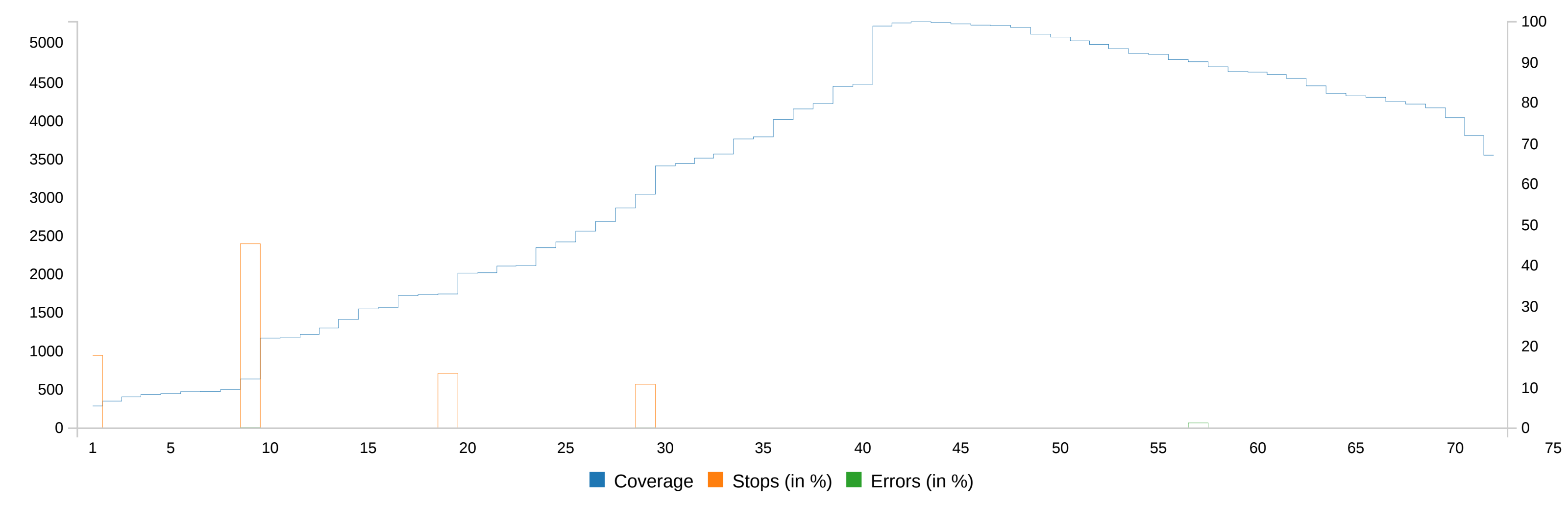

tRNA$^{Asp\ GTC-2.1}$ (Lib 2 m^{6}A)

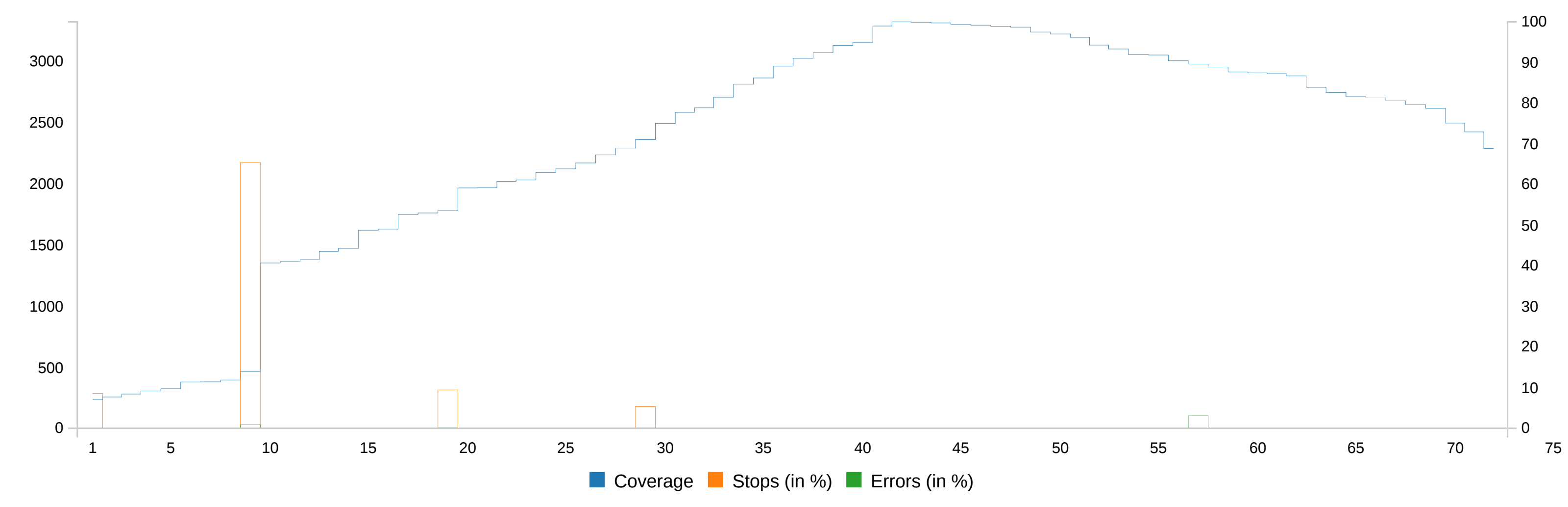

tRNA$^{Asp\ GTC-2.1}$ (Lib 3 m^{6}A)

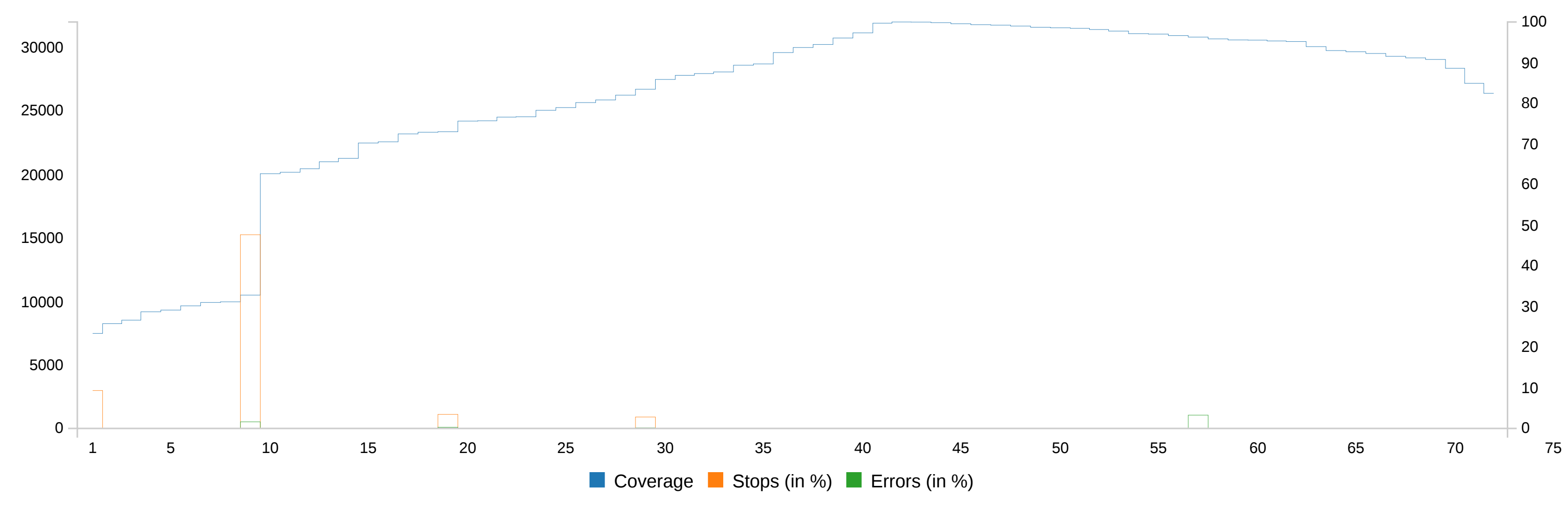

tRNA$^{Asp\ GTC-2.1}$ (Lib 4 m^{6}A-T)

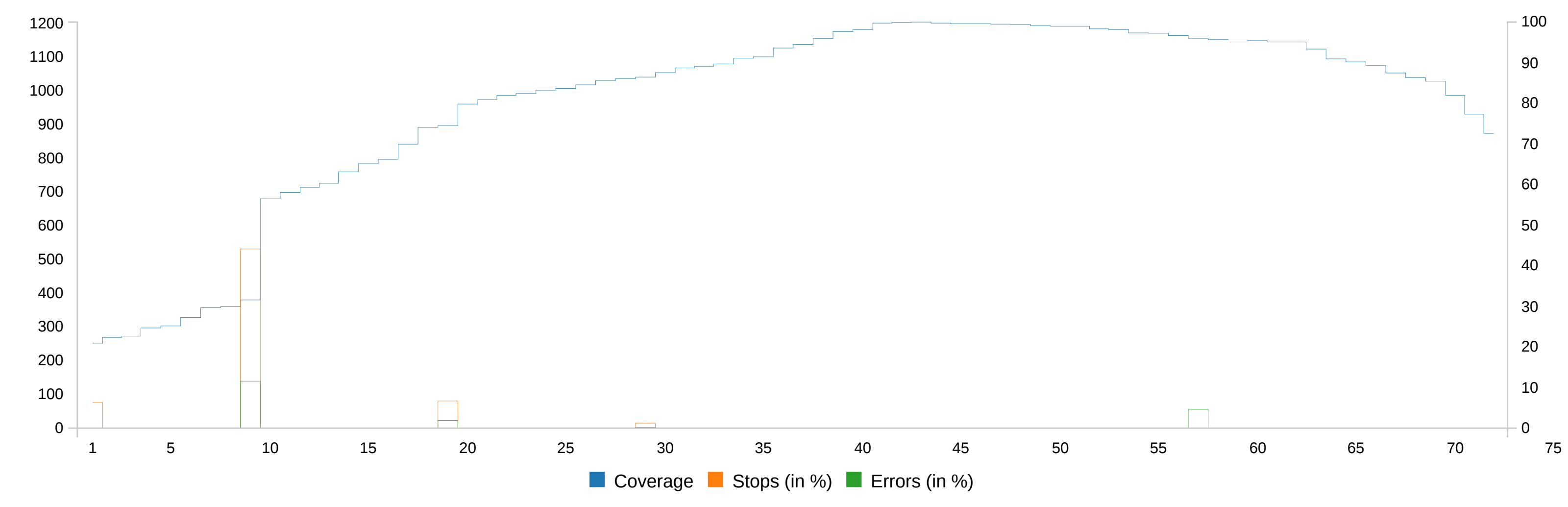

tRNA$^{\text{Asn GTT-2.1}}$

- Stops (coverage drops)
- Errors (base mismatch)
- Pattern: "gttc.A..c"

	Lib 1 m^{1}A	Lib 2 m^{1}A	Lib 3 m^{1}A	Lib 4 m^{1}A-T	Lib 1 m^{6}A	Lib 2 m^{6}A	Lib 3 m^{6}A	Lib 4 m^{6}A-T	p-value
21 (T)	93%	95%	83%	51%	98%	98%	91%	62%	0.64
	2% (A: 2%)	0% (A: 0%)	0% (A: 0%)	28% (C: 28%)	0% (A: 0%)	0% (A: 0%)	0% (A: 0%)	6% (A: 3%)	0.44
	(100)	(116)	(60)	(87)	(2)	(2)	(36)	(30)	
26 (C)	85%	96%	97%	13%	94%	89%	95%	59%	0.62
	0% (G: 0%)	0% (G: 0%)	0% (A: 0%)	0% (A: 0%)	0% (G: 0%)	0% (A: 0%)	0% (A: 0%)	0% (A: 0%)	0.92
	(1573)	(3090)	(385)	(180)	(130)	(142)	(433)	(88)	
27 (G)	10%	4%	2%	48%	5%	10%	5%	47%	0.96
	13% (T: 13%)	2% (T: 2%)	1% (T: 1%)	85% (T: 66%)	4% (T: 4%)	8% (T: 8%)	3% (T: 3%)	35% (A: 22%)	0.58
	(10491)	(81300)	(18112)	(209)	(2230)	(1401)	(10381)	(212)	
40 (C)	2%	0%	0%	0%	18%	9%	3%	4%	0.06
	0% (A: 0%)	0% (A: 0%)	0% (A: 0%)	0% (A: 0%)	0% (T: 0%)	0% (A: 0%)	0% (T: 0%)	0% (A: 0%)	0.10
	(15548)	(108426)	(21795)	(636)	(5232)	(2708)	(13293)	(608)	
59 (A)	0%	0%	0%	0%	0%	0%	0%	0%	----
	70% (G: 37%)	74% (G: 37%)	51% (T: 28%)	91% (G: 59%)	6% (G: 3%)	17% (T: 8%)	23% (T: 13%)	16% (T: 11%)	0.00
	(16178)	(109625)	(22168)	(607)	(8953)	(3604)	(14233)	(648)	

Overall statistics

	Lib 1 m^{1}A	Lib 2 m^{1}A	Lib 3 m^{1}A	Lib 4 m^{1}A-T	Lib 1 m^{6}A	Lib 2 m^{6}A	Lib 3 m^{6}A	Lib 4 m^{6}A-T
Total reads	16309	109928	22348	639	8975	3612	14291	661
Average coverage	9922 (σ=7191)	68474 (σ=50325)	13936 (σ=10226)	390 (σ=259)	4598 (σ=3946)	2004 (σ=1571)	8766 (σ=6444)	385 (σ=280)
Average errors	180 (σ=1322)	1198 (σ=9422)	164 (σ=1324)	11 (σ=66)	10 (σ=62)	13 (σ=76)	63 (σ=396)	3 (σ=15)
Total errors	1.82%	1.75%	1.18%	2.88%	0.24%	0.69%	0.72%	0.83%
Total "A" errors	0.02%	0.02%	0.01%	0.19%	0.01%	0.01%	0.01%	0.2%

tRNA$^{\text{Asn GTT-2.1}}$ (Lib 1 m^{1}A)

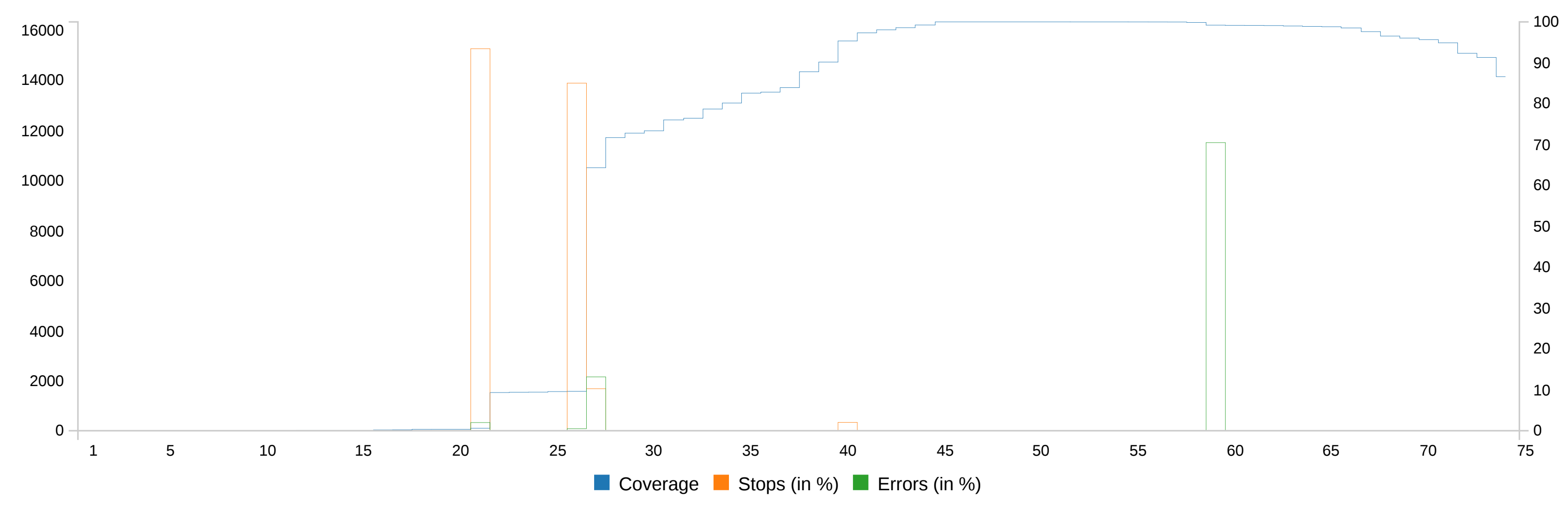

tRNA$^{\text{Asn GTT-2.1}}$ (Lib 2 m^{1}A)

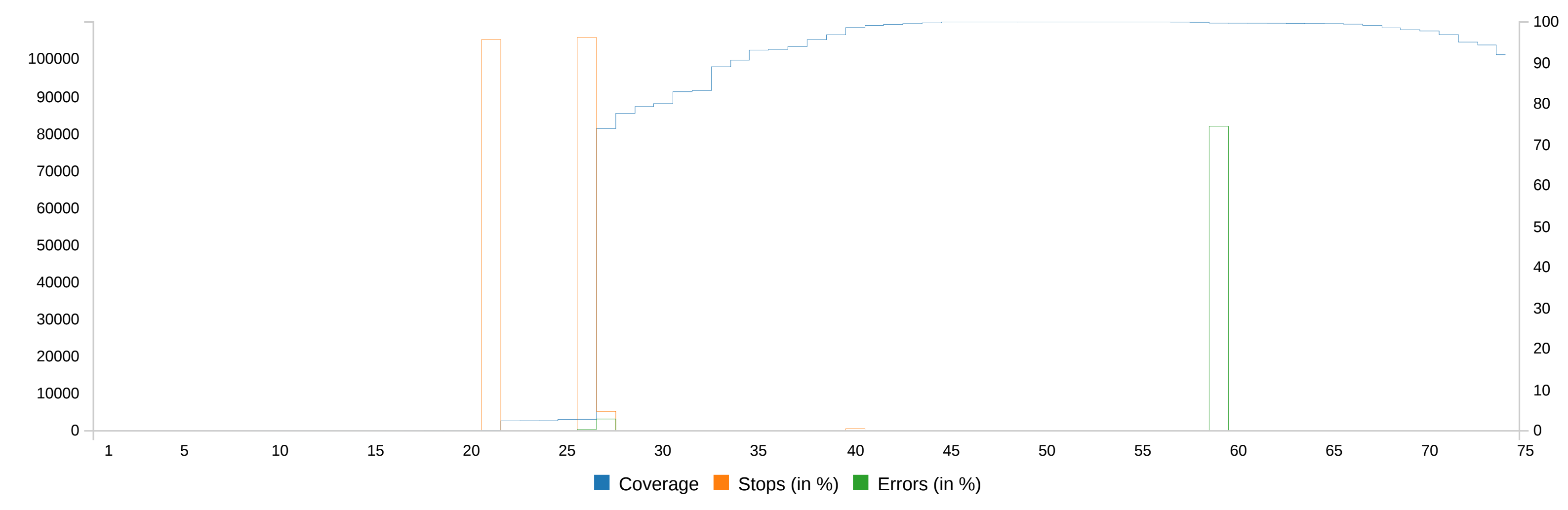

tRNA$^{\text{Asn GTT-2.1}}$ (Lib 3 m^{1}A)

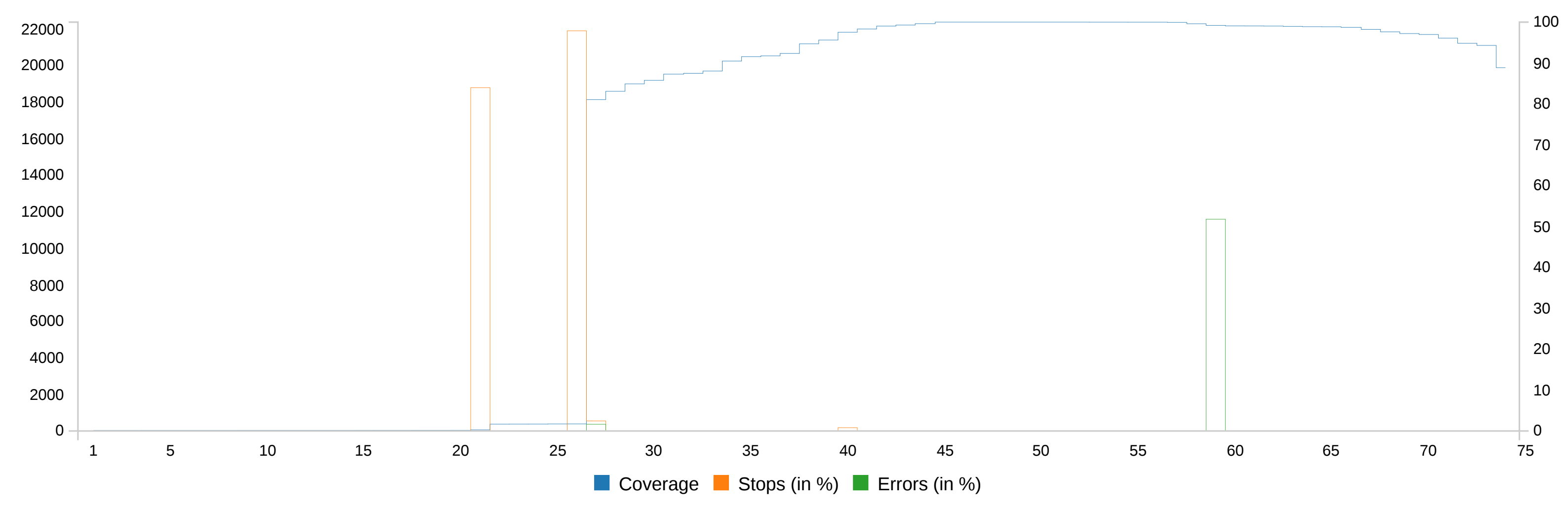

tRNA$^{\text{Asn GTT-2.1}}$ (Lib 4 m^{1}A-T)

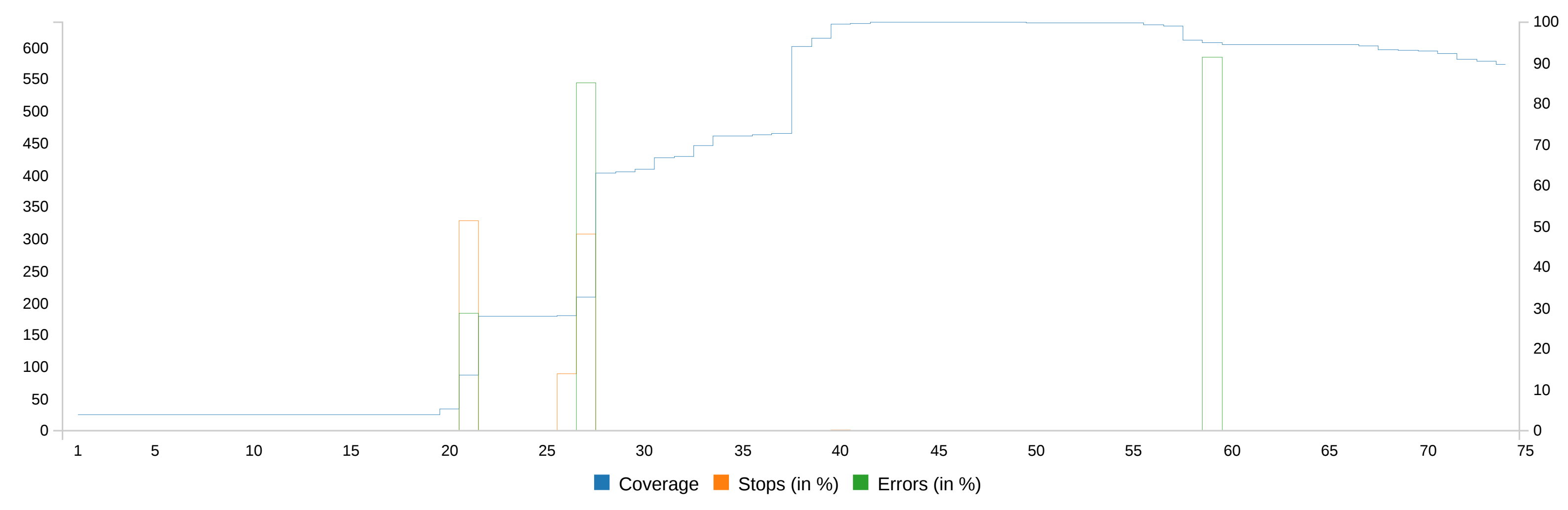

tRNA$^{\text{Asn GTT-2.1}}$ (Lib 1 m^{6}A)

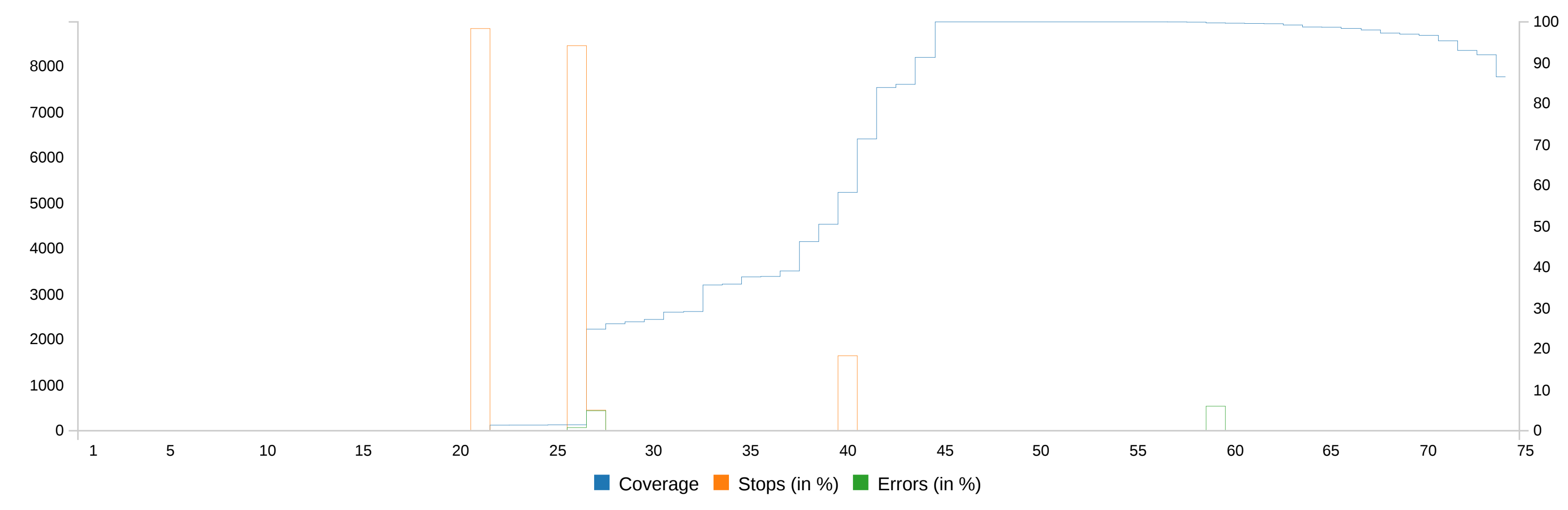

tRNA$^{\text{Asn GTT-2.1}}$ (Lib 2 m^{6}A)

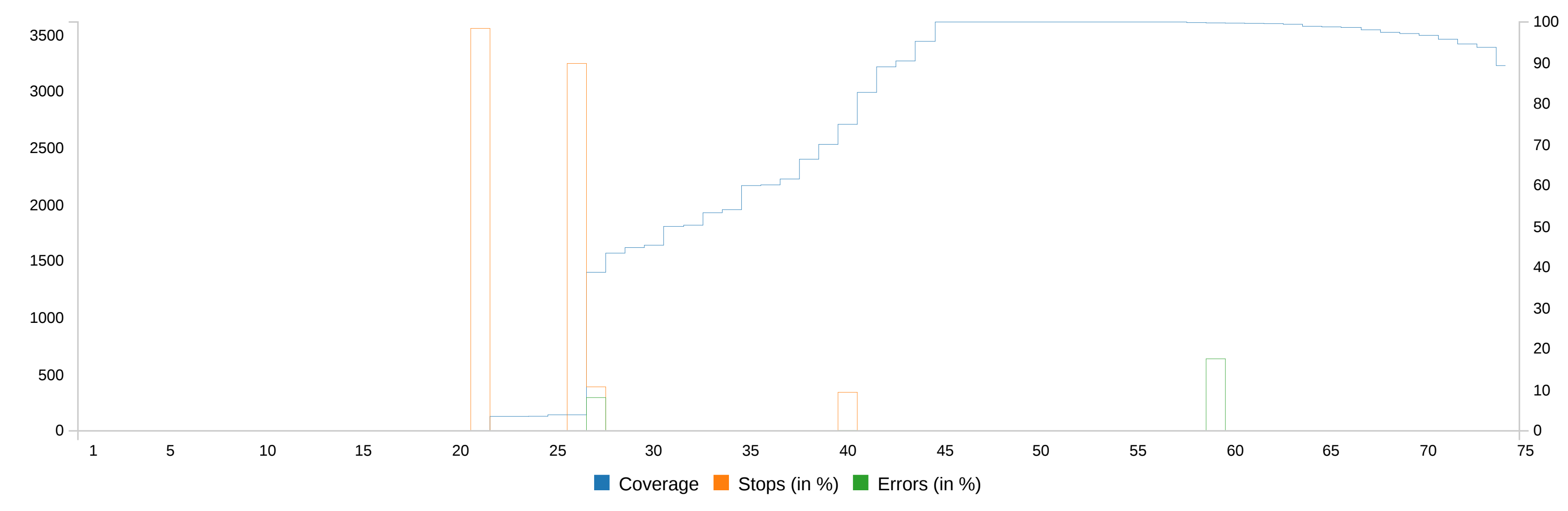

tRNA$^{\text{Asn GTT-2.1}}$ (Lib 3 m^{6}A)

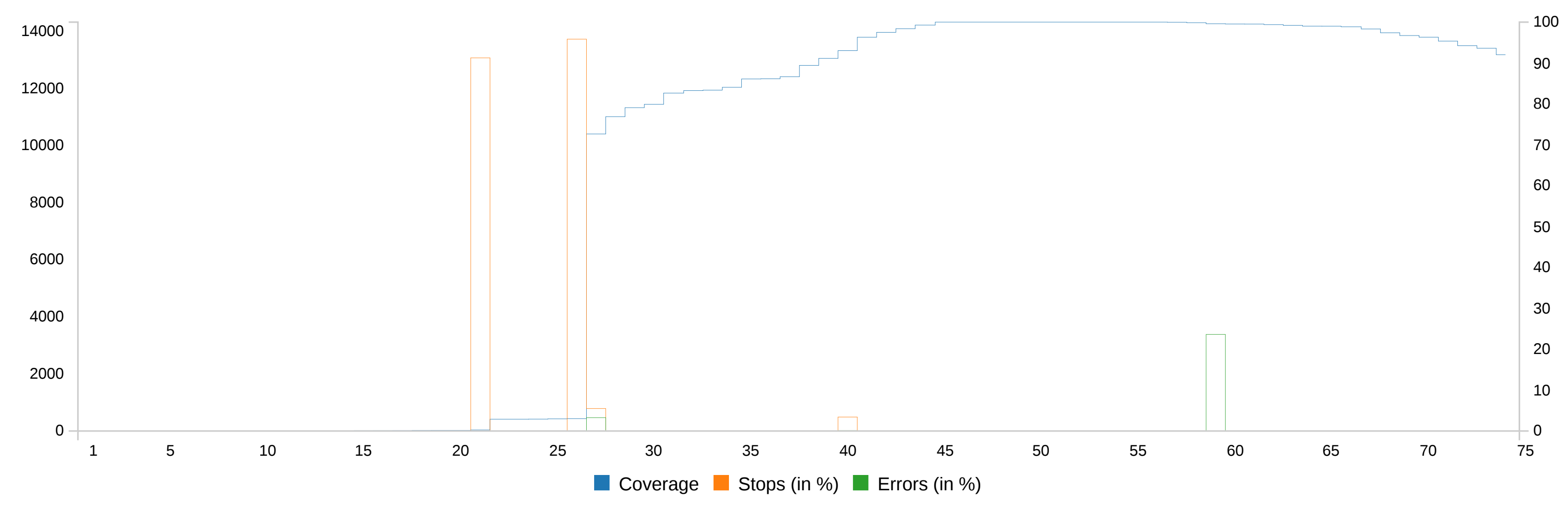

tRNA$^{\text{Asn GTT-2.1}}$ (Lib 4 m^{6}A-T)

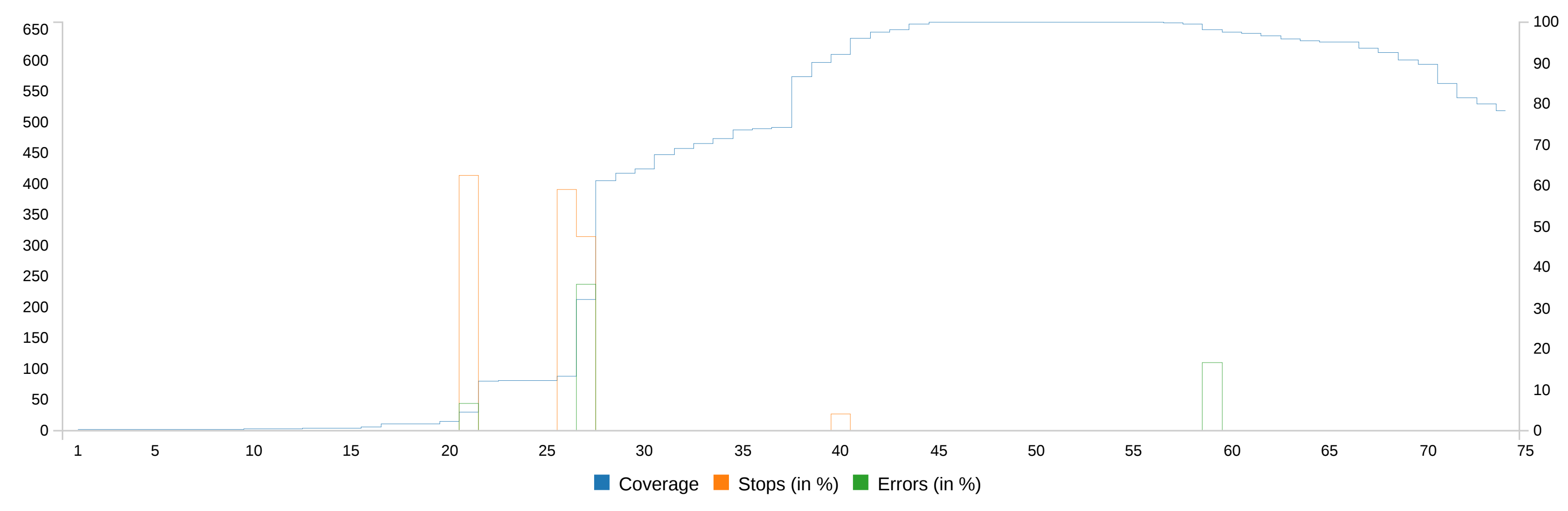

28S rRNA

- Stops (coverage drops)
- Errors (base mismatch)
- Pattern: "gttc.A..c"

	Lib 3 m1A	Lib 4 m1A-T	Lib 3 m6A	Lib 4 m6A-T	p-value
60 (A)	3%	4%	8%	5%	0.24
	79% (G: 79%)	76% (G: 76%)	77% (G: 77%)	80% (G: 80%)	0.61
	(57938)	(1025)	(57552)	(913)	
138 (G)	0%	2%	1%	1%	0.89
	0% (C: 0%)	0% (C: 0%)	0% (C: 0%)	0% (T: 0%)	0.42
	(29385)	(628)	(70964)	(624)	
212 (A)	2%	1%	2%	1%	0.91
	0% (C: 0%)	0% (A: 0%)	0% (G: 0%)	0% (A: 0%)	0.62
	(23904)	(418)	(44387)	(504)	
408 (A)	2%	1%	3%	0%	0.99
	0% (G: 0%)	0% (G: 0%)	0% (G: 0%)	0% (A: 0%)	0.42
	(25840)	(607)	(45538)	(569)	
471 (A)	0%	1%	0%	0%	0.54
	0% (C: 0%)	0% (C: 0%)	0% (C: 0%)	0% (A: 0%)	0.36
	(12952)	(244)	(38696)	(252)	
574 (C)	1%	0%	2%	0%	0.69
	0% (A: 0%)	0% (A: 0%)	0% (A: 0%)	0% (A: 0%)	----
	(280)	(3)	(769)	(1)	
579 (T)	0%	0%	0%	0%	0.64
	0% (G: 0%)	0% (A: 0%)	0% (G: 0%)	0% (A: 0%)	0.60
	(297)	(4)	(858)	(1)	
618 (C)	77%	0%	62%	100%	0.43
	0% (A: 0%)	0% (A: 0%)	0% (A: 0%)	0% (A: 0%)	----
	(18)	(0)	(53)	(0)	
622 (C)	68%	100%	70%	66%	0.43
	0% (A: 0%)	0% (A: 0%)	0% (A: 0%)	0% (A: 0%)	----
	(48)	(0)	(122)	(1)	
628 (C)	74%	77%	55%	55%	0.01
	0% (G: 0%)	0% (A: 0%)	1% (G: 1%)	0% (A: 0%)	0.72
	(220)	(2)	(764)	(4)	
643 (C)	57%	44%	37%	44%	0.32
	0% (G: 0%)	0% (A: 0%)	0% (G: 0%)	0% (A: 0%)	0.99
	(1509)	(16)	(3472)	(20)	
911 (A)	1%	0%	1%	2%	0.21
	1% (C: 1%)	1% (C: 1%)	1% (C: 0%)	1% (C: 1%)	0.61
	(7887)	(74)	(20193)	(108)	
1129 (G)	0%	0%	0%	0%	0.42
	0% (A: 0%)	0% (A: 0%)	0% (A: 0%)	0% (A: 0%)	0.42
	(297)	(3)	(1146)	(2)	
1132 (G)	11%	0%	8%	0%	0.87
	1% (C: 1%)	0% (A: 0%)	1% (C: 1%)	0% (A: 0%)	0.93
	(309)	(3)	(1250)	(3)	
1212 (G)	3%	6%	4%	3%	0.68
	0% (C: 0%)	0% (A: 0%)	0% (T: 0%)	0% (A: 0%)	0.73
	(6480)	(73)	(17009)	(117)	
1317 (A)	82%	54%	31%	33%	0.13
	10% (T: 6%)	32% (G: 29%)	3% (T: 2%)	20% (G: 16%)	0.58
	(5019)	(207)	(39698)	(312)	
1481 (C)	9%	9%	12%	12%	0.01
	0% (G: 0%)	0% (A: 0%)	0% (G: 0%)	0% (A: 0%)	0.88
	(26765)	(523)	(44489)	(634)	
1595 (A)	0%	0%	1%	0%	0.66
	0% (G: 0%)	0% (G: 0%)	0% (G: 0%)	0% (G: 0%)	0.72
	(99407)	(2173)	(128557)	(2052)	
1724 (A)	1%	0%	1%	0%	0.80
	0% (C: 0%)	0% (G: 0%)	0% (T: 0%)	0% (T: 0%)	0.49
	(26752)	(1011)	(33260)	(852)	
2179 (T)	0%	0%	0%	0%	0.42
	1% (C: 1%)	0% (A: 0%)	1% (C: 1%)	0% (A: 0%)	0.97
	(252)	(7)	(1236)	(8)	
2401 (A)	1%	1%	1%	0%	0.73
	0% (C: 0%)	0% (G: 0%)	0% (T: 0%)	0% (G: 0%)	0.89
	(35023)	(901)	(64199)	(803)	
2459 (T)	9%	5%	10%	7%	0.67
	99% (C: 96%)	99% (C: 98%)	98% (C: 96%)	98% (C: 96%)	0.08
	(33121)	(738)	(57659)	(598)	
2987 (C)	3%	0%	3%	0%	0.92
	0% (A: 0%)	0% (A: 0%)	0% (A: 0%)	0% (A: 0%)	----
	(338)	(5)	(1230)	(3)	
3054 (C)	1%	0%	1%	0%	1.00
	0% (A: 0%)	0% (A: 0%)	0% (A: 0%)	0% (A: 0%)	----
	(774)	(4)	(2444)	(6)	
3057 (C)	2%	20%	1%	0%	0.38
	1% (T: 1%)	0% (A: 0%)	1% (T: 1%)	0% (A: 0%)	0.99
	(778)	(4)	(2347)	(6)	
3169 (C)	37%	58%	14%	18%	0.09
	0% (T: 0%)	0% (A: 0%)	0% (A: 0%)	0% (A: 0%)	0.93
	(450)	(5)	(2142)	(9)	
3223 (C)	10%	8%	6%	5%	0.06
	0% (G: 0%)	0% (A: 0%)	0% (G: 0%)	0% (A: 0%)	0.86
	(2851)	(32)	(7133)	(38)	
3318 (C)	0%	0%	1%	0%	0.65
	0% (A: 0%)	0% (A: 0%)	0% (G: 0%)	0% (A: 0%)	0.42
	(209)	(6)	(804)	(3)	
3320 (N)	6%	0%	5%	0%	0.87
	87% (C: 86%)	100% (C: 100%)	91% (C: 90%)	100% (C: 100%)	0.83
	(200)	(6)	(775)	(3)	
3420 (C)	0%	0%	0%	0%	----
	0% (A: 0%)	0% (A: 0%)	0% (A: 0%)	0% (A: 0%)	----
	(200)	(3)	(785)	(0)	
3449 (C)	3%	0%	4%	0%	0.88
	0% (G: 0%)	0% (A: 0%)	0% (G: 0%)	0% (A: 0%)	0.51
	(216)	(3)	(594)	(4)	
3508 (C)	13%	14%	16%	16%	0.04
	0% (T: 0%)	0% (A: 0%)	0% (A: 0%)	0% (A: 0%)	0.73
	(537)	(6)	(1473)	(10)	
3526 (C)	47%	25%	23%	37%	0.70
	0% (A: 0%)	0% (A: 0%)	0% (A: 0%)	0% (A: 0%)	0.98
	(804)	(17)	(2041)	(15)	
3581 (A)	9%	10%	6%	7%	0.05
	0% (G: 0%)	0% (A: 0%)	0% (G: 0%)	0% (A: 0%)	0.59
	(10128)	(166)	(15783)	(200)	
4150 (C)	10%	9%	15%	14%	0.02
	0% (A: 0%)	0% (A: 0%)	0% (T: 0%)	0% (A: 0%)	0.84
	(6891)	(194)	(14455)	(203)	
4347 (A)	2%	1%	2%	2%	0.35
	0% (T: 0%)	0% (T: 0%)	0% (C: 0%)	1% (C: 1%)	0.53
	(31816)	(677)	(45144)	(627)	
4544 (A)	3%	1%	2%	0%	0.48
	0% (C: 0%)	0% (C: 0%)	0% (C: 0%)	0% (C: 0%)	0.56
	(37892)	(697)	(52932)	(717)	
4808 (C)	25%	20%	11%	9%	0.05
	0% (A: 0%)	20% (G: 20%)	0% (A: 0%)	0% (A: 0%)	0.42
	(476)	(5)	(1289)	(10)	
4810 (C)	50%	64%	41%	41%	0.14
	0% (A: 0%)	0% (A: 0%)	0% (G: 0%)	0% (A: 0%)	0.42
	(630)	(5)	(1430)	(11)	
4814 (A)	28%	26%	19%	20%	0.03
	0% (C: 0%)	0% (A: 0%)	0% (G: 0%)	0% (A: 0%)	0.69
	(1260)	(14)	(2451)	(19)	
4843 (C)	13%	13%	19%	19%	0.00
	0% (G: 0%)	0% (A: 0%)	0% (T: 0%)	0% (A: 0%)	0.63
	(6978)	(94)	(9424)	(106)	
4890 (T)	19%	14%	7%	9%	0.10
	95% (C: 93%)	81% (C: 80%)	95% (C: 94%)	89% (C: 87%)	0.61
	(13635)	(276)	(25726)	(398)	

Overall statistics

	Lib 3 m^1A	Lib 4 m^1A-T	Lib 3 m^6A	Lib 4 m^6A-T
Total reads	1701660	32120	2913620	29465
Average coverage	28689 (σ=30546)	540 (σ=513)	42174 (σ=31623)	541 (σ=484)
Average errors	52 (σ=831)	1 (σ=16)	87 (σ=1098)	1 (σ=14)
Total errors	0.18%	0.34%	0.21%	0.33%
Total "A" errors	0.03%	0.08%	0.03%	0.08%

28S rRNA (Lib 3 m^1A)

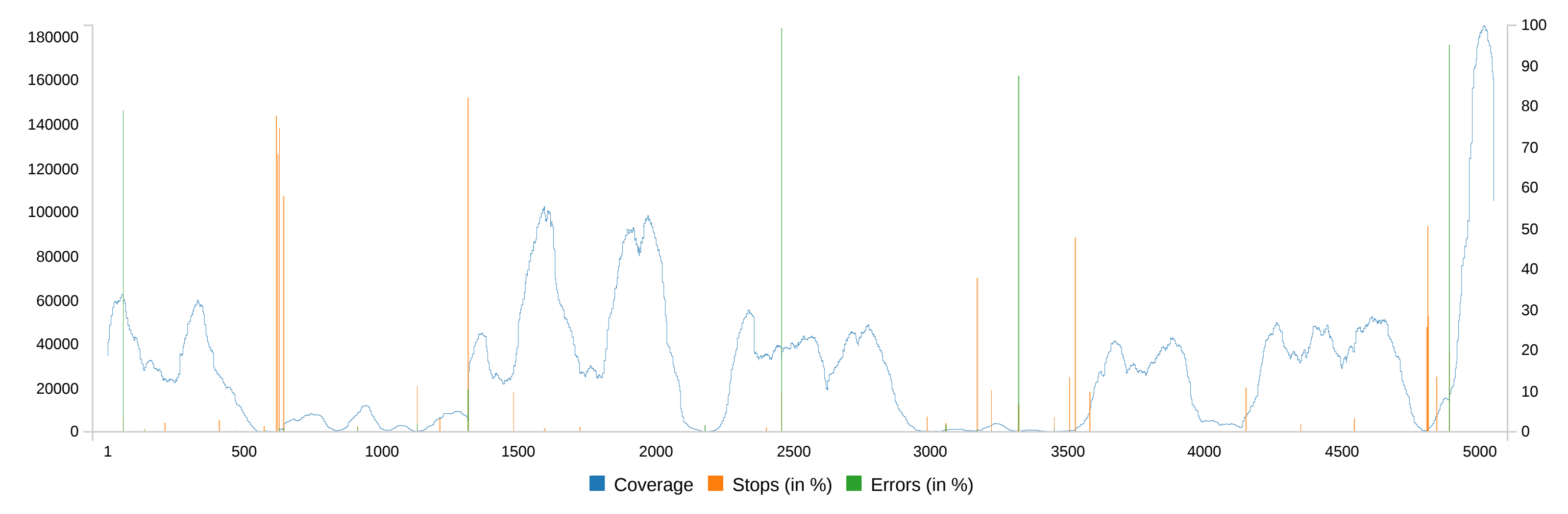

28S rRNA (Lib 4 m^1A-T)

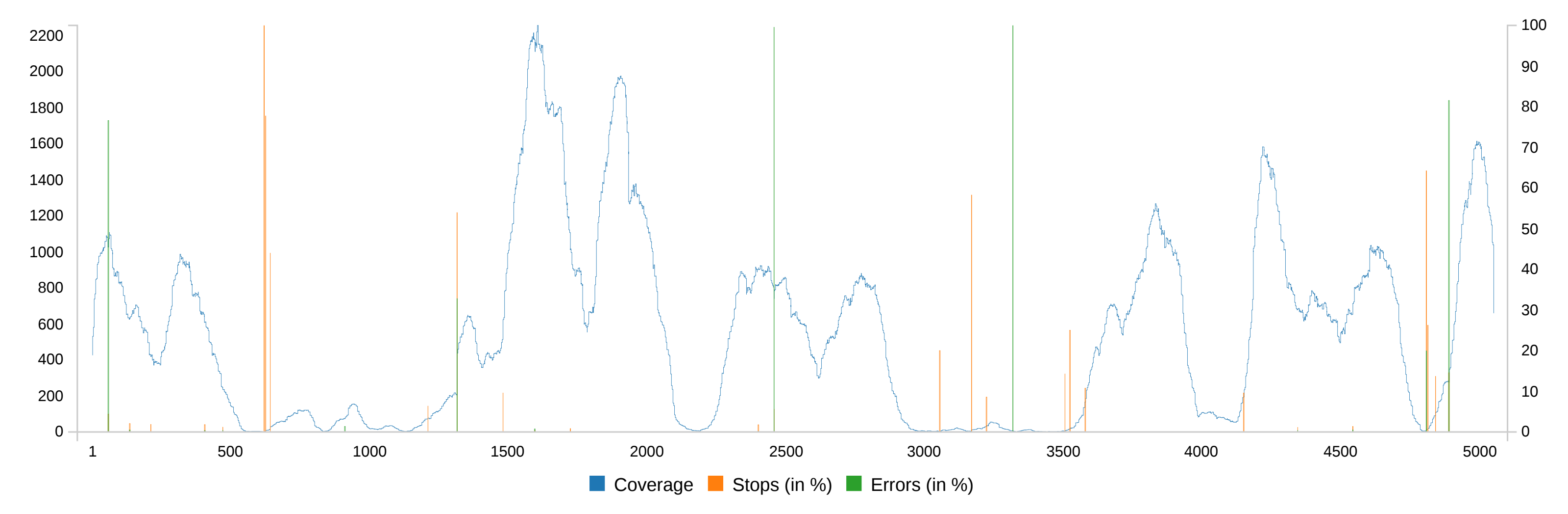

28S rRNA (Lib 3 m^6A)

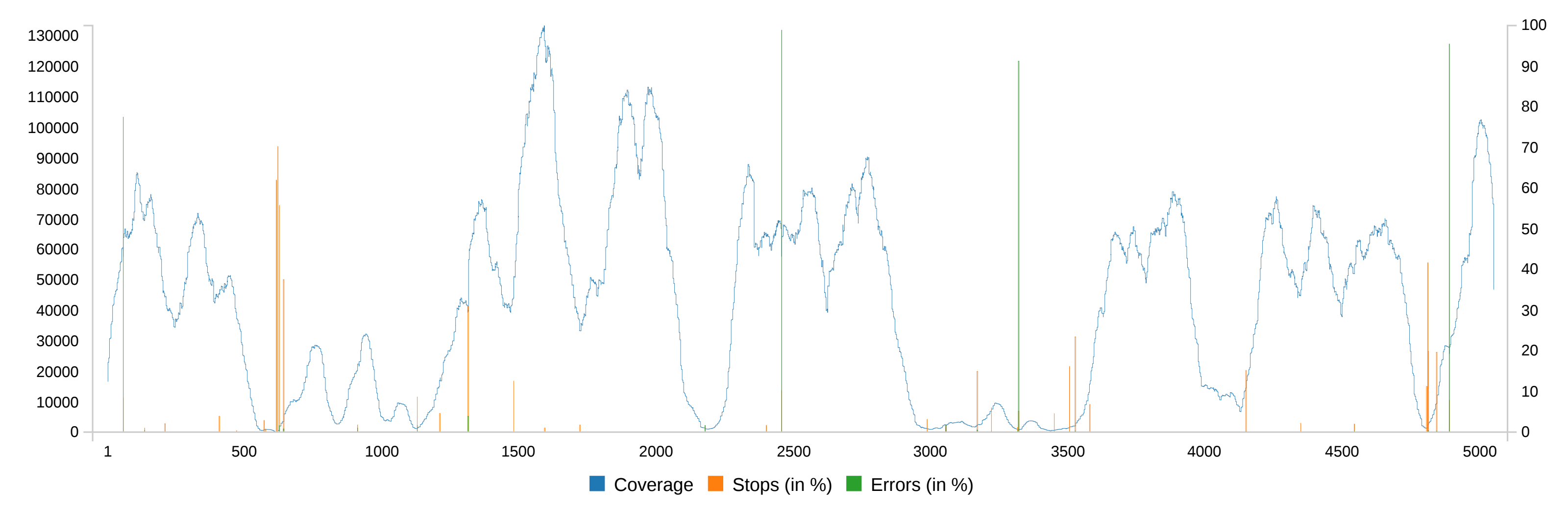

28S rRNA (Lib 4 m^6A-T)

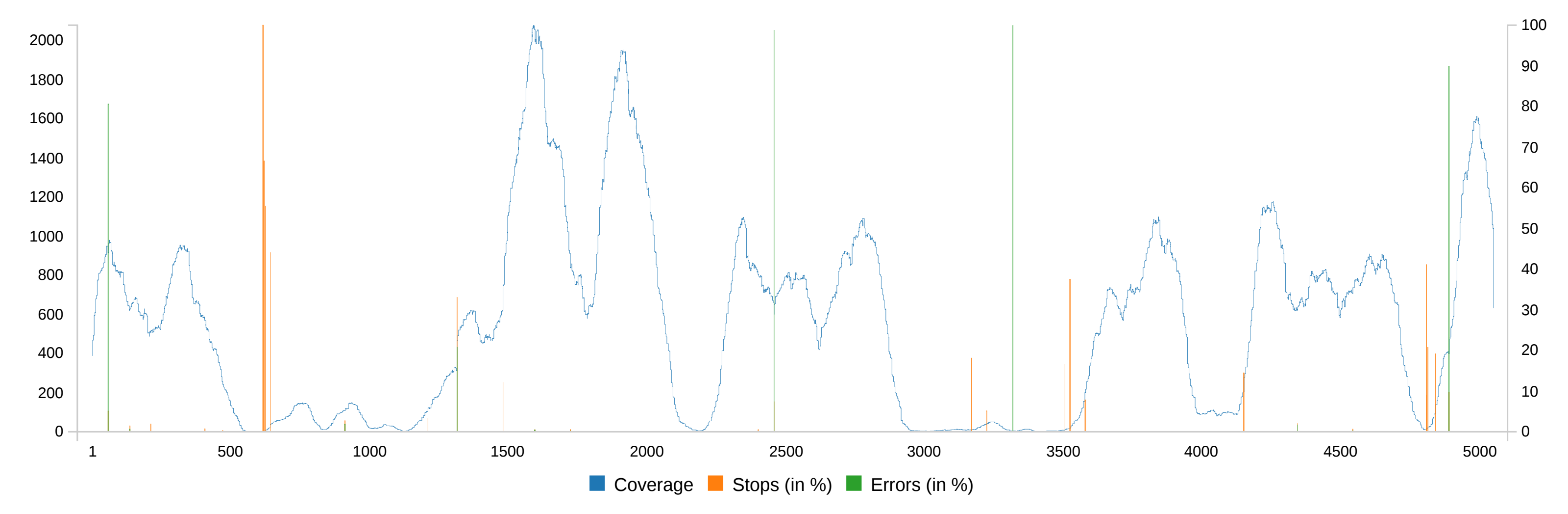

7SL RNA

- Stops (coverage drops)
- Errors (base mismatch)
- Pattern: "gttc.A..c"

	Lib 1 m¹A	Lib 2 m¹A	Lib 3 m¹A	Lib 4 m¹A-T	Lib 1 m⁶A	Lib 2 m⁶A	Lib 3 m⁶A	Lib 4 m⁶A-T	p-value
3 (C)	19%<br>0% (G: 0%)<br>(4458)	15%<br>0% (A: 0%)<br>(2150)	14%<br>0% (T: 0%)<br>(8058)	6%<br>0% (G: 0%)<br>(2939)	63%<br>0% (A: 0%)<br>(902)	35%<br>0% (A: 0%)<br>(414)	18%<br>0% (A: 0%)<br>(2131)	5%<br>0% (A: 0%)<br>(1038)	0.24<br>0.04
20 (G)	0%<br>0% (T: 0%)<br>(9187)	0%<br>0% (T: 0%)<br>(4354)	0%<br>0% (A: 0%)<br>(16350)	1%<br>0% (A: 0%)<br>(6203)	0%<br>0% (T: 0%)<br>(6837)	0%<br>0% (T: 0%)<br>(1684)	19%<br>0% (T: 0%)<br>(4973)	45%<br>0% (T: 0%)<br>(1440)	0.19<br>0.05
52 (C)	2%<br>29% (T: 29%)<br>(9640)	1%<br>27% (T: 27%)<br>(4829)	1%<br>41% (T: 40%)<br>(23970)	2%<br>38% (T: 37%)<br>(9126)	1%<br>19% (T: 19%)<br>(6673)	4%<br>16% (T: 16%)<br>(1484)	1%<br>28% (T: 28%)<br>(9001)	1%<br>19% (T: 18%)<br>(3750)	0.89<br>0.02
53 (T)	9%<br>29% (G: 29%)<br>(9505)	12%<br>27% (G: 27%)<br>(4729)	15%<br>41% (G: 41%)<br>(23438)	21%<br>39% (G: 39%)<br>(8657)	13%<br>20% (G: 20%)<br>(6449)	15%<br>16% (G: 16%)<br>(1451)	17%<br>28% (G: 28%)<br>(8972)	20%<br>19% (G: 18%)<br>(3742)	0.50<br>0.02
69 (T)	1%<br>35% (C: 35%)<br>(9836)	2%<br>33% (C: 33%)<br>(5503)	1%<br>49% (C: 48%)<br>(30734)	3%<br>52% (C: 52%)<br>(13113)	2%<br>28% (C: 28%)<br>(6209)	4%<br>29% (C: 29%)<br>(1782)	4%<br>38% (C: 37%)<br>(11493)	7%<br>33% (C: 32%)<br>(5141)	0.05<br>0.10
87 (T)	5%<br>0% (C: 0%)<br>(13604)	5%<br>0% (G: 0%)<br>(8728)	7%<br>0% (C: 0%)<br>(45282)	10%<br>0% (C: 0%)<br>(20538)	3%<br>0% (C: 0%)<br>(11762)	3%<br>0% (C: 0%)<br>(3193)	2%<br>0% (C: 0%)<br>(16836)	1%<br>0% (C: 0%)<br>(6984)	0.01<br>0.87
214 (A)	1%<br>0% (T: 0%)<br>(16775)	3%<br>0% (T: 0%)<br>(14726)	0%<br>0% (G: 0%)<br>(70314)	0%<br>0% (G: 0%)<br>(39945)	2%<br>0% (G: 0%)<br>(11857)	1%<br>0% (A: 0%)<br>(2788)	1%<br>0% (T: 0%)<br>(31114)	2%<br>0% (T: 0%)<br>(17105)	0.74<br>0.26
275 (G)	0%<br>27% (A: 27%)<br>(16695)	0%<br>39% (A: 39%)<br>(51118)	0%<br>47% (A: 47%)<br>(60296)	0%<br>56% (A: 56%)<br>(23375)	0%<br>23% (A: 23%)<br>(14112)	0%<br>29% (A: 29%)<br>(4262)	0%<br>46% (A: 46%)<br>(28740)	0%<br>60% (A: 59%)<br>(11060)	----<br>0.79

Overall statistics

	Lib 1 m¹A	Lib 2 m¹A	Lib 3 m¹A	Lib 4 m¹A-T	Lib 1 m⁶A	Lib 2 m⁶A	Lib 3 m⁶A	Lib 4 m⁶A-T
Total reads	85117	89297	176590	76897	76102	19991	83810	34810
Average coverage	16933 (σ=6466)	19508 (σ=16026)	53738 (σ=21326)	26461 (σ=13295)	12215 (σ=4867)	3012 (σ=1101)	21757 (σ=9601)	10456 (σ=5307)
Average errors	100 (σ=549)	127 (σ=1207)	326 (σ=2021)	174 (σ=899)	40 (σ=242)	12 (σ=82)	127 (σ=839)	71 (σ=399)
Total errors	0.59%	0.65%	0.61%	0.66%	0.34%	0.4%	0.58%	0.68%
Total "A" errors	0.27%	0.46%	0.24%	0.27%	0.13%	0.21%	0.28%	0.32%

7SL RNA (Lib 1 m^1A)

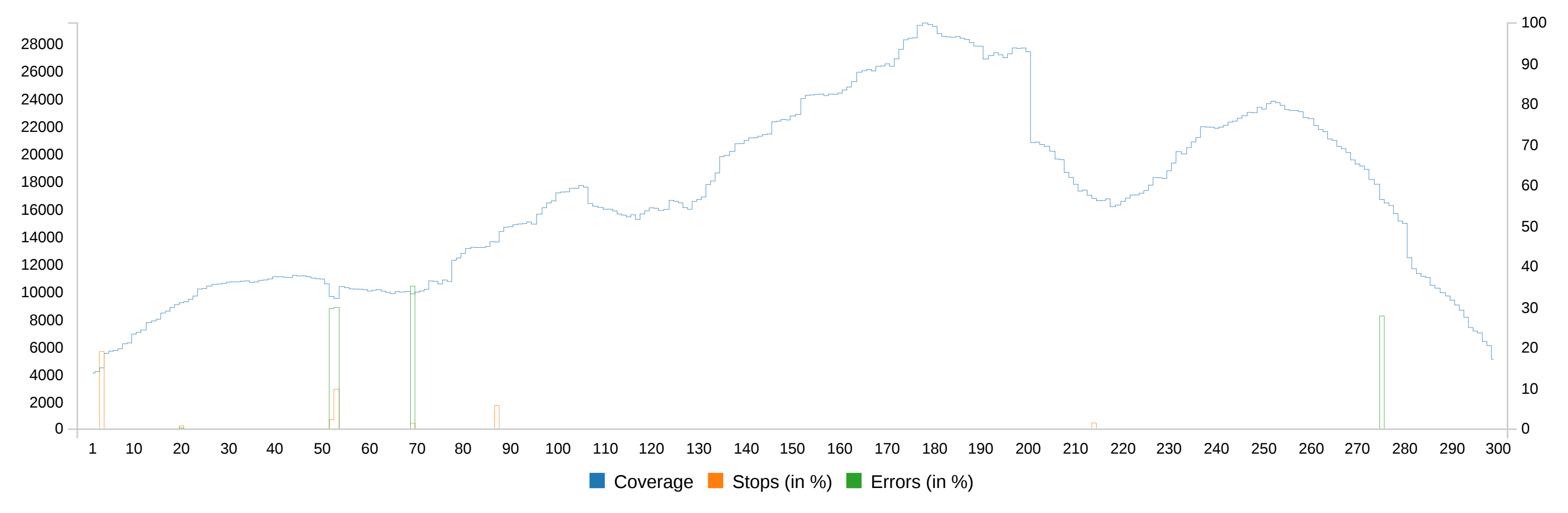

7SL RNA (Lib 2 m^1A)

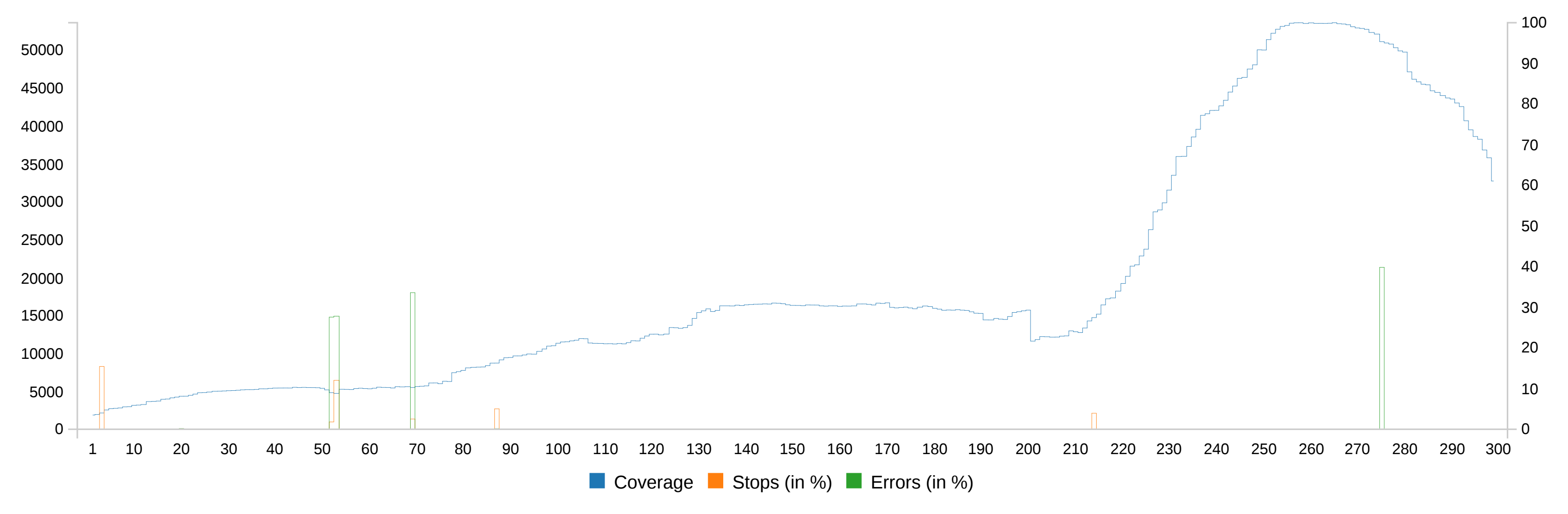

7SL RNA (Lib 3 m^1A)

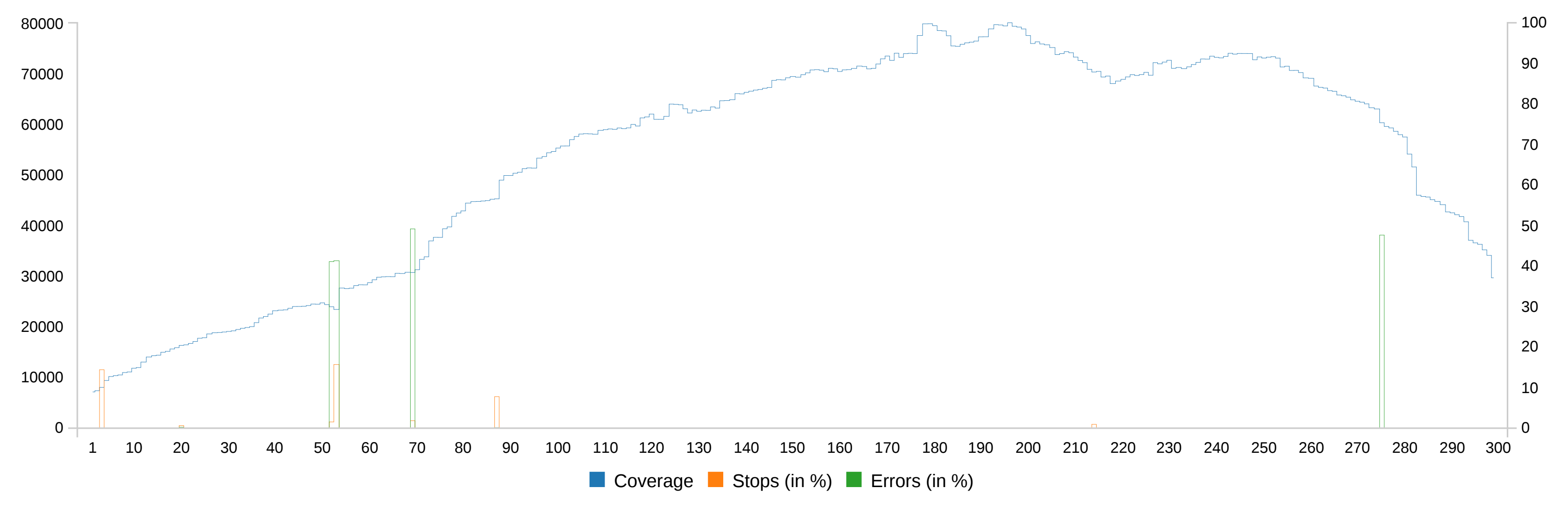

7SL RNA (Lib 4 m^1A-T)

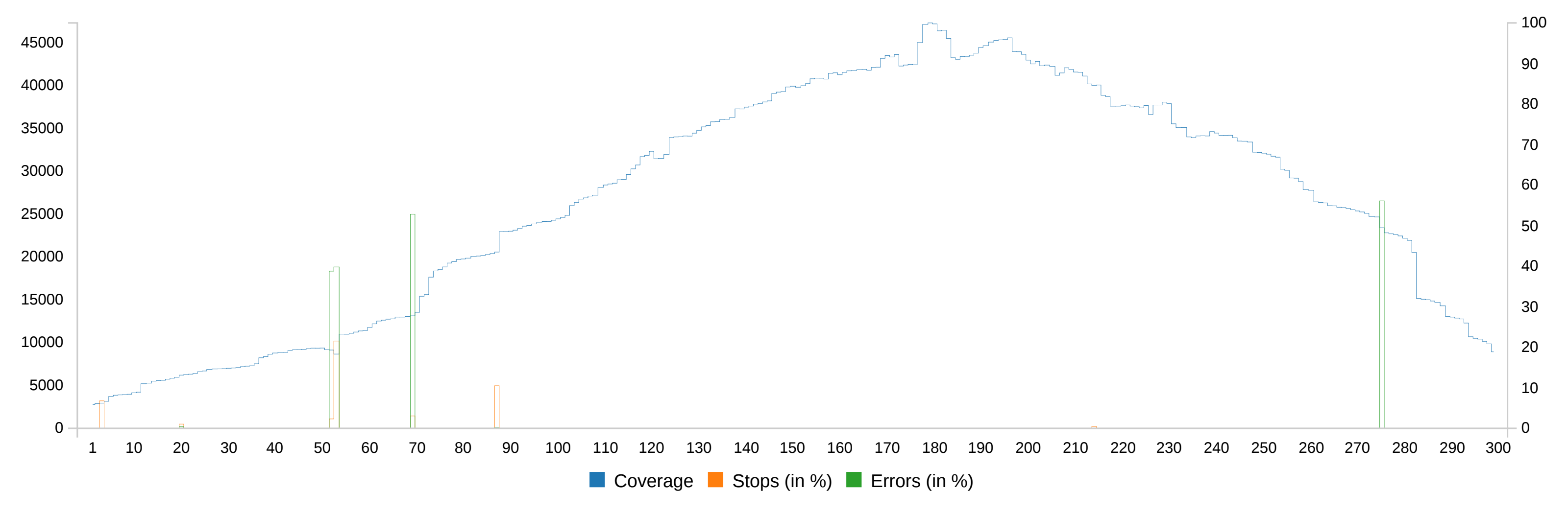

7SL RNA (Lib 1 m^6A)

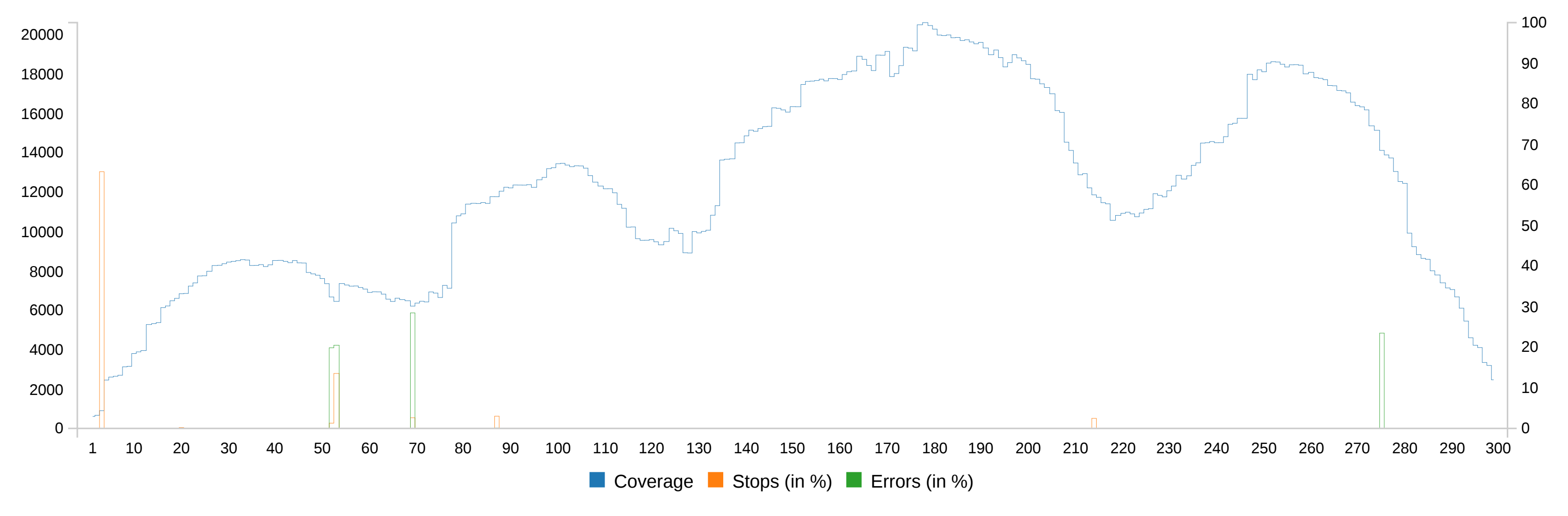

7SL RNA (Lib 2 m^6A)

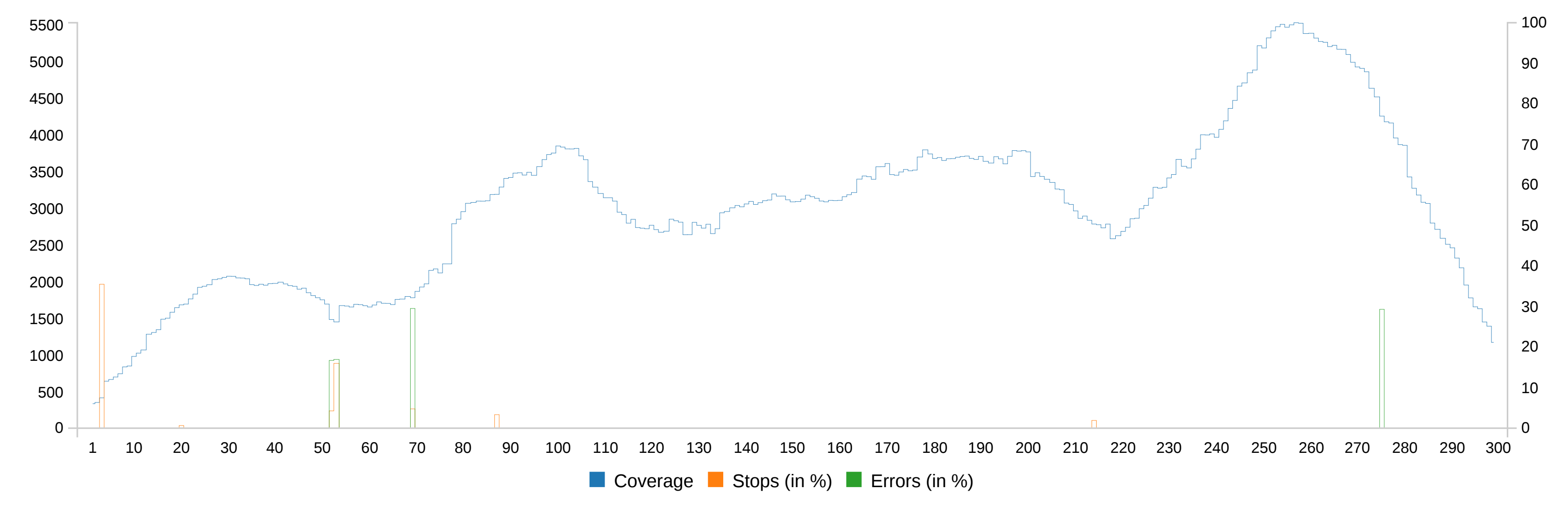

7SL RNA (Lib 3 m^6A)

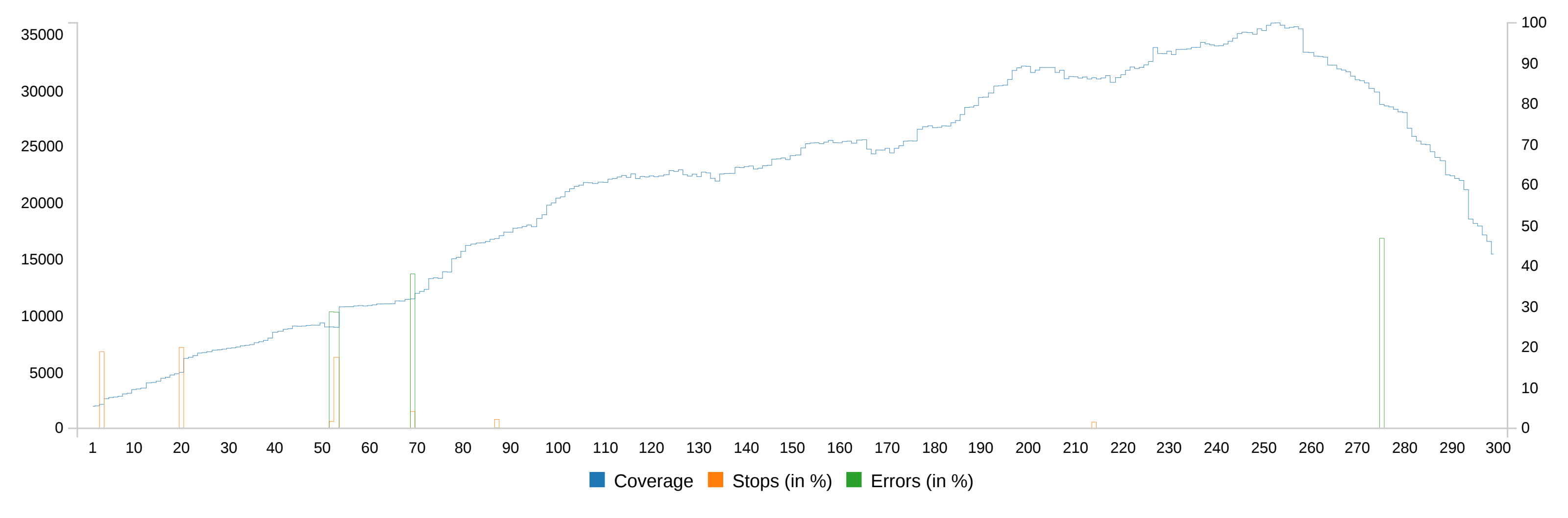

7SL RNA (Lib 4 m^6A-T)

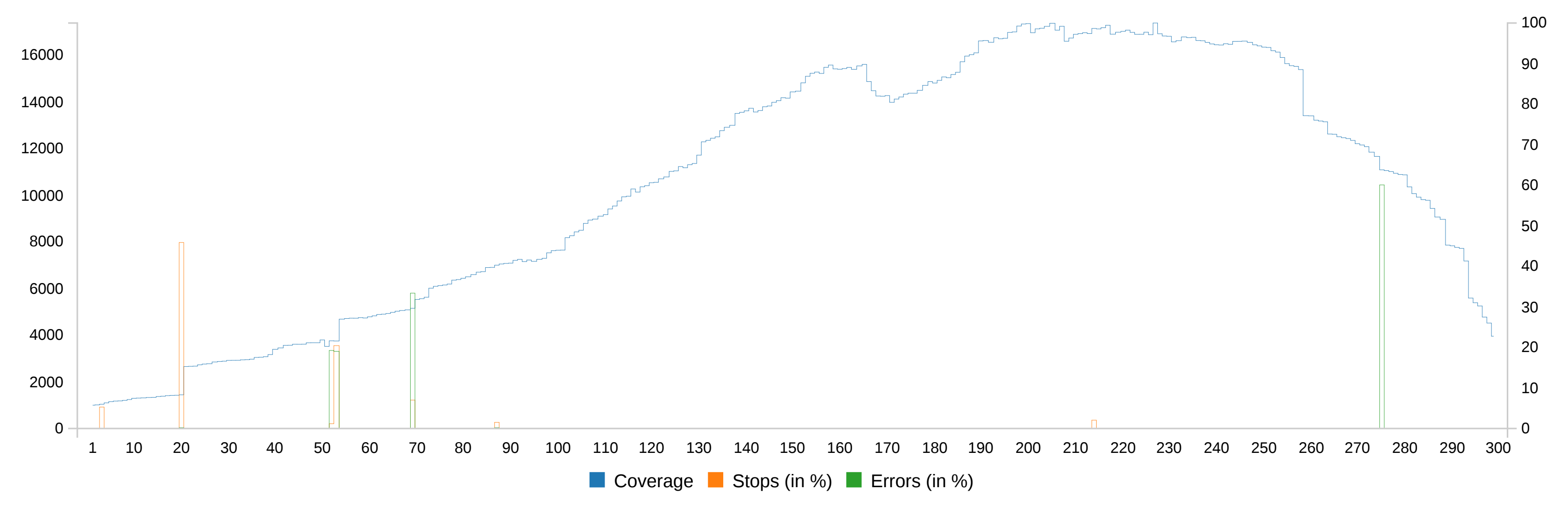

HIV NL43

- Stops (coverage drops)
- Errors (base mismatch)
- Pattern: "gttc.A..c"

	Lib 1 m^{1}A	Lib 2 m^{1}A	Lib 3 m^{1}A	Lib 4 m^{1}A-T	Lib 1 m^{6}A	Lib 2 m^{6}A	Lib 3 m^{6}A	Lib 4 m^{6}A-T	p-value
54 (T)	20%	10%	43%	10%	3%	9%	24%	8%	0.31
	0% (A: 0%)	0% (A: 0%)	0% (A: 0%)	0% (A: 0%)	0% (A: 0%)	0% (A: 0%)	0% (A: 0%)	0% (A: 0%)	----
	(88)	(51)	(101)	(50)	(86)	(30)	(79)	(32)	
109 (T)	0%	0%	0%	0%	0%	0%	0%	0%	----
	0% (A: 0%)	0% (A: 0%)	0% (A: 0%)	0% (A: 0%)	0% (A: 0%)	0% (A: 0%)	25% (A: 25%)	25% (A: 25%)	0.13
	(1)	(3)	(15)	(10)	(7)	(2)	(4)	(4)	
130 (G)	0%	0%	0%	0%	0%	0%	0%	0%	----
	0% (A: 0%)	0% (A: 0%)	0% (A: 0%)	0% (A: 0%)	0% (A: 0%)	0% (A: 0%)	0% (A: 0%)	0% (A: 0%)	----
	(0)	(0)	(4)	(1)	(3)	(0)	(1)	(1)	
208 (A)	0%	0%	0%	0%	0%	0%	0%	0%	----
	0% (A: 0%)	0% (A: 0%)	0% (A: 0%)	0% (A: 0%)	0% (A: 0%)	0% (A: 0%)	0% (A: 0%)	0% (A: 0%)	----
	(2)	(0)	(1)	(0)	(1)	(0)	(0)	(0)	
242 (G)	0%	0%	0%	0%	0%	0%	0%	0%	----
	0% (A: 0%)	0% (A: 0%)	0% (A: 0%)	0% (A: 0%)	0% (A: 0%)	0% (A: 0%)	0% (A: 0%)	0% (A: 0%)	----
	(2)	(0)	(0)	(0)	(2)	(0)	(0)	(0)	
339 (G)	0%	0%	0%	0%	0%	0%	0%	0%	----
	0% (A: 0%)	0% (A: 0%)	0% (A: 0%)	0% (A: 0%)	0% (A: 0%)	0% (A: 0%)	0% (A: 0%)	0% (A: 0%)	----
	(0)	(0)	(0)	(0)	(0)	(0)	(4)	(1)	
350 (G)	5%	0%	0%	0%	0%	0%	0%	0%	0.36
	0% (A: 0%)	0% (A: 0%)	0% (A: 0%)	0% (A: 0%)	0% (A: 0%)	0% (A: 0%)	0% (A: 0%)	0% (A: 0%)	----
	(18)	(1)	(7)	(1)	(7)	(6)	(9)	(3)	
374 (A)	6%	0%	10%	0%	0%	0%	0%	0%	0.15
	0% (A: 0%)	0% (A: 0%)	0% (A: 0%)	0% (A: 0%)	0% (A: 0%)	0% (A: 0%)	0% (A: 0%)	0% (A: 0%)	----
	(16)	(2)	(9)	(2)	(11)	(6)	(6)	(3)	
377 (G)	0%	0%	0%	0%	0%	0%	0%	0%	----
	0% (A: 0%)	0% (A: 0%)	0% (A: 0%)	0% (A: 0%)	0% (A: 0%)	0% (A: 0%)	0% (A: 0%)	0% (A: 0%)	----
	(14)	(2)	(10)	(2)	(12)	(6)	(5)	(2)	
399 (G)	0%	0%	0%	0%	0%	0%	0%	0%	----
	0% (A: 0%)	0% (A: 0%)	0% (A: 0%)	0% (A: 0%)	0% (A: 0%)	0% (A: 0%)	0% (A: 0%)	0% (A: 0%)	----
	(69)	(13)	(34)	(9)	(122)	(18)	(35)	(1)	
453 (T)	65%	72%	55%	69%	45%	40%	46%	42%	0.00
	2% (G: 2%)	6% (G: 6%)	1% (G: 1%)	5% (G: 5%)	1% (G: 1%)	2% (G: 2%)	0% (C: 0%)	2% (C: 2%)	0.09
	(189)	(266)	(459)	(88)	(85)	(42)	(112)	(42)	
454 (G)	4%	4%	40%	71%	1%	4%	6%	7%	0.17
	0% (A: 0%)	0% (A: 0%)	0% (A: 0%)	0% (A: 0%)	0% (A: 0%)	0% (A: 0%)	0% (C: 0%)	1% (C: 1%)	0.26
	(542)	(950)	(1031)	(282)	(157)	(71)	(208)	(71)	
455 (G)	4%	3%	27%	39%	1%	3%	47%	71%	0.54
	0% (C: 0%)	0% (A: 0%)	0% (C: 0%)	0% (C: 0%)	0% (T: 0%)	0% (A: 0%)	0% (A: 0%)	0% (A: 0%)	0.98
	(569)	(993)	(1736)	(978)	(160)	(74)	(223)	(77)	
463 (T)	7%	5%	4%	4%	17%	16%	10%	8%	0.02
	0% (G: 0%)	0% (C: 0%)	0% (C: 0%)	0% (C: 0%)	0% (A: 0%)	0% (A: 0%)	0% (A: 0%)	0% (A: 0%)	0.00
	(686)	(1192)	(3215)	(2362)	(260)	(100)	(505)	(318)	
468 (A)	4%	4%	1%	0%	13%	14%	7%	7%	0.01
	0% (A: 0%)	0% (A: 0%)	0% (G: 0%)	0% (G: 0%)	0% (A: 0%)	0% (A: 0%)	0% (A: 0%)	0% (A: 0%)	0.13
	(763)	(1297)	(3471)	(2537)	(340)	(125)	(610)	(395)	
471 (C)	6%	3%	2%	1%	9%	4%	13%	15%	0.03
	0% (T: 0%)	0% (A: 0%)	2% (G: 2%)	3% (G: 2%)	0% (A: 0%)	0% (A: 0%)	0% (T: 0%)	0% (T: 0%)	0.11
	(832)	(1402)	(3646)	(2643)	(408)	(156)	(755)	(499)	
564 (C)	12%	15%	1%	0%	40%	11%	1%	1%	0.56
	0% (T: 0%)	0% (A: 0%)	0% (T: 0%)	0% (A: 0%)	0% (A: 0%)	0% (A: 0%)	0% (A: 0%)	0% (A: 0%)	0.63
	(1854)	(2521)	(12793)	(8426)	(371)	(439)	(5926)	(4252)	
615 (A)	6%	3%	2%	0%	10%	10%	7%	7%	0.01
	0% (A: 0%)	0% (G: 0%)	0% (G: 0%)	0% (G: 0%)	0% (A: 0%)	0% (A: 0%)	0% (C: 0%)	0% (C: 0%)	0.74
	(1505)	(2884)	(13661)	(8485)	(420)	(352)	(5388)	(3841)	
646 (A)	6%	8%	11%	15%	2%	0%	5%	6%	0.03
	0% (G: 0%)	0% (G: 0%)	0% (C: 0%)	0% (C: 0%)	0% (A: 0%)	0% (G: 0%)	0% (C: 0%)	0% (C: 0%)	0.30
	(2351)	(2620)	(16571)	(9256)	(755)	(712)	(5962)	(3726)	
1059 (A)	3%	6%	0%	0%	6%	11%	0%	0%	0.54
	0% (A: 0%)	0% (A: 0%)	0% (A: 0%)	0% (A: 0%)	0% (A: 0%)	0% (A: 0%)	0% (A: 0%)	0% (A: 0%)	----
	(246)	(2901)	(2598)	(1066)	(32)	(213)	(1741)	(1391)	
1212 (A)	2%	1%	6%	9%	0%	1%	0%	0%	0.07
	0% (G: 0%)	0% (C: 0%)	0% (A: 0%)	0% (A: 0%)	0% (A: 0%)	0% (A: 0%)	0% (T: 0%)	0% (A: 0%)	0.45
	(311)	(1032)	(3148)	(2035)	(130)	(183)	(1377)	(838)	
1308 (A)	4%	3%	26%	36%	0%	4%	1%	0%	0.10
	0% (A: 0%)	0% (A: 0%)	0% (G: 0%)	0% (G: 0%)	0% (A: 0%)	0% (A: 0%)	0% (G: 0%)	0% (G: 0%)	0.60
	(290)	(1164)	(1440)	(908)	(41)	(220)	(1516)	(1268)	
1393 (A)	7%	2%	0%	0%	7%	2%	2%	2%	0.60
	0% (A: 0%)	0% (G: 0%)	0% (A: 0%)	0% (A: 0%)	0% (A: 0%)	0% (A: 0%)	0% (A: 0%)	0% (A: 0%)	0.36
	(615)	(1251)	(8891)	(7690)	(111)	(210)	(2702)	(2264)	
1645 (A)	4%	2%	12%	20%	2%	2%	1%	1%	0.08
	0% (A: 0%)	0% (G: 0%)	0% (A: 0%)	0% (A: 0%)	0% (A: 0%)	0% (G: 0%)	0% (A: 0%)	0% (A: 0%)	0.68
	(465)	(993)	(659)	(339)	(89)	(254)	(395)	(287)	
1648 (C)	3%	1%	10%	13%	2%	2%	2%	0%	0.09
	0% (T: 0%)	0% (A: 0%)	0% (A: 0%)	0% (A: 0%)	0% (A: 0%)	0% (A: 0%)	0% (A: 0%)	0% (A: 0%)	0.36
	(479)	(971)	(877)	(551)	(92)	(247)	(413)	(302)	
1728 (A)	5%	11%	3%	2%	2%	11%	5%	6%	0.77
	0% (A: 0%)	1% (T: 1%)	0% (T: 0%)	1% (T: 1%)	0% (A: 0%)	0% (A: 0%)	4% (T: 4%)	7% (T: 6%)	0.25
	(581)	(671)	(1070)	(700)	(33)	(93)	(434)	(285)	
1916 (T)	9%	4%	11%	14%	0%	3%	1%	0%	0.01
	0% (A: 0%)	0% (A: 0%)	0% (C: 0%)	0% (C: 0%)	0% (A: 0%)	0% (A: 0%)	0% (A: 0%)	0% (A: 0%)	0.14
	(266)	(776)	(1677)	(1281)	(54)	(185)	(598)	(398)	
1996 (A)	4%	4%	0%	0%	15%	9%	4%	5%	0.07
	0% (C: 0%)	0% (C: 0%)	0% (C: 0%)	0% (C: 0%)	0% (A: 0%)	0% (A: 0%)	0% (C: 0%)	0% (C: 0%)	0.52
	(715)	(950)	(3290)	(2533)	(207)	(106)	(857)	(649)	
2091 (A)	14%	42%	2%	0%	64%	65%	2%	0%	0.41
	0% (G: 0%)	0% (G: 0%)	2% (G: 2%)	3% (G: 3%)	1% (G: 1%)	0% (A: 0%)	0% (G: 0%)	0% (G: 0%)	0.22
	(668)	(404)	(1245)	(579)	(186)	(72)	(591)	(325)	
2112 (C)	12%	1%	40%	48%	6%	2%	10%	0%	0.12
	0% (A: 0%)	0% (T: 0%)	0% (T: 0%)	0% (G: 0%)	0% (T: 0%)	0% (A: 0%)	0% (A: 0%)	0% (A: 0%)	0.52
	(844)	(775)	(1235)	(540)	(914)	(269)	(548)	(287)	
2138 (A)	8%	5%	24%	46%	8%	6%	8%	7%	0.21
	0% (A: 0%)	0% (C: 0%)	0% (C: 0%)	0% (C: 0%)	0% (C: 0%)	0% (A: 0%)	0% (C: 0%)	0% (C: 0%)	0.41
	(1136)	(855)	(2057)	(696)	(1359)	(286)	(938)	(595)	
2292 (A)	6%	2%	0%	0%	3%	8%	15%	22%	0.06
	0% (A: 0%)	0% (A: 0%)	0% (A: 0%)	0% (A: 0%)	0% (A: 0%)	0% (A: 0%)	0% (G: 0%)	0% (G: 0%)	0.16
	(259)	(369)	(1784)	(1387)	(125)	(56)	(596)	(349)	
2434 (A)	0%	1%	0%	0%	5%	4%	11%	13%	0.01
	0% (A: 0%)	0% (A: 0%)	0% (C: 0%)	0% (A: 0%)	0% (A: 0%)	0% (A: 0%)	0% (A: 0%)	0% (A: 0%)	0.36
	(221)	(401)	(924)	(690)	(17)	(44)	(507)	(412)	
2583 (T)	11%	5%	8%	1%	0%	0%	1%	0%	0.03
	0% (A: 0%)	0% (A: 0%)	0% (A: 0%)	0% (A: 0%)	0% (A: 0%)	0% (A: 0%)	0% (A: 0%)	0% (A: 0%)	----
	(135)	(173)	(299)	(58)	(13)	(14)	(145)	(60)	
2685 (A)	2%	0%	0%	0%	0%	0%	0%	0%	0.28
	0% (A: 0%)	0% (A: 0%)	0% (A: 0%)	0% (A: 0%)	0% (A: 0%)	0% (A: 0%)	0% (A: 0%)	0% (A: 0%)	----
	(35)	(241)	(59)	(30)	(4)	(24)	(141)	(123)	
2692 (C)	9%	7%	66%	77%	20%	13%	0%	0%	0.15
	0% (A: 0%)	0% (A: 0%)	0% (A: 0%)	0% (A: 0%)	0% (A: 0%)	0% (A: 0%)	0% (A: 0%)	0% (A: 0%)	0.13
	(37)	(194)	(49)	(28)	(4)	(19)	(133)	(121)	
2802 (C)	2%	4%	11%	55%	14%	0%	74%	81%	0.36
	0% (A: 0%)	0% (A: 0%)	0% (A: 0%)	0% (A: 0%)	0% (A: 0%)	0% (A: 0%)	2% (G: 2%)	3% (G: 3%)	0.15
	(74)	(68)	(72)	(4)	(6)	(8)	(50)	(32)	
2822 (A)	11%	4%	2%	0%	16%	15%	1%	0%	0.52
	0% (A: 0%)	0% (A: 0%)	0% (T: 0%)	0% (A: 0%)	0% (A: 0%)	0% (A: 0%)	0% (A: 0%)	0% (A: 0%)	0.36
	(117)	(89)	(103)	(10)	(15)	(11)	(266)	(237)	
2836 (A)	0%	0%	0%	0%	0%	0%	0%	0%	0.36
	0% (A: 0%)	0% (A: 0%)	0% (A: 0%)	0% (A: 0%)	0% (A: 0%)	0% (A: 0%)	0% (A: 0%)	0% (A: 0%)	----
	(274)	(148)	(189)	(9)	(48)	(24)	(345)	(260)	
2857 (A)	0%	3%	0%	0%	2%	0%	1%	1%	0.80
	0% (A: 0%)	0% (A: 0%)	0% (A: 0%)	0% (A: 0%)	0% (A: 0%)	0% (A: 0%)	0% (A: 0%)	0% (A: 0%)	----
	(310)	(125)	(192)	(12)	(46)	(20)	(379)	(293)	
2867 (A)	3%	3%	29%	68%	2%	0%	1%	0%	0.15
	0% (A: 0%)	0% (A: 0%)	0% (A: 0%)	0% (A: 0%)	0% (A: 0%)	0% (A: 0%)	0% (A: 0%)	0% (A: 0%)	----
	(257)	(107)	(208)	(38)	(44)	(14)	(385)	(301)	
2895 (T)	0%	2%	3%	0%	0%	0%	0%	0%	0.06
	0% (A: 0%)	0% (A: 0%)	0% (A: 0%)	0% (A: 0%)	7% (A: 7%)	0% (A: 0%)	0% (A: 0%)	0% (A: 0%)	0.36
	(223)	(94)	(324)	(172)	(27)	(13)	(422)	(330)	
2912 (T)	11%	3%	5%	0%	0%	0%	0%	0%	0.08
	0% (A: 0%)	0% (A: 0%)	0% (A: 0%)	0% (A: 0%)	0% (A: 0%)	0% (A: 0%)	0% (A: 0%)	0% (A: 0%)	----
	(161)	(92)	(326)	(186)	(11)	(9)	(403)	(319)	
2957 (C)	17%	5%	47%	63%	12%	10%	6%	3%	0.10
	0% (A: 0%)	0% (A: 0%)	0% (G: 0%)	1% (G: 1%)	0% (A: 0%)	0% (A: 0%)	0% (A: 0%)	0% (A: 0%)	0.19
	(199)	(129)	(334)	(137)	(23)	(11)	(530)	(431)	
3030 (C)	3%	1%	21%	28%	1%	0%	1%	0%	0.10
	0% (A: 0%)	0% (A: 0%)	0% (T: 0%)	0% (A: 0%)	0% (A: 0%)	0% (A: 0%)	0% (A: 0%)	0% (A: 0%)	0.49
	(437)	(203)	(1098)	(715)	(77)	(27)	(579)	(430)	
3096 (T)	23%	9%	47%	45%	38%	10%	9%	6%	0.24
	0% (A: 0%)	0% (A: 0%)	0% (A: 0%)	0% (A: 0%)	0% (A: 0%)	0% (A: 0%)	0% (A: 0%)	0% (A: 0%)	----
	(194)	(50)	(691)	(605)	(8)	(13)	(239)	(158)	
3213 (C)	14%	5%	9%	10%	6%	0%	2%	1%	0.03
	0% (A: 0%)	0% (A: 0%)	0% (T: 0%)	0% (A: 0%)	0% (A: 0%)	0% (A: 0%)	0% (A: 0%)	0% (A: 0%)	0.36
	(441)	(99)	(720)	(222)	(86)	(27)	(776)	(483)	
3243 (T)	4%	1%	42%	57%	0%	0%	2%	0%	0.11
	0% (A: 0%)	0% (A: 0%)	0% (A: 0%)	0% (A: 0%)	0% (A: 0%)	0% (A: 0%)	0% (A: 0%)	0% (A: 0%)	0.18
	(398)	(73)	(750)	(327)	(87)	(18)	(611)	(392)	
3306 (A)	0%	0%	0%	0%	1%	0%	32%	44%	0.14
	0% (A: 0%)	1% (G: 1%)	0% (G: 0%)	0% (G: 0%)	0% (A: 0%)	0% (A: 0%)	0% (A: 0%)	0% (G: 0%)	0.39
	(761)	(201)	(1847)	(961)	(175)	(29)	(1030)	(625)	
3328 (T)	7%	0%	11%	13%	0%	0%	0%	0%	0.07
	0% (A: 0%)	0% (A: 0%)	0% (A: 0%)	0% (A: 0%)	0% (A: 0%)	0% (A: 0%)	0% (A: 0%)	0% (A: 0%)	0.49
	(292)	(138)	(1250)	(829)	(88)	(12)	(1199)	(981)	
3588 (A)	5%	13%	0%	0%	0%	0%	[illegible]	[illegible]	0.97
	0% (A: 0%)	0% (A: 0%)	0% (A: 0%)	0% (A: 0%)	0% (A: 0%)	0% (A: 0%)	0% (A: 0%)	0% (A: 0%)	----
	(21)	(25)	(54)	(30)	(0)	(2)	(51)	(43)	
3613 (C)	12%	9%	12%	3%	0%	0%	41%	42%	0.39
	0% (A: 0%)	0% (A: 0%)	0% (A: 0%)	0% (A: 0%)	0% (A: 0%)	0% (A: 0%)	0% (A: 0%)	0% (A: 0%)	----
	(69)	(57)	(92)	(32)	(6)	(2)	(60)	(40)	
3651 (T)	3%	2%	86%	99%	0%	0%	0%	0%	0.12
	0% (A: 0%)	0% (A: 0%)	0% (A: 0%)	0% (A: 0%)	0% (A: 0%)	0% (A: 0%)	0% (A: 0%)	0% (A: 0%)	----
	(113)	(71)	(112)	(29)	(10)	(9)	(140)	(91)	
3668 (A)	0%	0%	0%	0%	25%	0%	0%	0%	0.45
	0% (A: 0%)	0% (A: 0%)	0% (A: 0%)	0% (A: 0%)	0% (A: 0%)	0% (A: 0%)	0% (A: 0%)	0% (A: 0%)	----
	(81)	(49)	(558)	(498)	(6)	(6)	(122)	(85)	
3717 (A)	5%	0%	27%	30%	0%	0%	0%	0%	0.09
	0% (A: 0%)	0% (A: 0%)	0% (A: 0%)	0% (A: 0%)	0% (A: 0%)	0% (A: 0%)	0% (A: 0%)	0% (A: 0%)	0.14
	(100)	(21)	(1000)	(853)	(8)	(1)	(134)	(66)	
3733 (A)	6%	2%	6%	6%	25%	12%	16%	20%	0.00
	0% (A: 0%)	0% (A: 0%)	0% (A: 0%)	0% (A: 0%)	0% (A: 0%)	0% (A: 0%)	0% (A: 0%)	0% (A: 0%)	0.72
	(362)	(36)	(1851)	(1478)	(95)	(7)	(244)	(121)	
3734 (G)	0%	5%	0%	0%	3%	11%	7%	13%	0.04
	0% (A: 0%)	0% (A: 0%)	0% (A: 0%)	0% (A: 0%)	0% (A: 0%)	0% (A: 0%)	0% (A: 0%)	0% (A: 0%)	0.14
	(383)	(34)	(1975)	(1573)	(127)	(8)	(289)	(152)	
3740 (A)	0%	0%	0%	0%	6%	15%	10%	0%	0.01
	0% (A: 0%)	0% (A: 0%)	0% (A: 0%)	0% (A: 0%)	0% (A: 0%)	0% (A: 0%)	0% (A: 0%)	0% (A: 0%)	0.36
	(493)	(49)	(2159)	(1638)	(185)	(11)	(313)	(198)	
3767 (G)	0%	0%	0%	0%	0%	4%	6%	10%	0.07
	0% (A: 0%)	0% (A: 0%)	0% (A: 0%)	0% (A: 0%)	0% (A: 0%)	0% (A: 0%)	0% (A: 0%)	0% (A: 0%)	0.86
	(576)	(76)	(1948)	(1364)	(287)	(24)	(546)	(341)	
3848 (C)	0%	0%	0%	0%	0%	0%	0%	0%	0.13
	0% (A: 0%)	0% (A: 0%)	1% (A: 1%)	1% (A: 1%)	0% (A: 0%)	0% (A: 0%)	0% (A: 0%)	0% (A: 0%)	0.32
	(280)	(41)	(1135)	(730)	(33)	(9)	(496)	(365)	
3972 (C)	9%	1%	10%	7%	2%	0%	3%	0%	0.03
	0% (A: 0%)	0% (A: 0%)	0% (A: 0%)	0% (A: 0%)	0% (A: 0%)	0% (A: 0%)	0% (A: 0%)	0% (A: 0%)	----
	(311)	(66)	(283)	(48)	(95)	(11)	(166)	(87)	
3988 (A)	2%	0%	0%	0%	4%	0%	16%	22%	0.10
	0% (A: 0%)	0% (A: 0%)	0% (A: 0%)	0% (A: 0%)	0% (A: 0%)	0% (A: 0%)	0% (A: 0%)	0% (A: 0%)	----
	(227)	(28)	(249)	(53)	(24)	(0)	(173)	(112)	
4134 (G)	9%	0%	0%	0%	0%	0%	0%	3%	0.65
	0% (A: 0%)	0% (A: 0%)	0% (A: 0%)	0% (A: 0%)	0% (A: 0%)	0% (A: 0%)	0% (A: 0%)	2% (G: 2%)	0.97
	(98)	(29)	(142)	(42)	(21)	(6)	(101)	(67)	
4231 (T)	5%	0%	1%	2%	0%	14%	0%	0%	0.76
	0% (A: 0%)	0% (A: 0%)	0% (A: 0%)	0% (A: 0%)	0% (A: 0%)	0% (A: 0%)	0% (A: 0%)	0% (A: 0%)	----
	(118)	(53)	(126)	(47)	(6)	(6)	(61)	(30)	
4243 (T)	8%	6%	15%	15%	10%	0%	5%	0%	0.07
	0% (A: 0%)	0% (A: 0%)	0% (A: 0%)	0% (A: 0%)	0% (A: 0%)	0% (A: 0%)	0% (A: 0%)	0% (A: 0%)	----
	(152)	(75)	(133)	(44)	(9)	(8)	(70)	(32)	
4272 (T)	5%	6%	30%	46%	13%	0%	7%	0%	0.16
	0% (A: 0%)	0% (A: 0%)	1% (A: 1%)	1% (A: 1%)	0% (A: 0%)	0% (A: 0%)	0% (A: 0%)	0% (A: 0%)	0.14
	(154)	(55)	(237)	(112)	(13)	(13)	(86)	(44)	
4311 (C)	1%	15%	8%	10%	0%	0%	1%	1%	0.02
	0% (A: 0%)	0% (A: 0%)	0% (T: 0%)	0% (T: 0%)	0% (A: 0%)	0% (A: 0%)	1% (A: 1%)	1% (A: 1%)	0.20
	(161)	(36)	(573)	(408)	(13)	(1)	(307)	(238)	
4351 (A)	2%	3%	12%	21%	2%	0%	0%	0%	0.10
	0% (A: 0%)	0% (A: 0%)	0% (G: 0%)	0% (G: 0%)	0% (A: 0%)	0% (A: 0%)	0% (A: 0%)	0% (A: 0%)	0.17
	(498)	(63)	(1009)	(497)	(93)	(7)	(726)	(568)	
4439 (A)	2%	10%	11%	16%	0%	0%	1%	0%	0.02
	0% (A: 0%)	0% (A: 0%)	0% (G: 0%)	0% (G: 0%)	0% (A: 0%)	0% (A: 0%)	1% (G: 1%)	1% (G: 1%)	0.64
	(306)	(22)	(791)	(509)	(18)	(3)	(1291)	(1158)	
4541 (A)	2%	1%	0%	0%	6%	0%	6%	10%	0.07
	0% (A: 0%)	0% (A: 0%)	0% (G: 0%)	0% (G: 0%)	0% (A: 0%)	0% (A: 0%)	0% (A: 0%)	0% (A: 0%)	0.16
	(284)	(65)	(912)	(485)	(41)	(5)	(353)	(190)	
4560 (A)	2%	1%	0%	0%	3%	0%	0%	0%	0.88
	0% (A: 0%)	0% (A: 0%)	0% (A: 0%)	0% (A: 0%)	0% (A: 0%)	0% (A: 0%)	0% (A: 0%)	0% (A: 0%)	----
	(349)	(75)	(763)	(397)	(51)	(6)	(357)	(204)	
4566 (G)	0%	0%	0%	0%	0%	0%	33%	46%	0.15
	0% (A: 0%)	0% (A: 0%)	0% (T: 0%)	0% (T: 0%)	0% (A: 0%)	0% (A: 0%)	0% (A: 0%)	0% (A: 0%)	0.17
	(371)	(70)	(558)	(266)	(56)	(6)	(337)	(195)	
4601 (T)	3%	4%	3%	2%	20%	16%	8%	10%	0.01
	0% (A: 0%)	0% (A: 0%)	0% (A: 0%)	0% (A: 0%)	0% (A: 0%)	0% (A: 0%)	0% (G: 0%)	0% (G: 0%)	0.14
	(382)	(83)	(995)	(460)	(182)	(15)	(874)	(614)	
4714 (T)	30%	14%	12%	5%	0%	0%	3%	0%	0.03
	0% (A: 0%)	0% (A: 0%)	0% (A: 0%)	0% (A: 0%)	0% (A: 0%)	0% (A: 0%)	0% (A: 0%)	0% (A: 0%)	----
	(50)	(24)	(210)	(157)	(5)	(2)	(167)	(143)	
4781 (T)	0%	0%	0%	0%	0%	0%	0%	0%	----
	2% (C: 2%)	6% (A: 6%)	3% (G: 3%)	33% (G: 33%)	0% (A: 0%)	0% (A: 0%)	0% (A: 0%)	0% (A: 0%)	0.17
	(37)	(15)	(31)	(3)	(3)	(1)	(15)	(4)	
4785 (A)	5%	5%	0%	0%	30%	0%	0%	0%	0.55
	0% (A: 0%)	5% (G: 5%)	0% (A: 0%)	0% (A: 0%)	0% (A: 0%)	0% (A: 0%)	0% (A: 0%)	0% (A: 0%)	0.36
	(20)	(17)	(18)	(3)	(9)	(0)	(9)	(3)	
4786 (A)	0%	5%	0%	0%	13%	0%	0%	0%	0.61
	0% (A: 0%)	0% (A: 0%)	0% (A: 0%)	0% (A: 0%)	0% (A: 0%)	0% (A: 0%)	0% (A: 0%)	0% (A: 0%)	----
	(20)	(18)	(17)	(3)	(13)	(0)	(9)	(3)	
4787 (A)	5%	5%	25%	0%	11%	100%	0%	0%	0.47
	0% (A: 0%)	0% (A: 0%)	0% (A: 0%)	0% (A: 0%)	0% (A: 0%)	0% (A: 0%)	0% (A: 0%)	0% (A: 0%)	----
	(18)	(19)	(15)	(3)	(15)	(0)	(8)	(3)	
4883 (A)	0%	0%	25%	0%	0%	0%	0%	0%	0.36
	0% (A: 0%)	0% (A: 0%)	0% (A: 0%)	0% (A: 0%)	0% (A: 0%)	0% (A: 0%)	0% (A: 0%)	0% (A: 0%)	----
	(4)	(5)	(6)	(3)	(0)	(0)	(12)	(9)	
4895 (T)	0%	0%	81%	95%	0%	100%	13%	0%	0.67
	0% (A: 0%)	0% (A: 0%)	0% (A: 0%)	0% (A: 0%)	0% (A: 0%)	0% (A: 0%)	0% (A: 0%)	0% (A: 0%)	----
	(15)	(6)	(37)	(8)	(2)	(0)	(19)	(11)	
4938 (G)	0%	0%	0%	0%	0%	0%	0%	0%	0.60
	0% (A: 0%)	0% (A: 0%)	0% (A: 0%)	0% (A: 0%)	0% (T: 0%)	0% (A: 0%)	0% (A: 0%)	0% (A: 0%)	0.50
	(413)	(86)	(807)	(229)	(207)	(14)	(392)	(191)	
4948 (A)	2%	4%	5%	16%	0%	0%	1%	0%	0.08
	0% (A: 0%)	1% (G: 1%)	0% (G: 0%)	0% (G: 0%)	0% (A: 0%)	0% (A: 0%)	1% (G: 1%)	2% (G: 2%)	0.42
	(458)	(97)	(874)	(236)	(211)	(14)	(414)	(185)	
5023 (C)	6%	5%	10%	15%	0%	0%	0%	0%	0.01
	0% (A: 0%)	1% (T: 1%)	0% (A: 0%)	0% (A: 0%)	0% (A: 0%)	0% (A: 0%)	0% (A: 0%)	0% (A: 0%)	0.36
	(157)	(77)	(120)	(52)	(17)	(11)	(110)	(72)	
5182 (A)	4%	0%	0%	0%	0%	0%	1%	0%	0.50
	0% (A: 0%)	0% (A: 0%)	0% (A: 0%)	0% (A: 0%)	0% (A: 0%)	0% (A: 0%)	1% (T: 1%)	1% (T: 1%)	0.14
	(70)	(35)	(66)	(18)	(2)	(1)	(94)	(77)	
5188 (A)	0%	0%	0%	0%	0%	0%	0%	0%	----
	0% (A: 0%)	0% (A: 0%)	0% (A: 0%)	0% (A: 0%)	0% (A: 0%)	0% (A: 0%)	0% (A: 0%)	0% (A: 0%)	----
	(75)	(36)	(65)	(18)	(2)	(2)	(97)	(77)	
5204 (T)	0%	6%	0%	0%	22%	0%	9%	12%	0.10
	0% (A: 0%)	0% (A: 0%)	0% (A: 0%)	0% (A: 0%)	0% (A: 0%)	0% (A: 0%)	0% (A: 0%)	0% (A: 0%)	----
	(113)	(28)	(80)	(18)	(7)	(6)	(132)	(88)	
5242 (A)	7%	9%	70%	85%	10%	20%	3%	1%	0.15
	0% (A: 0%)	0% (A: 0%)	0% (A: 0%)	0% (A: 0%)	0% (A: 0%)	0% (A: 0%)	0% (A: 0%)	0% (A: 0%)	----
	(145)	(46)	(250)	(100)	(34)	(8)	(310)	(203)	
5243 (C)	15%	17%	28%	29%	22%	0%	11%	8%	0.08
	0% (A: 0%)	0% (A: 0%)	0% (A: 0%)	0% (A: 0%)	0% (A: 0%)	0% (A: 0%)	0% (T: 0%)	0% (T: 0%)	0.15
	(157)	(51)	(848)	(680)	(38)	(10)	(322)	(206)	
5244 (A)	10%	1%	11%	12%	4%	0%	3%	2%	0.05
	0% (A: 0%)	0% (A: 0%)	0% (T: 0%)	0% (T: 0%)	0% (A: 0%)	0% (A: 0%)	0% (A: 0%)	0% (A: 0%)	0.14
	(183)	(56)	(1188)	(961)	(45)	(10)	(356)	(225)	
5257 (C)	3%	4%	0%	0%	1%	0%	54%	67%	0.16
	0% (T: 0%)	0% (A: 0%)	0% (T: 0%)	0% (T: 0%)	0% (A: 0%)	0% (A: 0%)	0% (T: 0%)	0% (T: 0%)	0.41
	(395)	(121)	(2035)	(1325)	(285)	(21)	(567)	(322)	
5300 (T)	5%	1%	10%	12%	2%	2%	2%	2%	0.08
	0% (A: 0%)	0% (A: 0%)	0% (A: 0%)	0% (A: 0%)	0% (A: 0%)	0% (A: 0%)	0% (G: 0%)	0% (G: 0%)	0.30
	(1097)	(236)	(2926)	(1446)	(299)	(36)	(1860)	(1411)	
5426 (T)	7%	4%	18%	20%	2%	0%	0%	0%	0.03
	0% (A: 0%)	0% (A: 0%)	0% (A: 0%)	0% (A: 0%)	0% (A: 0%)	0% (A: 0%)	0% (A: 0%)	0% (A: 0%)	----
	(250)	(73)	(237)	(112)	(43)	(7)	(913)	(805)	
5433 (T)	10%	5%	11%	0%	1%	0%	3%	0%	0.09
	0% (C: 0%)	0% (A: 0%)	0% (G: 0%)	0% (G: 0%)	0% (A: 0%)	0% (A: 0%)	0% (A: 0%)	0% (A: 0%)	0.04
	(323)	(76)	(391)	(211)	(61)	(12)	(989)	(839)	
5458 (A)	7%	5%	48%	71%	8%	0%	1%	0%	0.11
	0% (A: 0%)	0% (A: 0%)	0% (T: 0%)	0% (T: 0%)	0% (A: 0%)	0% (A: 0%)	0% (T: 0%)	0% (T: 0%)	0.47
	(584)	(93)	(885)	(321)	(66)	(15)	(1298)	(976)	
5512 (C)	19%	15%	4%	1%	41%	16%	2%	0%	0.62
	0% (A: 0%)	0% (A: 0%)	0% (A: 0%)	0% (A: 0%)	0% (A: 0%)	0% (A: 0%)	0% (A: 0%)	0% (A: 0%)	0.23
	(494)	(112)	(2133)	(1414)	(83)	(16)	(1244)	(974)	
5699 (T)	6%	17%	3%	0%	44%	7%	1%	0%	0.58
	0% (A: 0%)	0% (A: 0%)	0% (G: 0%)	0% (G: 0%)	0% (A: 0%)	3% (A: 3%)	0% (A: 0%)	1% (A: 0%)	0.20
	(474)	(94)	(843)	(329)	(98)	(26)	(725)	(472)	
5737 (T)	6%	2%	13%	18%	1%	4%	1%	1%	0.07
	0% (A: 0%)	0% (A: 0%)	0% (A: 0%)	0% (A: 0%)	0% (A: 0%)	0% (A: 0%)	0% (A: 0%)	0% (A: 0%)	0.96
	(596)	(156)	(1038)	(387)	(157)	(24)	(1412)	(1004)	
5791 (A)	3%	1%	14%	40%	1%	1%	1%	0%	0.18
	0% (T: 0%)	0% (A: 0%)	0% (A: 0%)	0% (A: 0%)	0% (A: 0%)	0% (A: 0%)	0% (T: 0%)	0% (T: 0%)	0.62
	(1200)	(279)	(1804)	(412)	(426)	(52)	(1675)	(1224)	
5911 (A)	3%	7%	1%	0%	0%	0%	1%	1%	0.17
	0% (T: 0%)	0% (A: 0%)	0% (T: 0%)	1% (T: 1%)	0% (A: 0%)	0% (A: 0%)	0% (T: 0%)	0% (T: 0%)	0.22
	(290)	(100)	(1375)	(728)	(33)	(12)	(836)	(588)	
5945 (T)	19%	13%	4%	0%	46%	31%	3%	0%	0.39
	0% (A: 0%)	0% (A: 0%)	0% (G: 0%)	0% (G: 0%)	0% (A: 0%)	0% (A: 0%)	0% (G: 0%)	0% (G: 0%)	0.64
	(476)	(127)	(1639)	(885)	(220)	(14)	(642)	(461)	
5986 (A)	1%	0%	14%	40%	0%	0%	0%	0%	0.19
	0% (G: 0%)	0% (G: 0%)	0% (G: 0%)	0% (G: 0%)	0% (A: 0%)	0% (A: 0%)	0% (G: 0%)	0% (G: 0%)	0.87
	(2355)	(488)	(2830)	(700)	(1812)	(140)	(1296)	(551)	
6037 (T)	0%	1%	0%	0%	0%	9%	4%	12%	0.07
	0% (C: 0%)	0% (A: 0%)	0% (A: 0%)	0% (A: 0%)	0% (A: 0%)	0% (A: 0%)	0% (A: 0%)	0% (A: 0%)	0.48
	(428)	(136)	(2061)	(1108)	(45)	(10)	(662)	(228)	
6081 (A)	1%	0%	0%	0%	0%	50%	0%	0%	0.37
	0% (A: 0%)	0% (A: 0%)	0% (G: 0%)	0% (G: 0%)	0% (A: 0%)	0% (A: 0%)	0% (G: 0%)	0% (G: 0%)	0.23
	(71)	(92)	(1028)	(935)	(6)	(3)	(211)	(160)	
6139 (A)	0%	0%	61%	75%	0%	10%	0%	0%	0.16
	0% (A: 0%)	0% (A: 0%)	0% (A: 0%)	0% (A: 0%)	0% (A: 0%)	0% (A: 0%)	0% (A: 0%)	0% (A: 0%)	----
	(24)	(187)	(54)	(27)	(4)	(9)	(57)	(30)	
6169 (A)	10%	3%	1%	0%	0%	0%	2%	0%	0.19
	0% (A: 0%)	0% (A: 0%)	0% (A: 0%)	0% (A: 0%)	0% (A: 0%)	0% (A: 0%)	0% (A: 0%)	0% (A: 0%)	----
	(9)	(272)	(177)	(166)	(0)	(12)	(46)	(40)	
6197 (T)	48%	3%	7%	0%	52%	2%	9%	5%	0.88
	0% (A: 0%)	0% (A: 0%)	0% (A: 0%)	0% (A: 0%)	0% (A: 0%)	0% (A: 0%)	0% (A: 0%)	0% (A: 0%)	----
	(65)	(332)	(438)	(368)	(11)	(33)	(159)	(127)	
6315 (A)	2%	0%	0%	0%	7%	25%	9%	13%	0.02
	0% (A: 0%)	0% (A: 0%)	0% (G: 0%)	1% (G: 1%)	1% (C: 1%)	0% (A: 0%)	2% (G: 1%)	2% (G: 2%)	0.13
	(283)	(87)	(965)	(560)	(67)	(8)	(296)	(169)	
6409 (A)	11%	3%	6%	7%	6%	0%	1%	0%	0.08
	0% (A: 0%)	0% (A: 0%)	0% (A: 0%)	0% (A: 0%)	0% (A: 0%)	0% (A: 0%)	0% (A: 0%)	0% (A: 0%)	----
	(247)	(85)	(871)	(667)	(133)	(14)	(521)	(431)	
6510 (A)	2%	3%	0%	0%	0%	9%	0%	0%	0.82
	0% (A: 0%)	0% (A: 0%)	0% (G: 0%)	0% (G: 0%)	0% (A: 0%)	0% (A: 0%)	0% (A: 0%)	0% (A: 0%)	0.14
	(314)	(101)	(845)	(576)	(29)	(10)	(633)	(533)	
6514 (T)	3%	1%	18%	23%	0%	0%	0%	0%	0.08
	0% (A: 0%)	1% (G: 1%)	1% (G: 1%)	2% (G: 1%)	0% (A: 0%)	0% (A: 0%)	0% (G: 0%)	1% (G: 1%)	0.32
	(311)	(99)	(847)	(591)	(25)	(11)	(607)	(512)	
6562 (A)	1%	3%	0%	0%	0%	6%	0%	0%	0.90
	0% (A: 0%)	0% (A: 0%)	0% (A: 0%)	0% (A: 0%)	0% (A: 0%)	0% (A: 0%)	0% (A: 0%)	0% (A: 0%)	----
	(202)	(53)	(587)	(444)	(16)	(15)	(221)	(159)	
6570 (A)	0%	0%	0%	0%	0%	6%	20%	25%	0.07
	0% (A: 0%)	0% (A: 0%)	0% (A: 0%)	0% (A: 0%)	0% (A: 0%)	0% (A: 0%)	0% (G: 0%)	1% (G: 1%)	0.14
	(198)	(50)	(569)	(427)	(16)	(16)	(227)	(168)	
6619 (T)	3%	4%	7%	13%	0%	0%	1%	0%	0.03
	0% (A: 0%)	0% (A: 0%)	0% (A: 0%)	0% (A: 0%)	0% (A: 0%)	0% (A: 0%)	1% (A: 1%)	0% (A: 0%)	0.14
	(156)	(91)	(239)	(114)	(13)	(21)	(243)	(212)	
6752 (A)	7%	5%	0%	0%	0%	0%	25%	30%	0.24
	0% (A: 0%)	0% (A: 0%)	0% (A: 0%)	0% (A: 0%)	0% (A: 0%)	0% (A: 0%)	0% (A: 0%)	0% (A: 0%)	----
	(56)	(39)	(47)	(17)	(3)	(0)	(74)	(56)	
6795 (T)	0%	4%	14%	24%	0%	0%	46%	57%	0.38
	0% (A: 0%)	0% (A: 0%)	0% (A: 0%)	0% (A: 0%)	0% (A: 0%)	0% (A: 0%)	0% (A: 0%)	0% (A: 0%)	----
	(139)	(65)	(295)	(142)	(11)	(5)	(189)	(125)	
6797 (A)	2%	0%	0%	0%	0%	28%	11%	13%	0.07
	0% (A: 0%)	0% (A: 0%)	0% (A: 0%)	0% (A: 0%)	0% (A: 0%)	0% (A: 0%)	0% (A: 0%)	0% (A: 0%)	----
	(138)	(69)	(344)	(187)	(14)	(5)	(365)	(299)	
6901 (T)	46%	11%	11%	0%	21%	0%	3%	0%	0.36
	0% (A: 0%)	0% (A: 0%)	0% (A: 0%)	0% (A: 0%)	0% (A: 0%)	0% (A: 0%)	0% (A: 0%)	0% (A: 0%)	0.14
	(99)	(83)	(822)	(722)	(13)	(10)	(920)	(829)	
6907 (G)	0%	0%	0%	0%	5%	0%	13%	14%	0.06
	0% (A: 0%)	0% (A: 0%)	0% (A: 0%)	0% (A: 0%)	0% (A: 0%)	0% (A: 0%)	0% (A: 0%)	0% (A: 0%)	0.82
	(285)	(101)	(1024)	(660)	(18)	(10)	(905)	(770)	
6913 (C)	8%	6%	4%	1%	5%	0%	2%	0%	0.18
	0% (A: 0%)	0% (A: 0%)	32% (T: 32%)	56% (T: 55%)	0% (A: 0%)	0% (A: 0%)	0% (T: 0%)	0% (T: 0%)	0.16
	(297)	(109)	(884)	(504)	(18)	(13)	(877)	(728)	
6915 (A)	2%	0%	0%	0%	4%	12%	0%	0%	0.29
	0% (A: 0%)	0% (A: 0%)	0% (A: 0%)	0% (A: 0%)	0% (A: 0%)	0% (A: 0%)	0% (A: 0%)	0% (A: 0%)	----
	(325)	(117)	(927)	(506)	(21)	(14)	(786)	(627)	
6964 (A)	4%	2%	25%	41%	0%	5%	2%	0%	0.13
	0% (A: 0%)	0% (A: 0%)	0% (A: 0%)	0% (A: 0%)	0% (A: 0%)	0% (A: 0%)	0% (A: 0%)	0% (A: 0%)	----
	(352)	(112)	(834)	(389)	(35)	(18)	(844)	(599)	
6999 (A)	4%	5%	0%	0%	3%	10%	17%	24%	0.05
	0% (A: 0%)	0% (A: 0%)	0% (T: 0%)	1% (T: 0%)	0% (A: 0%)	0% (A: 0%)	0% (G: 0%)	1% (G: 0%)	0.94
	(250)	(83)	(749)	(410)	(25)	(17)	(505)	(326)	
7106 (A)	3%	0%	0%	0%	12%	12%	0%	0%	0.22
	0% (A: 0%)	0% (A: 0%)	0% (A: 0%)	0% (A: 0%)	11% (G: 11%)	0% (A: 0%)	0% (A: 0%)	0% (A: 0%)	0.36
	(26)	(129)	(63)	(50)	(9)	(7)	(57)	(45)	
7132 (T)	24%	3%	43%	0%	15%	2%	26%	25%	0.97
	0% (A: 0%)	0% (A: 0%)	0% (A: 0%)	0% (A: 0%)	0% (A: 0%)	0% (A: 0%)	0% (A: 0%)	0% (A: 0%)	----
	(104)	(150)	(61)	(3)	(71)	(44)	(35)	(8)	
7169 (A)	0%	0%	0%	0%	0%	0%	0%	0%	0.16
	0% (A: 0%)	0% (T: 0%)	0% (A: 0%)	0% (A: 0%)	0% (A: 0%)	0% (A: 0%)	1% (G: 1%)	0% (A: 0%)	0.55
	(114)	(209)	(81)	(2)	(20)	(26)	(68)	(23)	
7178 (A)	0%	1%	0%	0%	0%	5%	0%	0%	0.52
	0% (A: 0%)	0% (A: 0%)	0% (A: 0%)	0% (A: 0%)	0% (A: 0%)	0% (A: 0%)	0% (A: 0%)	0% (A: 0%)	----
	(73)	(193)	(57)	(2)	(11)	(19)	(57)	(23)	
7207 (T)	9%	2%	0%	0%	0%	0%	0%	0%	0.23
	0% (A: 0%)	0% (A: 0%)	0% (A: 0%)	0% (A: 0%)	0% (A: 0%)	0% (A: 0%)	0% (A: 0%)	0% (A: 0%)	----
	(22)	(122)	(14)	(0)	(3)	(17)	(11)	(3)	
7227 (G)	0%	0%	0%	0%	0%	0%	0%	0%	----
	0% (A: 0%)	0% (A: 0%)	0% (A: 0%)	0% (A: 0%)	0% (A: 0%)	0% (A: 0%)	0% (A: 0%)	0% (A: 0%)	----
	(29)	(147)	(31)	(3)	(2)	(12)	(6)	(1)	
7247 (A)	0%	0%	0%	0%	0%	0%	0%	0%	0.36
	0% (A: 0%)	0% (G: 0%)	0% (A: 0%)	0% (A: 0%)	0% (A: 0%)	0% (A: 0%)	0% (A: 0%)	0% (A: 0%)	0.36
	(26)	(160)	(31)	(3)	(1)	(14)	(7)	(1)	
7339 (T)	0%	1%	0%	0%	0%	14%	15%	30%	0.06
	0% (A: 0%)	0% (A: 0%)	0% (A: 0%)	0% (A: 0%)	0% (A: 0%)	0% (A: 0%)	0% (A: 0%)	0% (A: 0%)	----
	(118)	(60)	(257)	(125)	(15)	(8)	(86)	(37)	
7345 (T)	0%	1%	34%	46%	0%	0%	7%	5%	0.19
	0% (A: 0%)	0% (A: 0%)	0% (A: 0%)	0% (A: 0%)	0% (A: 0%)	0% (A: 0%)	0% (A: 0%)	0% (A: 0%)	----
	(112)	(58)	(285)	(168)	(10)	(6)	(89)	(50)	
7376 (A)	2%	18%	1%	0%	0%	0%	1%	0%	0.25
	0% (A: 0%)	0% (A: 0%)	0% (C: 0%)	0% (A: 0%)	0% (A: 0%)	0% (A: 0%)	0% (A: 0%)	0% (A: 0%)	0.36
	(65)	(41)	(443)	(367)	(6)	(6)	(145)	(103)	
7386 (T)	2%	2%	0%	0%	0%	25%	32%	39%	0.04
	0% (A: 0%)	0% (A: 0%)	0% (A: 0%)	0% (A: 0%)	0% (A: 0%)	0% (A: 0%)	0% (A: 0%)	0% (A: 0%)	----
	(69)	(47)	(478)	(409)	(7)	(6)	(138)	(101)	
7478 (C)	5%	2%	7%	13%	0%	0%	0%	0%	0.02
	0% (A: 0%)	0% (A: 0%)	0% (A: 0%)	0% (A: 0%)	0% (A: 0%)	0% (A: 0%)	0% (A: 0%)	0% (A: 0%)	----
	(161)	(118)	(500)	(349)	(10)	(17)	(173)	(91)	
7485 (T)	11%	4%	7%	0%	27%	0%	24%	34%	0.09
	0% (A: 0%)	0% (A: 0%)	0% (A: 0%)	0% (A: 0%)	0% (A: 0%)	0% (A: 0%)	0% (A: 0%)	0% (A: 0%)	0.17
	(151)	(110)	(238)	(116)	(8)	(16)	(199)	(115)	
7519 (T)	0%	0%	0%	0%	2%	5%	11%	13%	0.02
	0% (A: 0%)	0% (A: 0%)	0% (A: 0%)	0% (A: 0%)	0% (A: 0%)	0% (A: 0%)	0% (C: 0%)	0% (A: 0%)	0.36
	(351)	(142)	(497)	(200)	(38)	(17)	(342)	(254)	
7543 (T)	9%	1%	24%	26%	0%	0%	1%	0%	0.05
	0% (A: 0%)	0% (A: 0%)	0% (A: 0%)	0% (A: 0%)	0% (A: 0%)	0% (A: 0%)	0% (A: 0%)	0% (A: 0%)	----
	(149)	(95)	(339)	(235)	(15)	(8)	(504)	(418)	
7578 (C)	10%	6%	16%	13%	9%	6%	4%	3%	0.05
	0% (A: 0%)	0% (A: 0%)	0% (A: 0%)	0% (A: 0%)	0% (A: 0%)	0% (A: 0%)	0% (A: 0%)	0% (A: 0%)	----
	(258)	(155)	(1251)	(940)	(29)	(15)	(733)	(564)	
7594 (T)	16%	6%	4%	2%	43%	6%	1%	0%	0.62
	0% (A: 0%)	0% (A: 0%)	0% (A: 0%)	0% (A: 0%)	0% (A: 0%)	0% (A: 0%)	0% (A: 0%)	0% (G: 0%)	0.61
	(394)	(225)	(1768)	(1216)	(87)	(29)	(919)	(685)	
7671 (A)	0%	1%	2%	2%	25%	0%	1%	1%	0.42
	0% (A: 0%)	0% (A: 0%)	0% (C: 0%)	0% (C: 0%)	0% (A: 0%)	0% (A: 0%)	0% (A: 0%)	0% (A: 0%)	0.14
	(45)	(287)	(526)	(377)	(3)	(11)	(367)	(278)	
7678 (A)	1%	3%	14%	19%	0%	0%	1%	0%	0.08
	0% (A: 0%)	0% (G: 0%)	0% (G: 0%)	0% (G: 0%)	0% (A: 0%)	0% (A: 0%)	0% (A: 0%)	0% (A: 0%)	0.36
	(74)	(328)	(630)	(392)	(14)	(18)	(375)	(289)	
7680 (T)	19%	5%	14%	10%	36%	10%	25%	29%	0.08
	0% (A: 0%)	0% (A: 0%)	0% (G: 0%)	0% (G: 0%)	0% (A: 0%)	0% (A: 0%)	1% (A: 1%)	1% (A: 0%)	0.54
	(74)	(325)	(736)	(488)	(14)	(18)	(375)	(287)	
7681 (A)	3%	10%	7%	8%	8%	13%	10%	12%	0.07
	0% (A: 0%)	1% (T: 0%)	0% (T: 0%)	0% (T: 0%)	0% (A: 0%)	0% (A: 0%)	0% (T: 0%)	0% (T: 0%)	0.78
	(87)	(333)	(843)	(540)	(22)	(19)	(491)	(394)	
7684 (A)	11%	3%	5%	4%	9%	15%	11%	12%	0.03
	0% (A: 0%)	0% (A: 0%)	0% (A: 0%)	0% (A: 0%)	0% (A: 0%)	0% (A: 0%)	0% (A: 0%)	0% (A: 0%)	----
	(109)	(390)	(1129)	(734)	(30)	(27)	(536)	(428)	
7693 (A)	3%	0%	4%	5%	16%	3%	8%	10%	0.07
	0% (A: 0%)	0% (A: 0%)	0% (T: 0%)	0% (T: 0%)	0% (A: 0%)	0% (A: 0%)	0% (C: 0%)	0% (C: 0%)	0.26
	(200)	(443)	(1546)	(1012)	(84)	(49)	(691)	(562)	
7741 (A)	0%	1%	1%	1%	0%	2%	0%	0%	0.27
	0% (G: 0%)	0% (G: 0%)	0% (G: 0%)	0% (G: 0%)	0% (A: 0%)	0% (A: 0%)	0% (G: 0%)	0% (G: 0%)	0.87
	(927)	(582)	(7017)	(4814)	(488)	(87)	(2383)	(1789)	
7767 (T)	1%	2%	0%	1%	7%	0%	11%	15%	0.07
	0% (A: 0%)	0% (A: 0%)	0% (G: 0%)	0% (G: 0%)	0% (A: 0%)	0% (A: 0%)	0% (G: 0%)	0% (G: 0%)	0.92
	(683)	(329)	(8586)	(6162)	(301)	(38)	(2521)	(1826)	
7873 (C)	6%	5%	13%	11%	2%	3%	1%	0%	0.01
	0% (A: 0%)	0% (A: 0%)	0% (A: 0%)	0% (A: 0%)	0% (A: 0%)	0% (A: 0%)	0% (A: 0%)	0% (A: 0%)	0.16
	(514)	(298)	(2032)	(1315)	(164)	(57)	(1283)	(744)	
7904 (C)	16%	6%	29%	17%	7%	2%	7%	3%	0.05
	0% (A: 0%)	0% (A: 0%)	0% (A: 0%)	0% (A: 0%)	0% (A: 0%)	0% (A: 0%)	0% (A: 0%)	0% (A: 0%)	0.36
	(674)	(304)	(1535)	(629)	(392)	(106)	(1028)	(389)	
7918 (C)	6%	3%	12%	21%	6%	0%	2%	0%	0.09
	0% (A: 0%)	0% (A: 0%)	0% (A: 0%)	0% (A: 0%)	0% (A: 0%)	0% (A: 0%)	0% (A: 0%)	0% (T: 0%)	0.12
	(854)	(402)	(2418)	(842)	(556)	(122)	(1347)	(501)	
7944 (A)	1%	0%	1%	1%	0%	1%	2%	1%	0.54
	0% (A: 0%)	0% (A: 0%)	0% (G: 0%)	0% (G: 0%)	0% (C: 0%)	0% (A: 0%)	0% (G: 0%)	0% (G: 0%)	0.66
	(974)	(570)	(4498)	(2632)	(922)	(148)	(1770)	(980)	
7974 (T)	5%	3%	12%	12%	2%	0%	0%	0%	0.02
	0% (A: 0%)	0% (A: 0%)	0% (C: 0%)	1% (C: 1%)	0% (A: 0%)	0% (A: 0%)	0% (C: 0%)	0% (C: 0%)	0.51
	(500)	(345)	(5594)	(4069)	(303)	(50)	(1816)	(1159)	
7990 (C)	5%	7%	2%	0%	2%	14%	11%	12%	0.08
	0% (A: 0%)	0% (A: 0%)	0% (T: 0%)	0% (T: 0%)	0% (A: 0%)	0% (A: 0%)	0% (G: 0%)	0% (G: 0%)	0.23
	(493)	(324)	(7632)	(5526)	(186)	(46)	(2197)	(1548)	
8020 (C)	1%	0%	8%	10%	0%	0%	0%	0%	0.07
	0% (A: 0%)	0% (G: 0%)	0% (G: 0%)	0% (G: 0%)	0% (A: 0%)	0% (A: 0%)	0% (G: 0%)	1% (G: 0%)	0.93
	(501)	(224)	(7973)	(6063)	(75)	(48)	(2786)	(2118)	
8151 (T)	52%	16%	1%	0%	76%	48%	2%	0%	0.55
	0% (A: 0%)	0% (A: 0%)	1% (G: 1%)	0% (G: 0%)	0% (A: 0%)	0% (A: 0%)	0% (A: 0%)	0% (A: 0%)	0.14
	(61)	(239)	(1478)	(1055)	(3)	(16)	(885)	(704)	
8274 (T)	3%	1%	4%	1%	40%	23%	7%	10%	0.06
	0% (A: 0%)	0% (A: 0%)	0% (A: 0%)	0% (A: 0%)	0% (A: 0%)	0% (A: 0%)	0% (A: 0%)	0% (A: 0%)	----
	(81)	(208)	(763)	(556)	(3)	(10)	(338)	(212)	
8300 (A)	3%	0%	1%	2%	9%	0%	8%	11%	0.09
	0% (A: 0%)	0% (A: 0%)	0% (G: 0%)	0% (G: 0%)	0% (A: 0%)	0% (A: 0%)	0% (A: 0%)	0% (A: 0%)	0.15
	(179)	(211)	(1399)	(963)	(10)	(30)	(756)	(544)	
8352 (C)	2%	0%	0%	0%	10%	16%	2%	2%	0.08
	0% (A: 0%)	0% (A: 0%)	0% (T: 0%)	0% (T: 0%)	0% (A: 0%)	0% (A: 0%)	0% (T: 0%)	0% (T: 0%)	0.80
	(290)	(157)	(4300)	(3738)	(108)	(42)	(2523)	(2242)	
8365 (T)	24%	29%	1%	1%	54%	7%	1%	1%	0.89
	0% (A: 0%)	0% (A: 0%)	1% (C: 0%)	1% (C: 0%)	0% (A: 0%)	0% (A: 0%)	0% (C: 0%)	0% (C: 0%)	0.84
	(326)	(194)	(4928)	(4433)	(296)	(62)	(2905)	(2673)	
8367 (A)	5%	7%	1%	0%	10%	9%	4%	4%	0.17
	0% (A: 0%)	0% (A: 0%)	0% (C: 0%)	0% (C: 0%)	0% (G: 0%)	0% (A: 0%)	0% (C: 0%)	0% (C: 0%)	0.97
	(505)	(324)	(5320)	(4785)	(815)	(77)	(2939)	(2711)	
8450 (A)	2%	0%	0%	0%	0%	0%	0%	0%	0.42
	0% (C: 0%)	0% (A: 0%)	0% (C: 0%)	0% (C: 0%)	0% (A: 0%)	0% (A: 0%)	0% (C: 0%)	0% (C: 0%)	0.83
	(1010)	(451)	(5522)	(3795)	(322)	(158)	(2883)	(2095)	
8479 (T)	31%	24%	16%	0%	52%	27%	9%	0%	0.77
	0% (C: 0%)	0% (C: 0%)	0% (A: 0%)	0% (A: 0%)	0% (A: 0%)	0% (A: 0%)	0% (A: 0%)	0% (A: 0%)	0.87
	(1239)	(384)	(6519)	(3965)	(336)	(125)	(3450)	(2533)	
8661 (A)	1%	1%	0%	0%	3%	2%	0%	0%	0.51
	0% (A: 0%)	0% (A: 0%)	0% (T: 0%)	0% (T: 0%)	0% (A: 0%)	0% (A: 0%)	0% (T: 0%)	0% (T: 0%)	0.27
	(850)	(308)	(6077)	(4393)	(211)	(67)	(2788)	(2048)	
8758 (A)	4%	3%	18%	20%	2%	5%	0%	0%	0.08
	0% (A: 0%)	0% (A: 0%)	0% (G: 0%)	0% (G: 0%)	0% (A: 0%)	0% (A: 0%)	0% (A: 0%)	0% (A: 0%)	0.22
	(434)	(343)	(8051)	(6974)	(69)	(59)	(4046)	(3511)	
8759 (C)	4%	2%	9%	10%	1%	1%	3%	3%	0.08
	0% (A: 0%)	0% (A: 0%)	0% (G: 0%)	0% (G: 0%)	0% (A: 0%)	0% (A: 0%)	0% (A: 0%)	0% (A: 0%)	0.75
	(451)	(353)	(9824)	(8718)	(70)	(60)	(4058)	(3502)	
8993 (A)	0%	0%	0%	0%	0%	2%	0%	0%	0.25
	0% (A: 0%)	0% (A: 0%)	0% (G: 0%)	0% (G: 0%)	0% (A: 0%)	0% (A: 0%)	0% (G: 0%)	0% (G: 0%)	0.32
	(881)	(363)	(5326)	(2939)	(370)	(75)	(4229)	(3289)	
9058 (T)	30%	15%	12%	0%	62%	9%	3%	0%	0.80
	0% (A: 0%)	0% (A: 0%)	0% (A: 0%)	1% (G: 0%)	0% (A: 0%)	0% (A: 0%)	0% (A: 0%)	0% (A: 0%)	0.59
	(339)	(251)	(2059)	(1134)	(69)	(33)	(1259)	(910)	
9072 (G)	0%	0%	21%	42%	0%	2%	1%	0%	0.17
	0% (T: 0%)	0% (A: 0%)	0% (A: 0%)	0% (A: 0%)	0% (T: 0%)	0% (A: 0%)	0% (A: 0%)	0% (A: 0%)	0.22
	(1199)	(509)	(2951)	(1053)	(621)	(97)	(1357)	(750)	
9128 (C)	0%	0%	33%	40%	1%	1%	2%	2%	0.17
	0% (A: 0%)	1% (A: 1%)	0% (T: 0%)	0% (T: 0%)	0% (A: 0%)	0% (A: 0%)	0% (A: 0%)	0% (A: 0%)	0.29
	(651)	(309)	(4463)	(3268)	(195)	(57)	(1390)	(832)	
9241 (A)	3%	2%	1%	1%	8%	3%	15%	18%	0.05
	0% (G: 0%)	0% (G: 0%)	0% (G: 0%)	0% (G: 0%)	0% (G: 0%)	0% (A: 0%)	0% (G: 0%)	0% (G: 0%)	0.08
	(1195)	(679)	(12216)	(9901)	(399)	(128)	(5355)	(4253)	
9283 (A)	0%	0%	0%	0%	1%	1%	2%	2%	0.03
	0% (C: 0%)	0% (A: 0%)	0% (G: 0%)	0% (G: 0%)	0% (A: 0%)	0% (A: 0%)	0% (G: 0%)	0% (G: 0%)	0.91
	(1330)	(536)	(12209)	(9835)	(542)	(85)	(7738)	(6784)	
9396 (A)	2%	0%	0%	0%	2%	1%	7%	10%	0.07
	0% (A: 0%)	0% (A: 0%)	0% (G: 0%)	0% (C: 0%)	0% (A: 0%)	0% (A: 0%)	0% (C: 0%)	0% (C: 0%)	0.39
	(1134)	(497)	(6963)	(4850)	(518)	(100)	(2303)	(1352)	
9528 (T)	44%	61%	23%	9%	27%	42%	6%	0%	0.35
	1% (G: 1%)	6% (G: 5%)	1% (G: 1%)	8% (G: 8%)	1% (G: 1%)	2% (G: 2%)	0% (G: 0%)	0% (G: 0%)	0.10
	(366)	(320)	(706)	(99)	(150)	(39)	(917)	(592)	
9529 (G)	3%	3%	16%	62%	2%	0%	0%	1%	0.19
	0% (C: 0%)	0% (C: 0%)	0% (A: 0%)	0% (A: 0%)	0% (A: 0%)	0% (A: 0%)	0% (A: 0%)	0% (A: 0%)	0.65
	(637)	(799)	(910)	(109)	(198)	(64)	(968)	(592)	
9624 (T)	3%	6%	1%	0%	0%	44%	66%	67%	0.04
	0% (A: 0%)	0% (A: 0%)	0% (A: 0%)	1% (A: 0%)	20% (G: 20%)	0% (A: 0%)	1% (C: 0%)	1% (C: 0%)	0.32
	(30)	(31)	(416)	(376)	(5)	(5)	(312)	(302)	
9626 (A)	42%	45%	7%	2%	56%	40%	4%	2%	0.94
	0% (A: 0%)	0% (A: 0%)	0% (A: 0%)	0% (A: 0%)	0% (A: 0%)	0% (A: 0%)	0% (A: 0%)	0% (A: 0%)	----
	(46)	(62)	(443)	(379)	(7)	(27)	(946)	(923)	
9627 (A)	60%	35%	11%	2%	42%	37%	7%	1%	0.77
	0% (A: 0%)	0% (A: 0%)	0% (A: 0%)	0% (A: 0%)	0% (A: 0%)	0% (A: 0%)	0% (C: 0%)	0% (C: 0%)	0.13
	(80)	(113)	(480)	(390)	(16)	(45)	(989)	(949)	
9639 (C)	20%	26%	4%	0%	57%	18%	2%	0%	0.67
	0% (A: 0%)	0% (G: 0%)	0% (A: 0%)	0% (A: 0%)	0% (A: 0%)	1% (A: 1%)	0% (A: 0%)	0% (T: 0%)	0.22
	(597)	(563)	(1045)	(440)	(183)	(233)	(1490)	(1080)	

Overall statistics

	Lib 1 m^{1}A	Lib 2 m^{1}A	Lib 3 m^{1}A	Lib 4 m^{1}A-T	Lib 1 m^{6}A	Lib 2 m^{6}A	Lib 3 m^{6}A	Lib 4 m^{6}A-T
Total reads	109577	81169	339961	195284	58063	21105	164197	103256
Average coverage	354 (σ=550)	320 (σ=658)	2058 (σ=4713)	1388 (σ=3442)	181 (σ=437)	61 (σ=132)	900 (σ=1671)	639 (σ=1210)
Average errors	0 (σ=1)	0 (σ=4)	5 (σ=35)	5 (σ=33)	0 (σ=0)	0 (σ=1)	3 (σ=12)	2 (σ=12)
Total errors	0.12%	0.21%	0.28%	0.37%	0.1%	0.14%	0.35%	0.44%
Total "A" errors	0.03%	0.06%	0.1%	0.13%	0.03%	0.04%	0.12%	0.15%

HIV NL43 (Lib 1 m^{1}A)

HIV NL43 (Lib 2 m^{1}A)

HIV NL43 (Lib 3 m^{1}A)

HIV NL43 (Lib 4 m^{1}A-T)

HIV NL43 (Lib 1 m^{6}A)

HIV NL43 (Lib 2 m^{6}A)

HIV NL43 (Lib 3 m^{6}A)

HIV NL43 (Lib 4 m^{6}A-T)
